# Supplementary material for: Differential contribution of TFE3 isoforms to cell motility and invasion
Source: EMBO Rep. 2025 Dec 8;27(2):471–500. doi: 10.1038/s44319-025-00659-3 (PMC12852735; doi:10.1038/s44319-025-00659-3)
Supplement: Supplementary file 1 — Table EV1 [file 44319_2025_659_MOESM1_ESM.pdf]

Table EV1: PCR, RT-qPCR primers and siRNA probes.

REAGENT or RESOURCE	SOURCE	IDENTIFIER
Oligonucleotides		
**TFE3-Ser47A Forward**<br>GAACTCTTTGCTTCCGGAAGCCGGGATTGTTG<br>CTGACATAG	Eurofins Genomics	N/A
**TFE3-Ser47A Reverse**<br>CTATGTCAGCAACAATCCCGGCTTCCGGAAGC<br>AAAGAGTTC	Eurofins Genomics	N/A
**TFE3-M1A Forward**<br>GCCCGGGCGGATCCGCCACCGCTTCTCATGCG<br>GCCGAACCAG	Eurofins Genomics	N/A
**TFE3-M1A Reverse**<br>CTGGTTCGGCCGCATGAGAAGCGGTGGCGGAT<br>CCGCCCGGGC	Eurofins Genomics	N/A
**TFE3-M106A Forward**<br>GCTCCAGGACCCCTGCCGCGTCGTCATCTTCTT<br>CATCG	Eurofins Genomics	N/A
**TFE3-M106A Reverse**<br>CGATGAAGAAGATGACGACGCGGCAGGGGTC<br>CTGGAGC	Eurofins Genomics	N/A
**TFE3-R356-358A Forward**<br>GAAAGACAATCACAACCTAATTGAGGCTGCC<br>GCGCGATTCAACATTAACGACAGGATC	Eurofins Genomics	N/A
**TFE3-R356-358A Reverse**<br>GATCCTGTCGTTAATGTTGAATCGCGCGGCAG<br>CCTCAATTAGGTTGTGATTGTCTTTC	Eurofins Genomics	N/A
**TFE3-5UTR Forward (Suppl. Fig. 2B, Red)**<br>GCTAGCTCCATGGCTTAGCGGAGGAG	Eurofins Genomics	N/A
**TFE3-5UTR Forward (Suppl. Fig. 2B, Blue)**<br>CTTATTTTGTTAGGGGGACCGGGCCGAGGCCC<br>GAC	Eurofins Genomics	N/A
**TFE3-5UTR Forward (Suppl. Fig. 2B, Orange)**<br>GGCTTGGGATGAGACTTTTAGCTTACC	Eurofins Genomics	N/A
**TFE3-5UTR Forward (Suppl. Fig. 2B, Green)**<br>CCTAAAATATGGCCCCATATTTCCCATTCCAC<br>AG	Eurofins Genomics	N/A
**TFE3-Exon4 Reverse (Suppl. Fig. 2B, Black)**<br>GCTGGACCCGATGGTGAGCAGCGCCATG	Eurofins Genomics	N/A
**TFE3-Exon3-4 Forward (Suppl. Fig. 2C)**<br>CAGCTACACTCTCTGCATCGTCTTCTGC	Eurofins Genomics	N/A
**TFE3-Exon3-4 Reverse (Suppl. Fig. 2C)**<br>CCTTCTCTGAGCTGGACCCGATGGTGAGC	Eurofins Genomics	N/A

**TFE3 Exon8-9 Forward** CATTAACGACAGGATCAAGG	Eurofins Genomics	N/A
**TFE3 Exon8-9 Reverse** TGCTGCTCCTTCTGCAGCTT	Eurofins Genomics	N/A
**SNHG15 Forward** CACAAGAGTGCCTGCCATC	Eurofins Genomics	N/A
**SNHG15 Reverse** GGCAGCCACTGAAGGTATC	Eurofins Genomics	N/A
Alt-R® CRISPR-Cas9 Negative Control crRNA #1	IDT	Cat# 1072544
Alt-R CRISPR-Cas9 crRNA, TFE3-L TGTTCGTGCTGTTGGAGGAG	IDT	Cat# Hs.Cas9.TFE3.1.AK
Alt-R® CRISPR-Cas9 tracrRNA	IDT	Cat# 1072533
ON-TARGETplus Non-targeting Control Pool	Horizon Discovery	Cat# D-001810
ON-TARGETplus Human FLCN siRNA	Horizon Discovery	Cat# L-009998
ON-TARGETplus Human ATG7 siRNA	Horizon Discovery	Cat# L-020112
Hs_GAPDH_1_SG QuantiTect Primer Assay	Qiagen	Cat# QT00079247
Hs_ITGAX_1_SG QuantiTect Primer Assay	Qiagen	Cat# QT00030646
Hs_ACP5_1_SG QuantiTect Primer Assay	Qiagen	Cat# QT00199801
Hs_CHI3L1_1_SG QuantiTect Primer Assay	Qiagen	Cat# QT00066703
Hs_CDKN1C_1_SG QuantiTect Primer Assay	Qiagen	Cat# QT00018018
Hs_PMEL_1_SG QuantiTect Primer Assay	Qiagen	Cat# QT00016149
Hs_TYR_1_SG QuantiTect Primer Assay	Qiagen	Cat# QT00080815
Hs_GPR143_1_SG QuantiTect Primer Assay	Qiagen	Cat# QT00012635
Hs_ATP6V0A1_1_SG QuantiTect Primer Assay	Qiagen	Cat# QT00071666
Hs_CTNS_1_SG QuantiTect Primer Assay	Qiagen	Cat# QT00046914
Hs_MAP1LC3B_1_SG QuantiTect Primer Assay	Qiagen	Cat# QT00055069
Hs_WDR81_1_SG QuantiTect Primer Assay	Qiagen	Cat# QT00034496
Hs_ATG9B_1_SG QuantiTect Primer Assay	Qiagen	Cat# QT00242669
Hs_ATG4A_1_SG QuantiTect Primer Assay	Qiagen	Cat# QT00003423
Hs_KREMEN2_1_SG QuantiTect Primer Assay	Qiagen	Cat# QT00091385
Hs_ZNRF3_1_SG QuantiTect Primer Assay	Qiagen	Cat# QT00025802
Hs_TBX6_1_SG QuantiTect Primer Assay	Qiagen	Cat# QT00041741
Hs_CITED1_1_SG QuantiTect Primer Assay	Qiagen	Cat# QT00009086
Hs_CEBPA_1_SG QuantiTect Primer Assay	Qiagen	Cat# QT00203357
Hs_HEY2_1_SG QuantiTect Primer Assay	Qiagen	Cat# QT00026971
Hs_SCRT1_1_SG QuantiTect Primer Assay	Qiagen	Cat# QT00227332
Hs_ETV4_1_SG QuantiTect Primer Assay	Qiagen	Cat# QT00053060
Hs_BRCA1_1_SG QuantiTect Primer Assay	Qiagen	Cat# QT00039305
Hs_SLC45A4_1_SG QuantiTect Primer Assay	Qiagen	Cat# QT00218652
Hs_ROS1_1_SG QuantiTect Primer Assay	Qiagen	Cat# QT00000686
Hs_ACE2_1_SG QuantiTect Primer Assay	Qiagen	Cat# QT00034055
Hs_TFE3_1_SG QuantiTect Primer Assay	Qiagen	Cat# QT00041076
Hs_AXIN2_1_SG QuantiTect Primer Assay	Qiagen	Cat# QT00037639
Hs_TLE3_1_SG QuantiTect Primer Assay	Qiagen	Cat# QT00045003
Hs_NKD1_1_SG QuantiTect Primer Assay	Qiagen	Cat# QT00036106
Hs_DKK2_1_SG QuantiTect Primer Assay	Qiagen	Cat# QT00038073

Hs_JUP_1_SG QuantiTect Primer Assay	Qiagen	Cat# QT00089166
Hs_GPNMB_1_SG QuantiTect Primer Assay	Qiagen	Cat# QT00036904
Hs_ATP6V1C1_1_SG QuantiTect Primer Assay	Qiagen	Cat# QT00015022
Hs_UVRAG_1_SG QuantiTect Primer Assay	Qiagen	Cat# QT00034328
Hs_GLA_1_SG QuantiTect Primer Assay	Qiagen	Cat# QT00012047
Hs_LAMP1_1_SG QuantiTect Primer Assay	Qiagen	Cat# QT00070994
Hs_CTSA_1_SG QuantiTect Primer Assay	Qiagen	Cat# QT00087381
Hs_MCOLN1_1_SG QuantiTect Primer Assay	Qiagen	Cat# QT00094234
Hs_RRAGC_1_SG QuantiTect Primer Assay	Qiagen	Cat# QT00086527
Hs_WIPI-2_va.1_SG QuantiTect Primer Assay	Qiagen	Cat# QT01018234
Hs_GABARAPL1_1_SG QuantiTect Primer Assay	Qiagen	Cat# QT00096509
Hs_ATG7_2_SG QuantiTect Primer Assay	Qiagen	Cat# QT01879556

List of primers and siRNA probes used in this study
