# Supplementary material for: Differential contribution of TFE3 isoforms to cell motility and invasion
Source: EMBO Rep. 2025 Dec 8;27(2):471–500. doi: 10.1038/s44319-025-00659-3 (PMC12852735; doi:10.1038/s44319-025-00659-3)
Supplement: Supplementary file 2 — Appendix [file 44319_2025_659_MOESM2_ESM.pdf]

APPENDIX FIGURES

Differential contribution of TFE3 isoforms to cell motility and invasion

Pablo S Contreras[1#], José A Martina[1#], Katie Rollins[1], Eutteum Jeong[1], Alberto Rissone[1], Rosa Puertollano[1*]

Appendix Figure S1. Differential expression of TFE3 isoforms in HeLa cells in response to stress. Page 2.

Appendix Figure S2. Differential expression of TFE3 isoforms in response to refed conditions in the presence of Torin-1. Page 3.

Appendix Figure S3. Nuclear and cytoplasmic fractionation assay in ARPE19 cells under different stress conditions. Page 4.

Appendix Figure S4. Differential expression of TFE3 isoforms in response to different type of stressors. Pages 5-6.

Appendix Figure S5. TFE3-L and TFE3-S interacts with active Rag heterodimers. Page 7.

Appendix Figure S6. TFE3-L induced motility in HeLa cells. Page 8.

Appendix Figure S7. TFE3L induced invasiveness is not autophagy dependent. Pages 9-10.

Appendix Figure S8. TFE3-L transcriptional activity drives migratory behaviors. Page 11.

Appendix Figure S9. Gene target selectivity between TFE3 isoforms. Page 12.

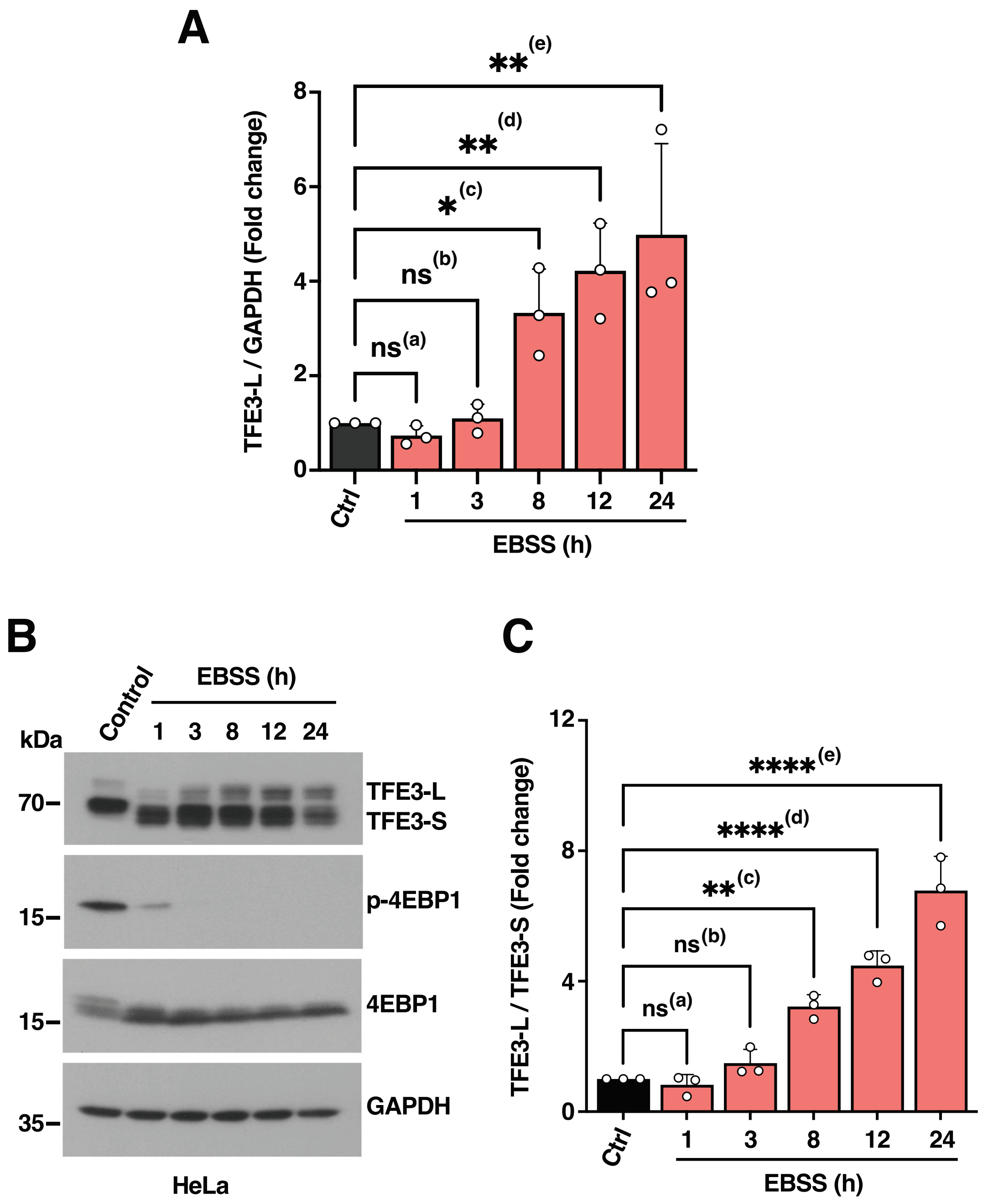

Appendix Figure S1. Differential expression of TFE3 isoforms in HeLa cells in response to stress. (A) Quantification of protein levels showing TFE3-L/GAPDH ratio expressed as fold change as shown in **(Figure 1A).** Data are presented as mean ± SD of three independent experiments. (ns) not significant (a)*P*=0.9968; (ns) not significant (b)*P*>0.9999; *(c)*P*=0.0483; **(d)*P*=0.0067; **(e)*P*=0.0013 (one-way ANOVA followed by Dunnett's multiple comparison post-test). (B) Immunoblot analysis of protein lysates from HeLa cells treated with EBSS for 1, 3, 8, 12, and 24h. (**C)** Quantification of protein levels showing TFE3-L/TFE3-S ratio expressed as fold change as shown in (B). Data are presented as mean ± SD of three independent experiments. (ns) not significant (a)*P*=0.9984; (ns) not significant (b)*P*=0.8665; **(c)*P*=0.0028; ****(d)*P*<0.0001; ****(e)*P*<0.0001 (one-way ANOVA followed by Tukey's multiple comparison post-test).

Appendix Figure S1

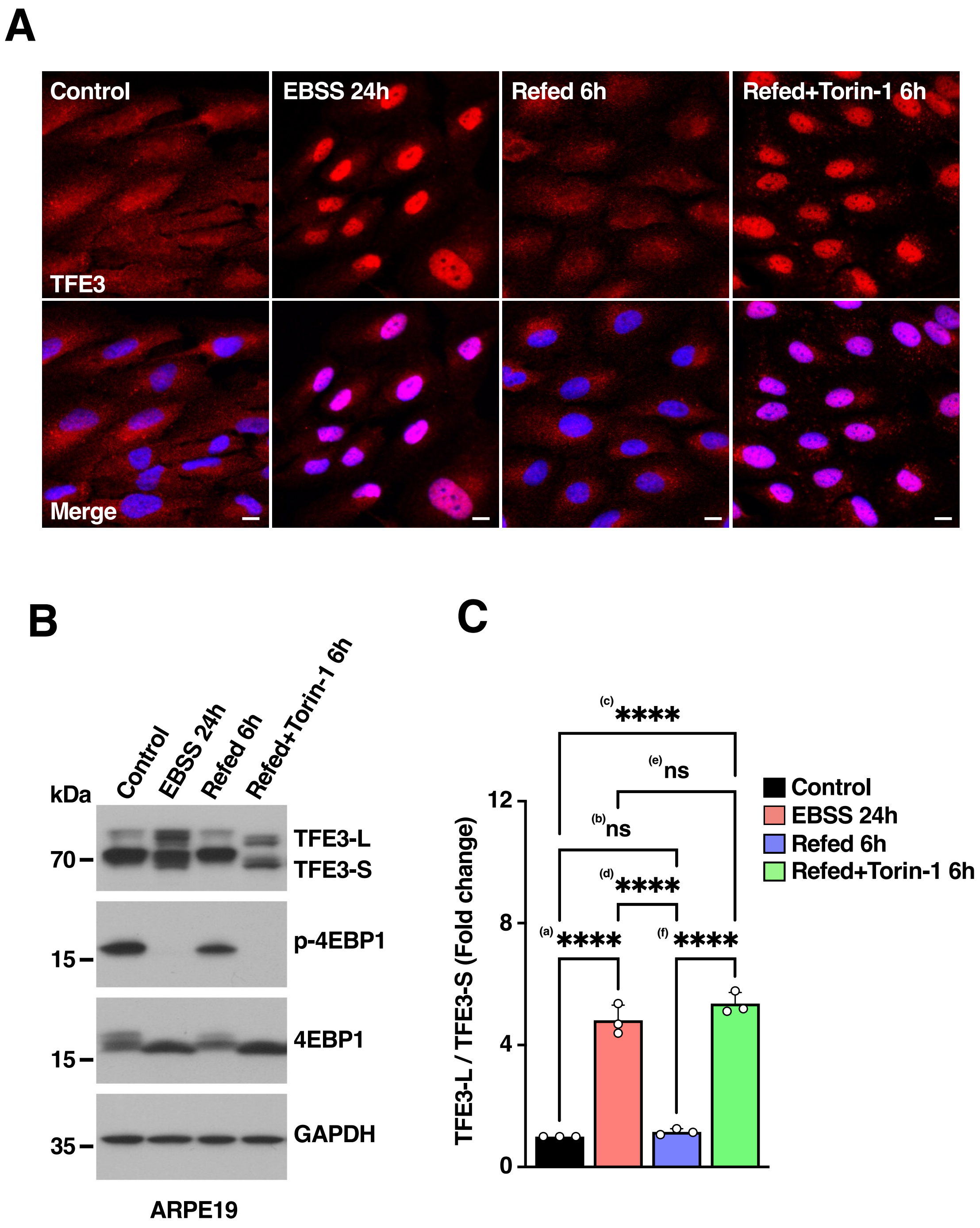

Appendix Figure S2. Differential expression of TFE3 isoforms in response to refed conditions in the presence of Torin-1. (A) Immunofluorescence confocal microscopy of ARPE19 cells showing the subcellular distribution of recombinants TFE3 (red) in response to treatment with EBSS for 24h and after refed with complete media in the absence or presence of 250 nM Torin-1 for 6h. DNA in the nucleus is stained with DAPI (blue). Scale bars: 10 µm. **(B)** Immunoblot analysis of protein lysates from ARPE19 cells treated with EBSS for 24h followed by incubation in complete media (Refed) in the absence or presence of 250 nM Torin-1 for 6h. **(C)** Quantification of protein levels showing TFE3-L/TFE3-S ratio expressed as fold change as shown in (B). Data are presented as mean ± SD of three independent experiments. ****(a)P<0.0001; (ns) not significant (b)P=0.9289; ****(c)P<0.0001; ****(d)P<0.0001; (ns) not significant (e)P=0.2190; ****(f)P<0.0001 (one-way ANOVA followed by Tukey's multiple comparison post-test).

Appendix Figure S2

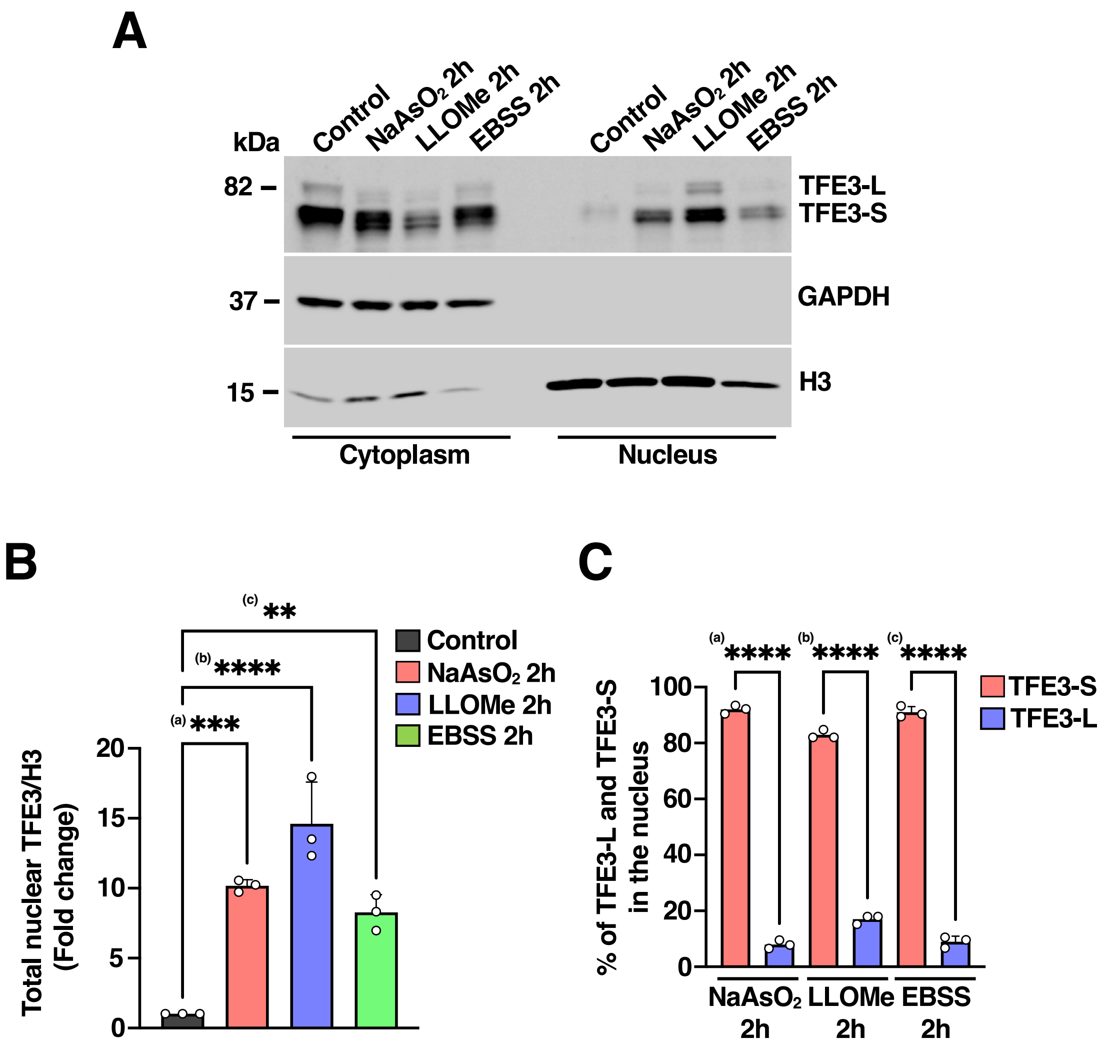

Appendix Figure S3. Nuclear and cytoplasmic fractionation assay in ARPE19 cells under different stress conditions. (A) Immunoblot analysis of nuclei/cytoplasmic fractionation assay from ARPE19 cells treated with 250 µM $NaAsO_2$, 1mM LLOMe, and EBSS (starvation media) for 2h. **(B)** Quantification of total nuclear TFE3 in (A). Data are presented as mean ± SD of three independent experiments. $^{***(a)}$*P*=0.0003; $^{****(b)}$*P*<0.0001; $^{**(c)}$*P*<0.0016 (one-way ANOVA followed by Tukey's multiple comparison post-test). **(C)** Quantification of TFE3-L and TFE3-S compared to total TFE3 in the nucleus represented as percentage. Data are presented as mean ± SD of three independent experiments. $^{****(a)}$*P*<0.0001; $^{****(b)}$*P*<0.0001; $^{****(c)}$*P*<0.0001 (one-way ANOVA followed by Tukey's multiple comparison post-test).

Appendix Figure S3

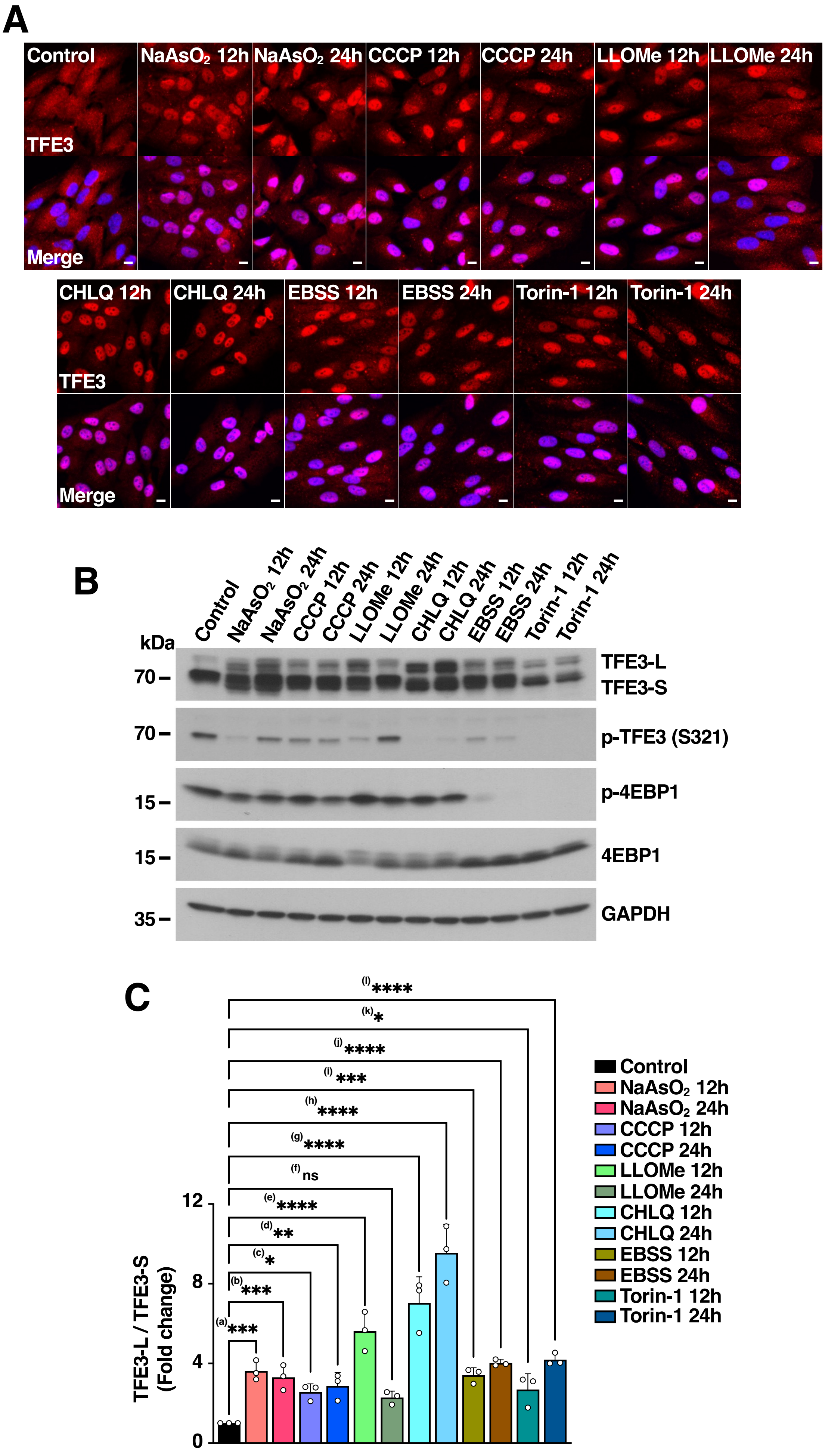

Appendix Figure S4

Appendix Figure S4. Differential expression of TFE3 isoforms in response to different type of stressors. **(A)** Immunofluorescence confocal microscopy of ARPE19 cells showing the subcellular distribution of TFE3 (red) in response to treatments with either 50 μM $NaAsO_2$, 10 μM CCCP, 1 mM LLOMe, 50 μM Chloroquine (CHLO), EBSS or 250 nM Torin-1 for 12h and 24h. DNA in the nucleus is stained with DAPI (blue). Scale bars: 10 μm. **(B)** Immunoblot analysis of protein lysates from ARPE19 cells treated with either 50 μM $NaAsO_2$, 10 μM CCCP, 1 mM LLOMe, 50 μM Chloroquine (CHLO), EBSS, or 250 nM Torin-1 for 12 and 24h. **(C)** Quantification of protein levels showing TFE3-L/TFE3-S ratio expressed as fold change as shown in (B). Data are presented as mean ± SD of three independent experiments. $^{***(a)}$*P*=0.0001; $^{***(b)}$*P*=0.0007; $^{*(c)}$*P*=0.0383; $^{**(d)}$*P*=0.0064; $^{****(e)}$*P*<0.0001; (ns) not significant $^{(f)}$*P*=0.0995; $^{****(g)}$*P*<0.0001; $^{****(h)}$*P*<0.0001; $^{***(i)}$*P*=0.0004; $^{****(j)}$*P*<0.0001; $^{*(k)}$*P*=0.0160; $^{****(l)}$*P*<0.0001 (one-way ANOVA followed by Dunnett's multiple comparison post-test

Appendix Figure S4

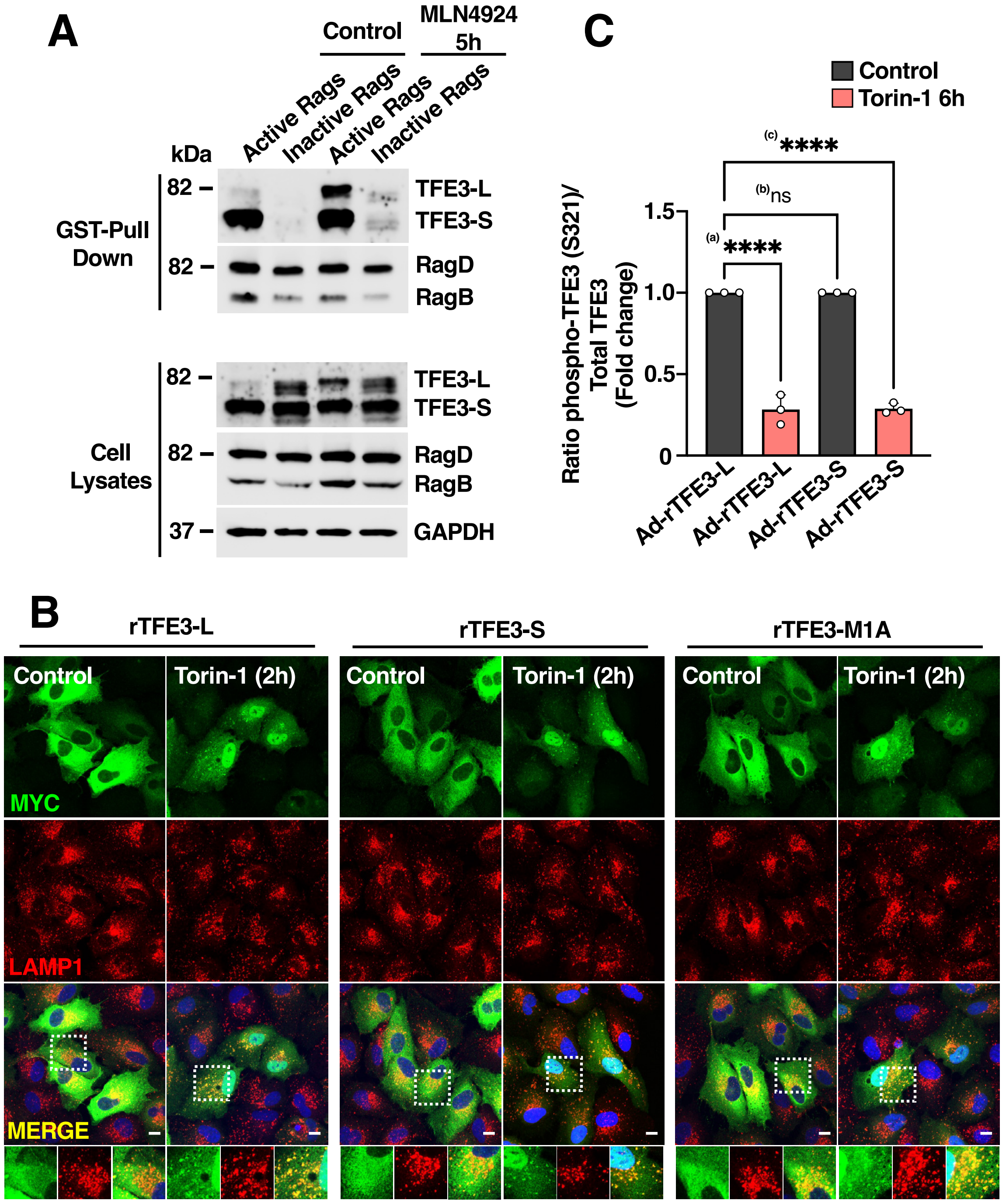

Appendix Figure S5. TFE3-L and TFE3-S interacts with active Rag heterodimers. (A) Endogenous TFE3 affinity-purified with glutathione S-transferase (GST) fused to the C terminus of active Rags heterodimers (RagB GTP/RagD GDP) in ARPE19 cells treated with 1µM MLN4924 for 5h. Data is representative of three independent experiments. **(B)** Representative immunofluorescence confocal microscopy images of ARPE19 cells transfected with rTFE3-L, rTFE3-S or rTFE3-M1A and treated with 0.3 µM Torin-1 for 2h. Insets show a 2-fold magnification of the indicated region. Scale bar: 10 µm. **(C)** Quantification of phospho-TFE3 (Serine 321) protein levels expressed as fold change in ARPE19 cells treated with 0.3 µM Torin-1 for 6h. Data are presented as mean ± SD of three independent experiments. ****(a)$P<0.0001$; (ns) not significant (b)$P>0.9999$; ****(c)$P<0.0001$ (one-way ANOVA followed by Dunnett's multiple comparison post-test).

Appendix Figure S5

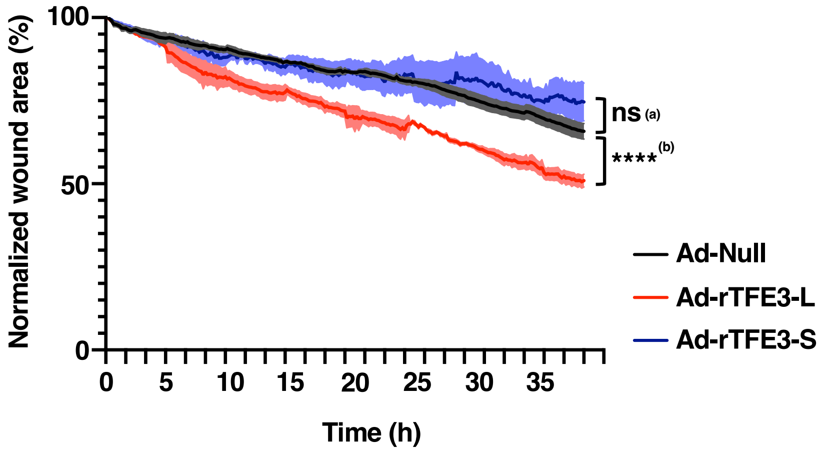

Appendix Figure S6. TFE3-L induced motility in HeLa cells. Quantification of scratch wound assay experiment using HeLa cells infected with Adenovirus expressing rTFE3-S or rTFE3-L and control virus (Ad-Null). Data are presented as mean ± SD of three independent experiments. (ns) not significant [(a)]P=0.2842; ****[(b)]P<0.0001 (one-way ANOVA followed by Tukey's multiple comparison post-test).

Appendix Figure S6

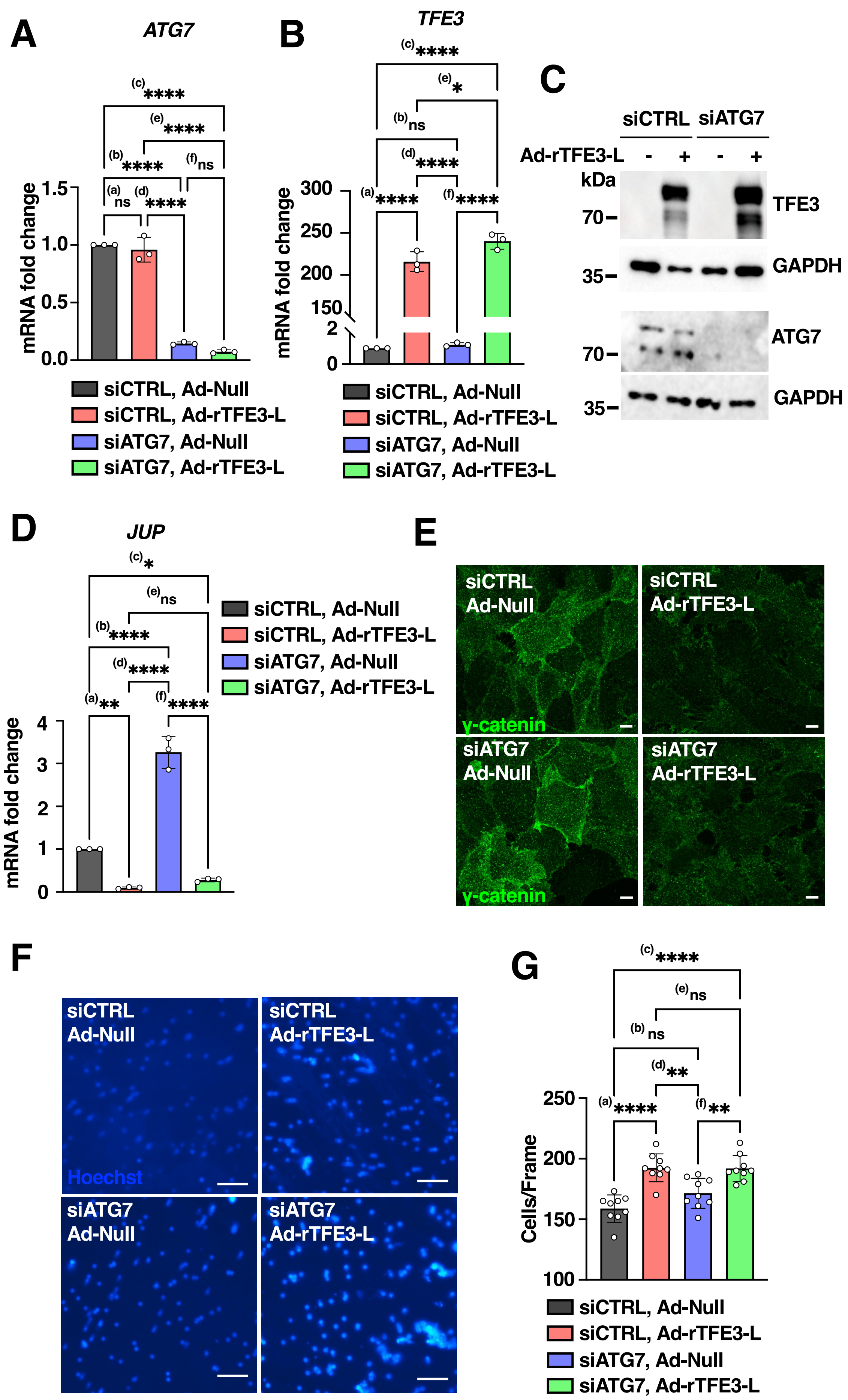

Appendix Figure S7

Appendix Figure S7. TFE3L induced invasiveness is not autophagy dependent. (A) Relative quantitative RT-PCR analysis of mRNA expression of ATG7 in ARPE19 cells treated with siRNA targeting ATG7 or non-targeting control and infected with either adenovirus expressing rTFE3-L-Myc or Control for 30h. Data are presented as mean ± SD of three independent experiments. (ns) not significant $^{(a)}P$=0.788; $^{****(b)}P$<0.0001; $^{****(c)}P$<0.0001; $^{****(d)}P$<0.0001; $^{****(e)}P$<0.0001; (ns) not significant $^{(f)}P$=0.4283 (two-way ANOVA followed by Tukey's multiple comparison post-test). **(B)** Relative quantitative RT-PCR analysis of mRNA expression of TFE3 in ARPE19 cells treated as described in (A). Data are presented as mean ± SD of three independent experiments. $^{****(a)}P$<0.0001; (ns) not significant $^{(b)}P$>0.9999; $^{****(c)}P$<0.0001; $^{****(d)}P$<0.0001; $^{*(e)}P$=0.0133; $^{****(f)}P$<0.0001 (two-way ANOVA followed by Tukey's multiple comparison post-test). **(C)** Immunoblot analysis of protein lysates from ARPE19 cells treated as described in (A). **(D)** Relative quantitative RT-PCR analysis of mRNA expression of JUP in ARPE19 cells treated as described in (A). Data are presented as mean ± SD of three independent experiments. $^{**(a)}P$=0.0036; $^{****(b)}P$<0.0001; $^{*(c)}P$=0.0115; $^{****(d)}P$<0.0001; (ns) not significant $^{(e)}P$=0.6203; $^{****(f)}P$<0.0001 (two-way ANOVA followed by Tukey's multiple comparison post-test). **(E)** Immunofluorescence using laser scanning confocal microscopy showing γ-catenin organization in ARPE19 cells treated as described in (A). Scale bar: 10 µm. **(F)** Representative images of Hoechst-stained membranes from Boyden chamber invasion assay after removal of non-invading ARPE19 cells treated as described in (A). Scale bar: 50 µm. **(G)** Quantification of (F) by nuclear count per frame (540µmx400µm). Data are presented as mean ± SD of three independent experiments. $^{****(a)}P$<0.0001; (ns) not significant $^{(b)}P$=0.1099; $^{****(c)}P$<0.0001; $^{**(d)}P$=0.0033; (ns) not significant $^{(e)}P$=0.9996; $^{**(f)}P$=0.0042 (two-way ANOVA followed by Tukey's multiple comparison post-test).

Appendix Figure S7

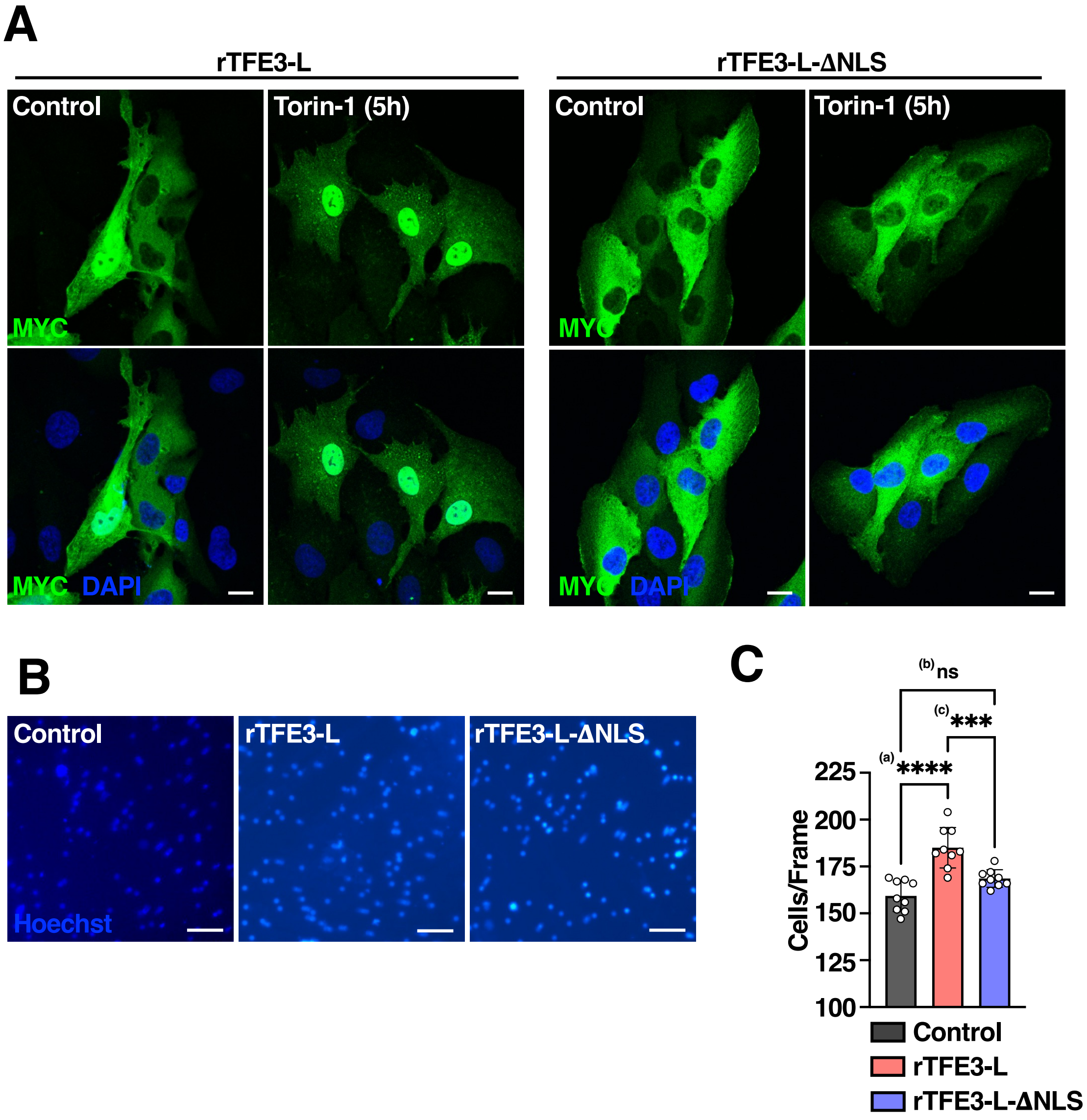

Appendix Figure S8. TFE3-L transcriptional activity drives migratory behaviors. (A) Immunofluorescence by laser scanning confocal microscopy of ARPE19 cells overexpressing r-TFE3-L-Myc or rTFE3-L-**Δ**NLS-Myc for 24h condition that results in increased nuclear accumulation of recombinant TFE3. Then cells were treated with 0.3 µM Torin-1 or vehicle (control) for 5h to induce nuclear localization showing TFE3 distributions by Myc fluorescence. Scale bar: 10 µm. **(B)** Representative images of Hoechst-stained membranes from Boyden chamber invasion assay after removal of non-invading ARPE19 cells expressing either rTFE3-L-Myc or rTFE3-L-**Δ**NLS-Myc. Scale bar: 50 µm. **(C)** Quantification of (B) by nuclear count per frame (540µmx400µm). Data are presented as mean ± SD of three independent experiments. ****(a)*P*<0.0001; (ns) not significant (b)*P*=0.0681; ***(c)*P*=0.0009 (one-way ANOVA followed by Tukey's multiple comparison post-test).

Appendix Figure S8

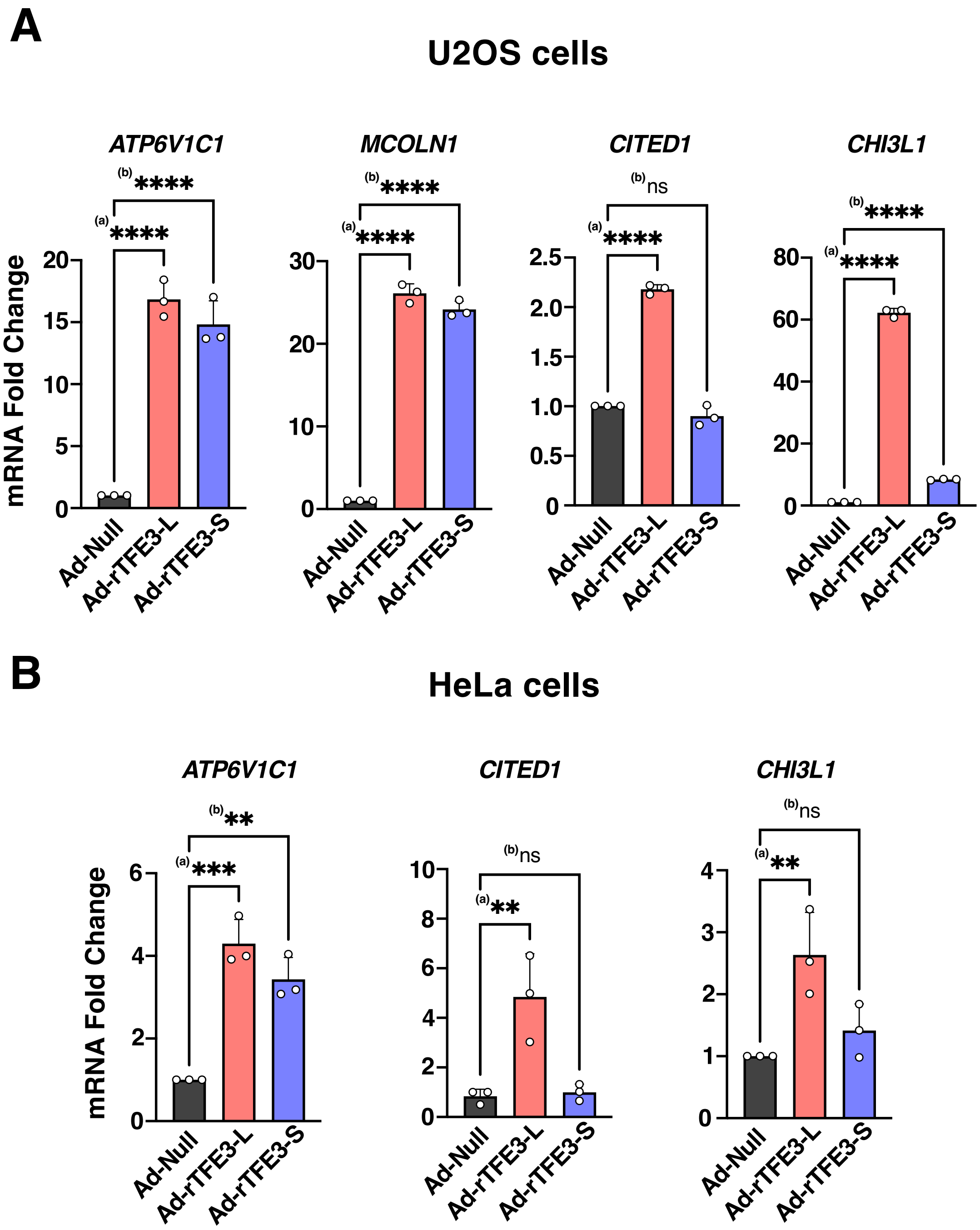

Appendix Figure S9. Gene target selectivity between TFE3 isoforms. Relative quantitative PCR analysis of the expression of different genes in U20S cells **(A)** and in Hela cells **(B)** infected with control adenovirus (Null) or adenovirus expressing recombinant TFE3-L and rTFE3-S for 30h. Data in (A) are presented as mean ± SD of three independent experiments. ATP6V1C1 ($^{***(a)}P$<0.0001; $^{***(b)}P$<0.0001), MCOLN1 ($^{***(a)}P$<0.0001; $^{***(b)}P$<0.0001), CITED1 ($^{***(a)}P$<0.0001; (ns) not significant $^{(b)}P$=0.1876), CHI3L1 ($^{***(a)}P$<0.0001; $^{***(b)}P$<0.0001) (one-way ANOVA followed by Dunnett's multiple comparison post-test). Data in (B) are presented as mean ± SD of three independent experiments. ATP6V1C1 ($^{***(a)}P$=0.0002; $^{**(b)}P$=0.0011), CITED1 ($^{**(a)}P$=0.0058; (ns) not significant $^{(b)}P$=0.9733), CHI3L1 ($^{**(a)}P$=0.0093; (ns) not significant $^{(b)}P$=0.493) (one-way ANOVA followed by Dunnett's multiple comparison post-test).

Appendix Figure S9
