# Supplementary material for: Differential contribution of TFE3 isoforms to cell motility and invasion
Source: EMBO Rep. 2025 Dec 8;27(2):471–500. doi: 10.1038/s44319-025-00659-3 (PMC12852735; doi:10.1038/s44319-025-00659-3)

Source Data Fig. 1E

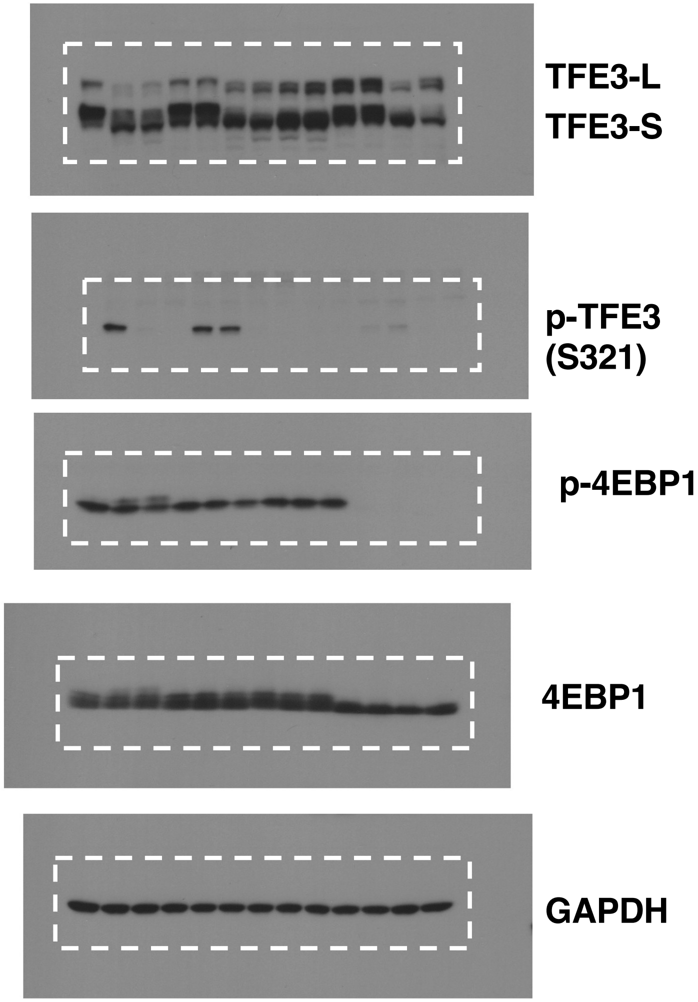

Supplement: Supplementary file 9 — Source data Fig. 1 [file 44319_2025_659_MOESM9_ESM.zip › Figure 1/1L/Source Data Fig 1L.pdf]
