# Supplementary figures and images for: Differential contribution of TFE3 isoforms to cell motility and invasion
Source: EMBO Rep. 2025 Dec 8;27(2):471–500. doi: 10.1038/s44319-025-00659-3 (PMC12852735; doi:10.1038/s44319-025-00659-3)

Source Data Fig. 1A

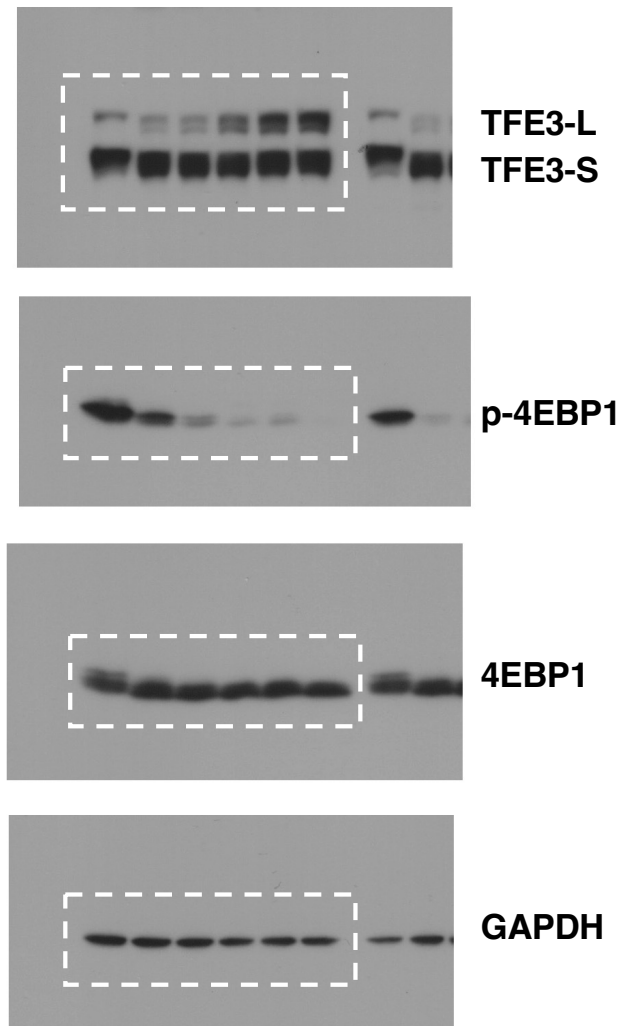

Supplement: Supplementary file 9 — Source data Fig. 1 [file 44319_2025_659_MOESM9_ESM.zip › Figure 1/1A/Source Data Fig 1A.pdf]

Source Data Fig. 1J

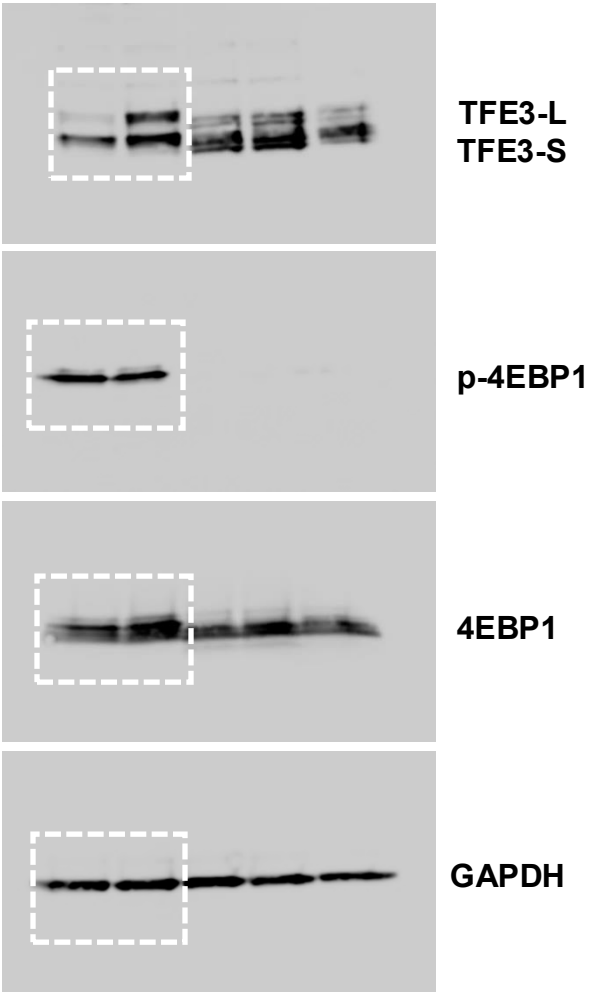

Supplement: Supplementary file 9 — Source data Fig. 1 [file 44319_2025_659_MOESM9_ESM.zip › Figure 1/1J/Source Data Fig 1J.pdf]

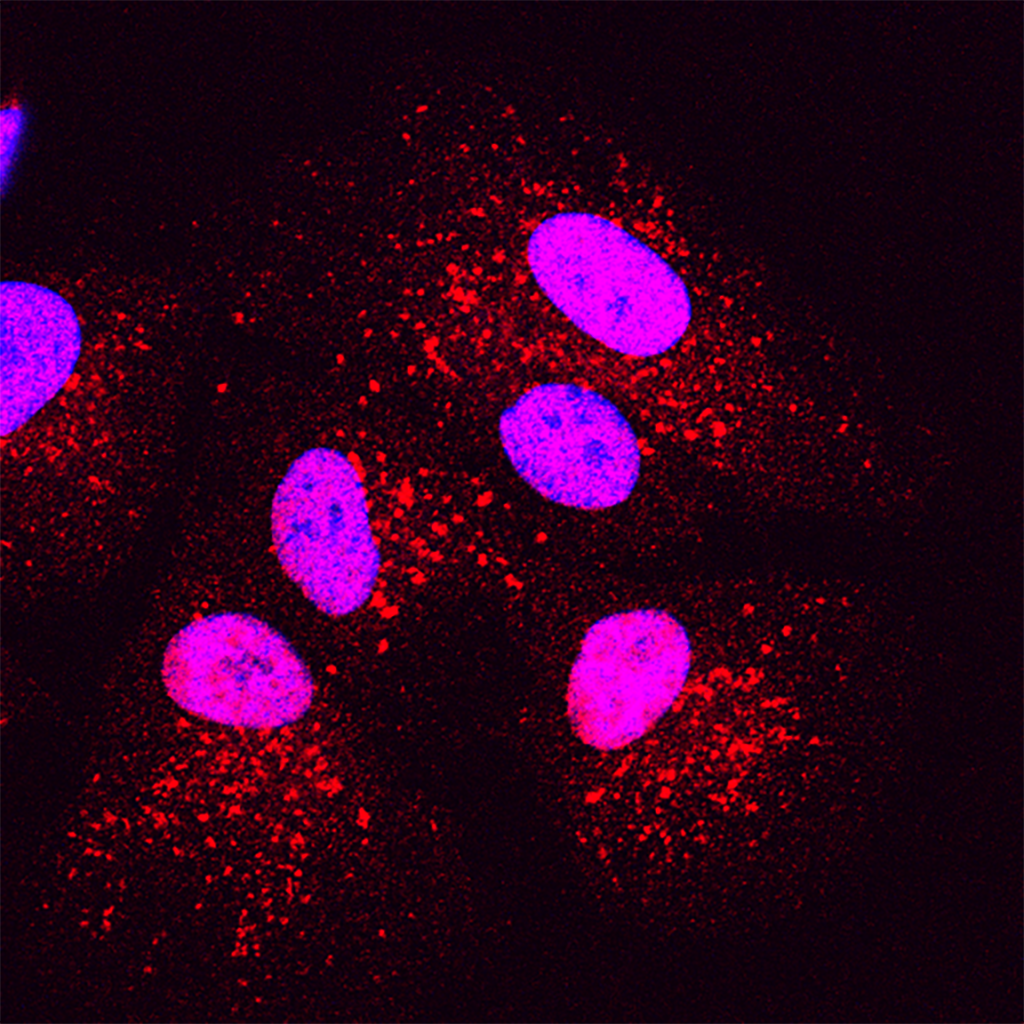

Supplement: Supplementary file 9 — Source data Fig. 1 [file 44319_2025_659_MOESM9_ESM.zip › Figure 1/1M/Torin2h.tif]

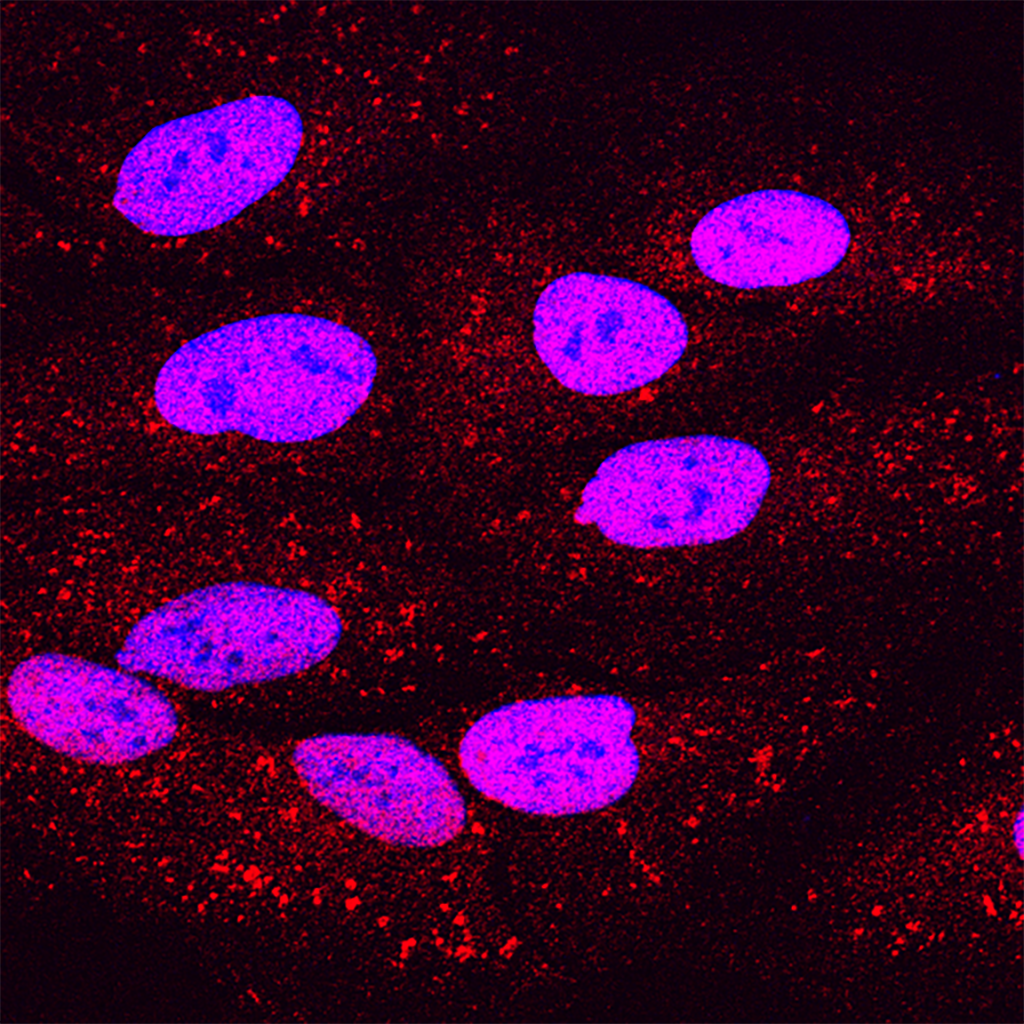

Supplement: Supplementary file 9 — Source data Fig. 1 [file 44319_2025_659_MOESM9_ESM.zip › Figure 1/1M/Torin24h.tif]

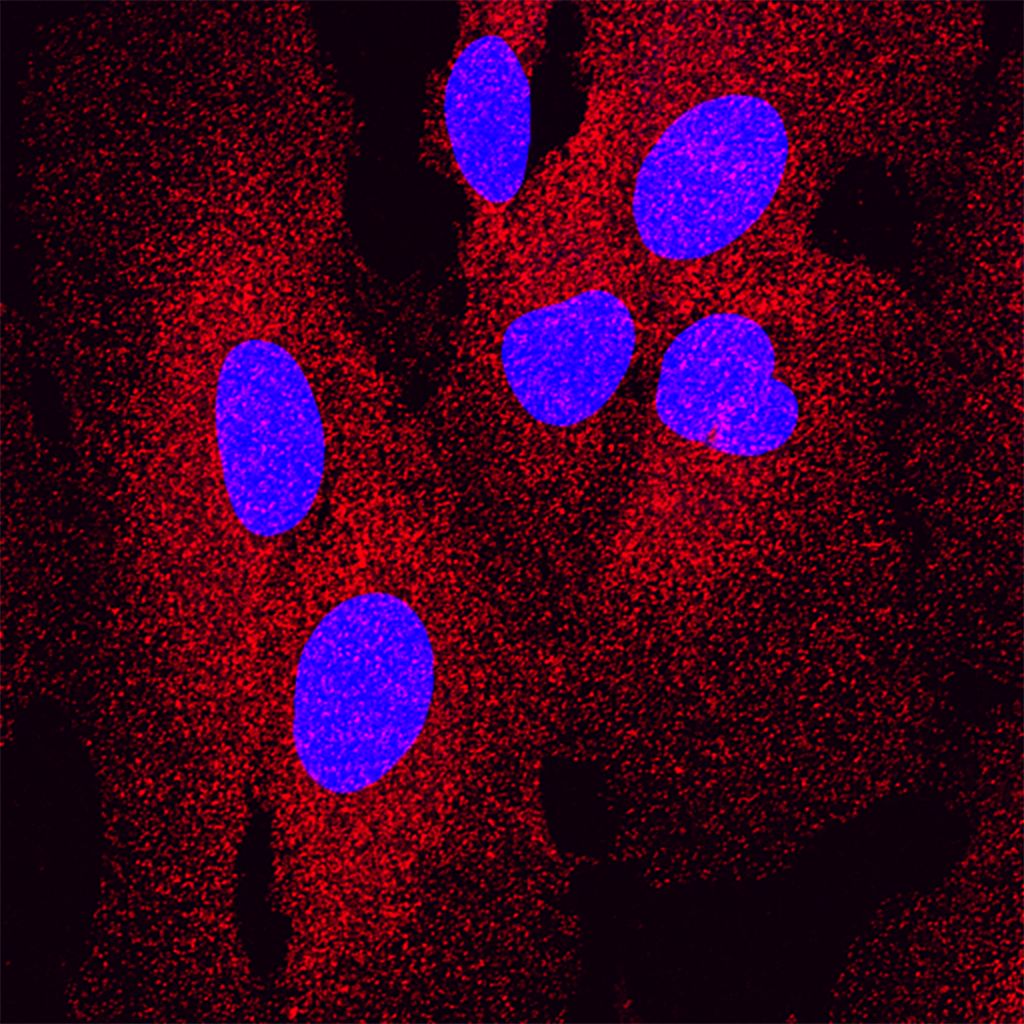

Supplement: Supplementary file 9 — Source data Fig. 1 [file 44319_2025_659_MOESM9_ESM.zip › Figure 1/1M/Control.tif]

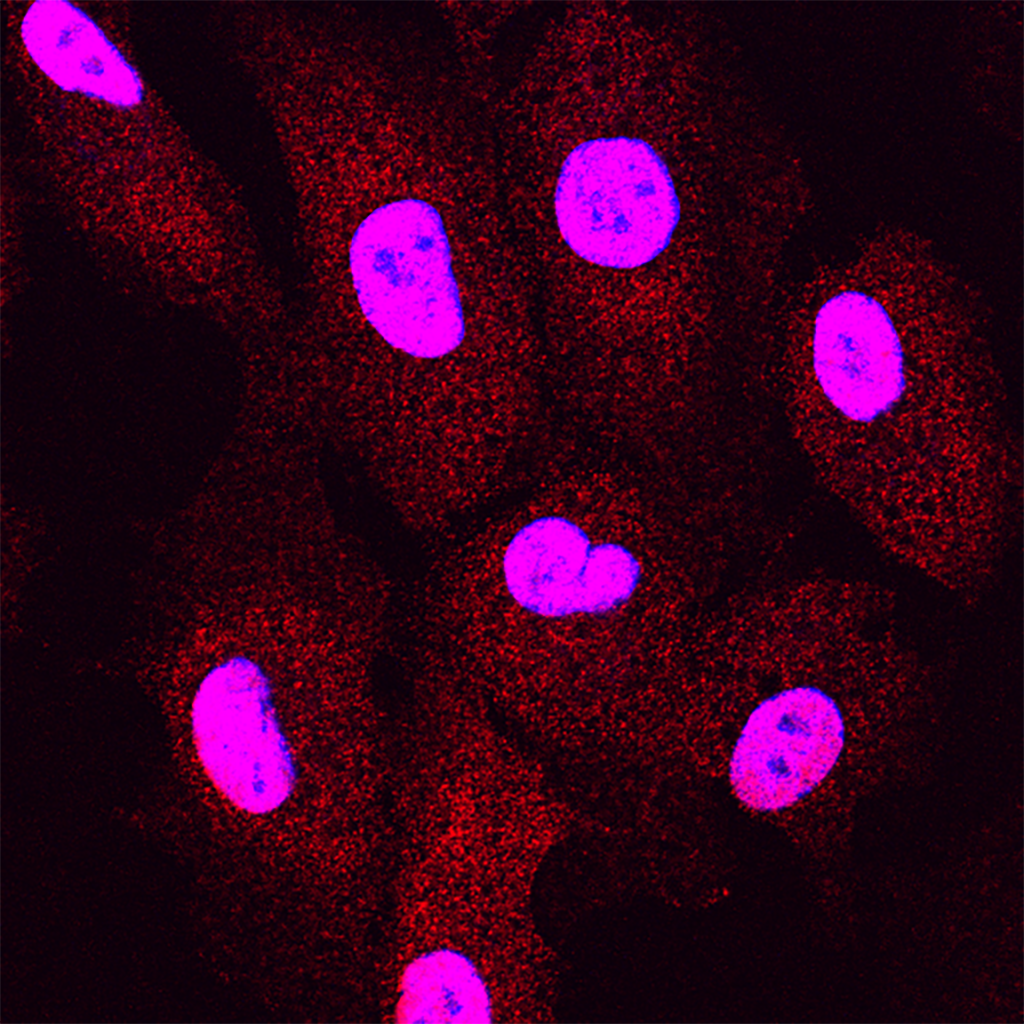

Supplement: Supplementary file 9 — Source data Fig. 1 [file 44319_2025_659_MOESM9_ESM.zip › Figure 1/1M/CHLQ4h.tif]

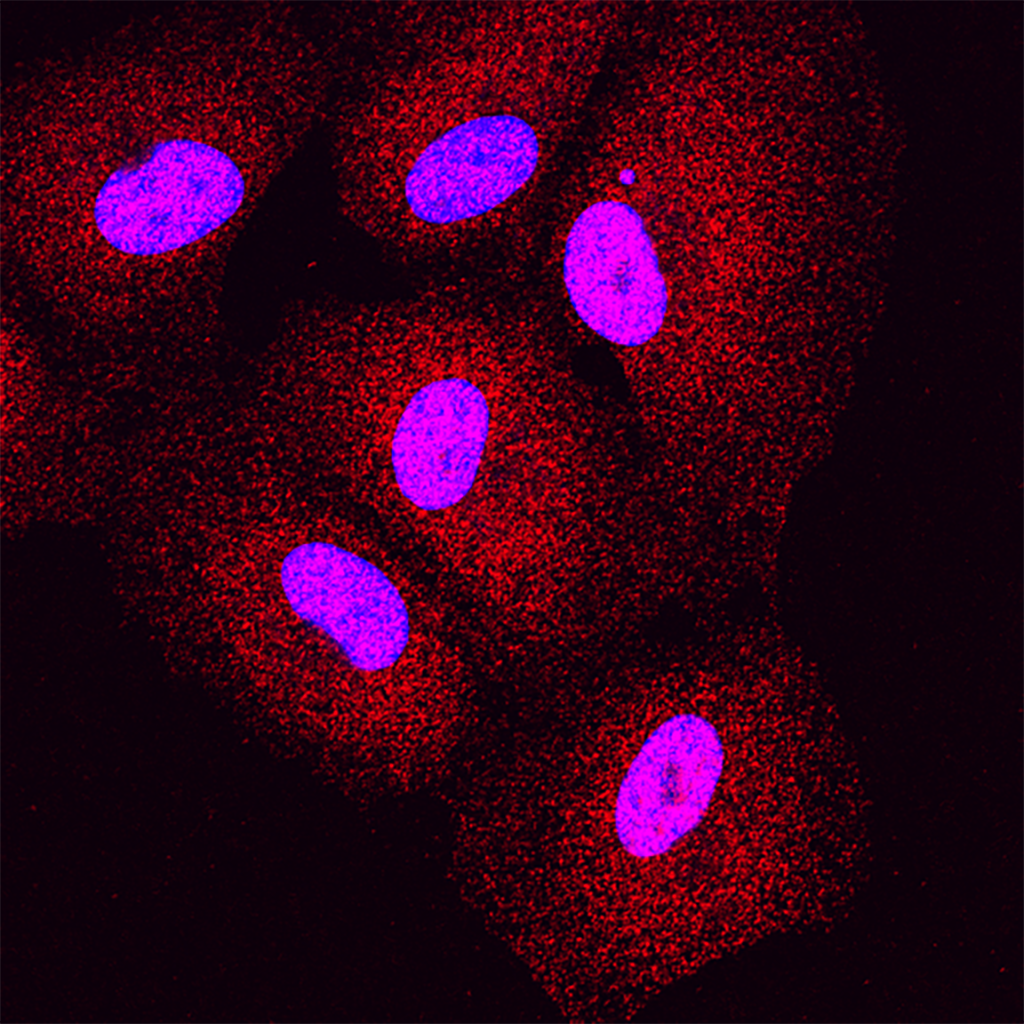

Supplement: Supplementary file 9 — Source data Fig. 1 [file 44319_2025_659_MOESM9_ESM.zip › Figure 1/1M/NaAsO2_4h.tif]

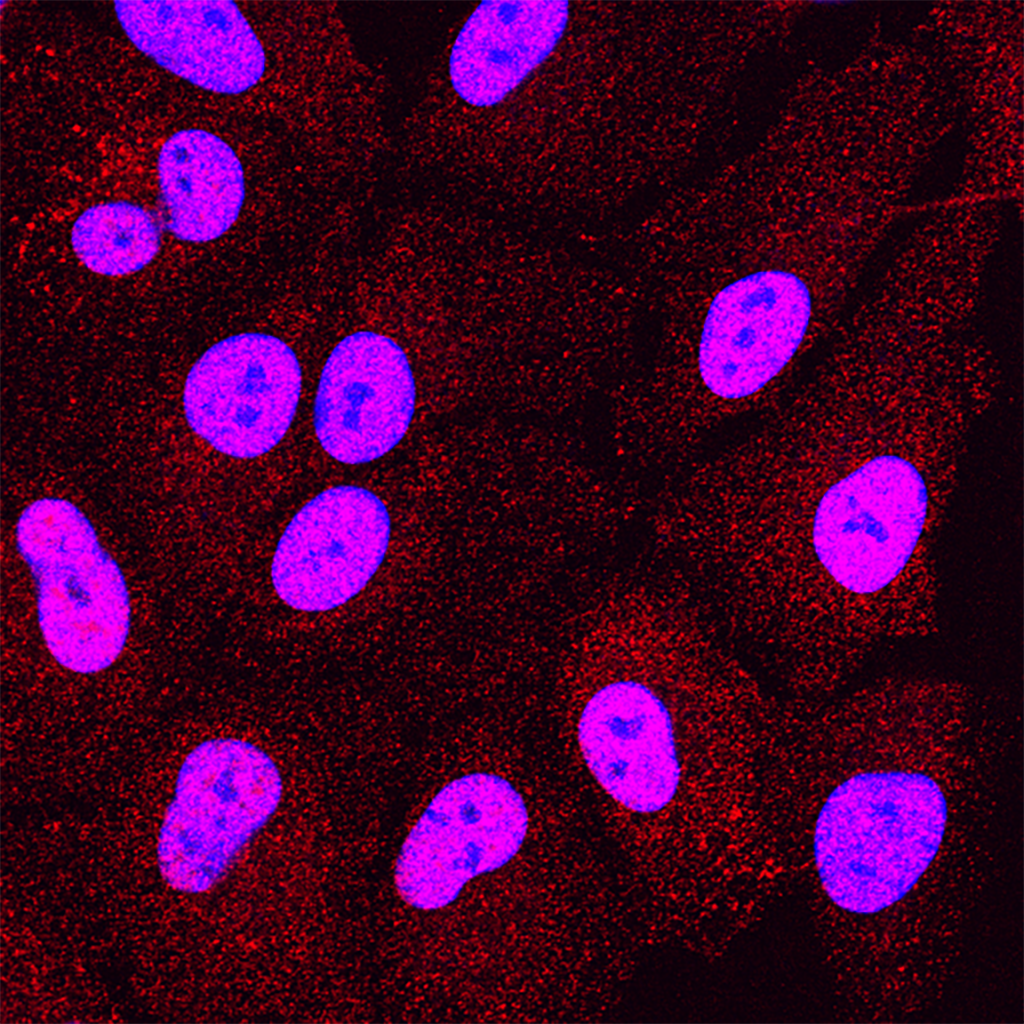

Supplement: Supplementary file 9 — Source data Fig. 1 [file 44319_2025_659_MOESM9_ESM.zip › Figure 1/1M/EBSS24h.tif]

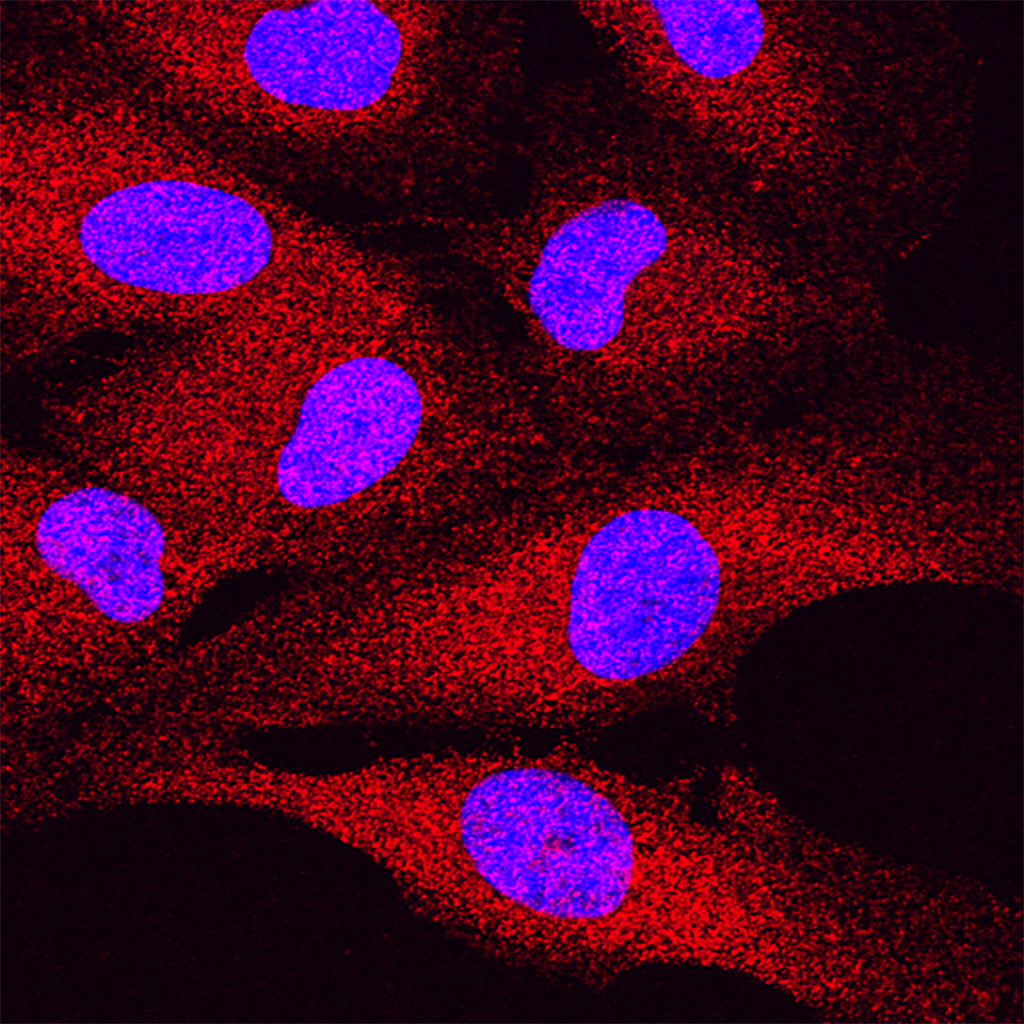

Supplement: Supplementary file 9 — Source data Fig. 1 [file 44319_2025_659_MOESM9_ESM.zip › Figure 1/1M/CCCP2h.tif]

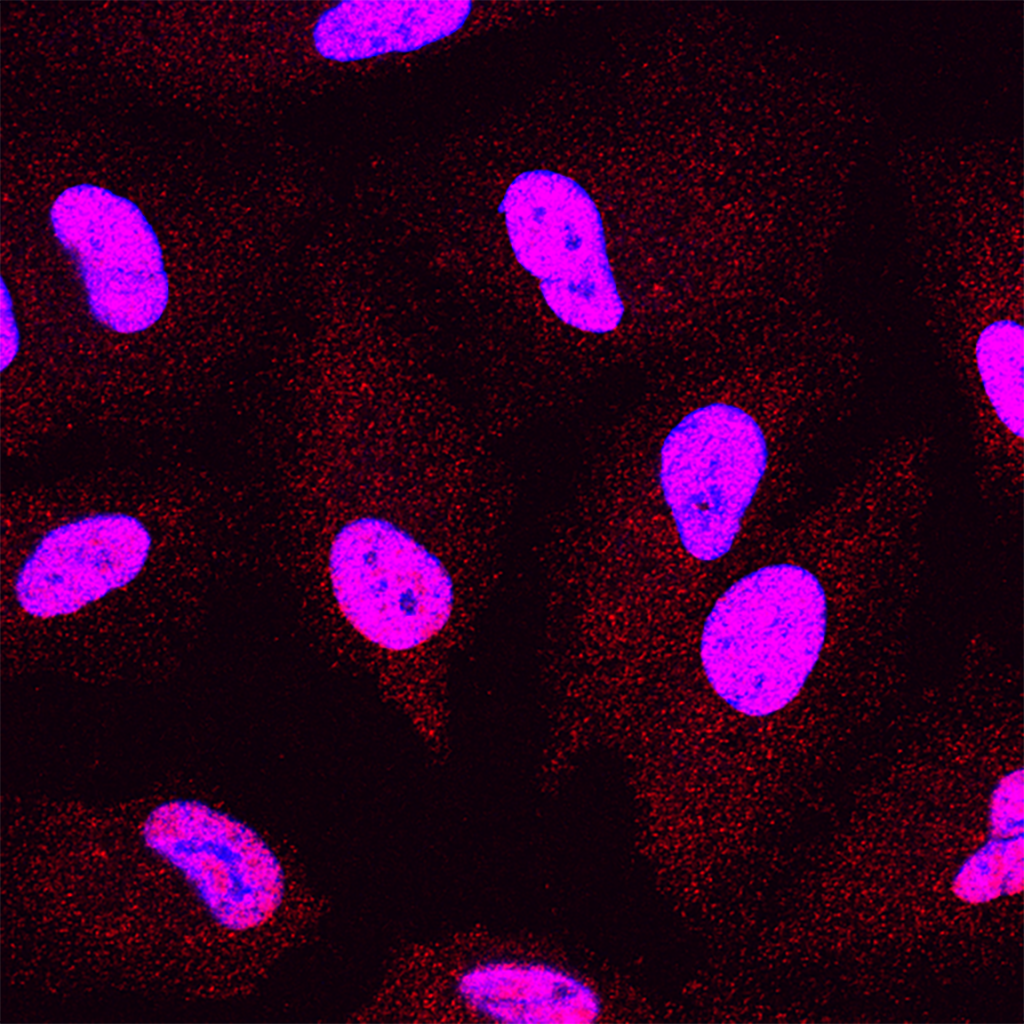

Supplement: Supplementary file 9 — Source data Fig. 1 [file 44319_2025_659_MOESM9_ESM.zip › Figure 1/1M/LLOMe4h.tif]

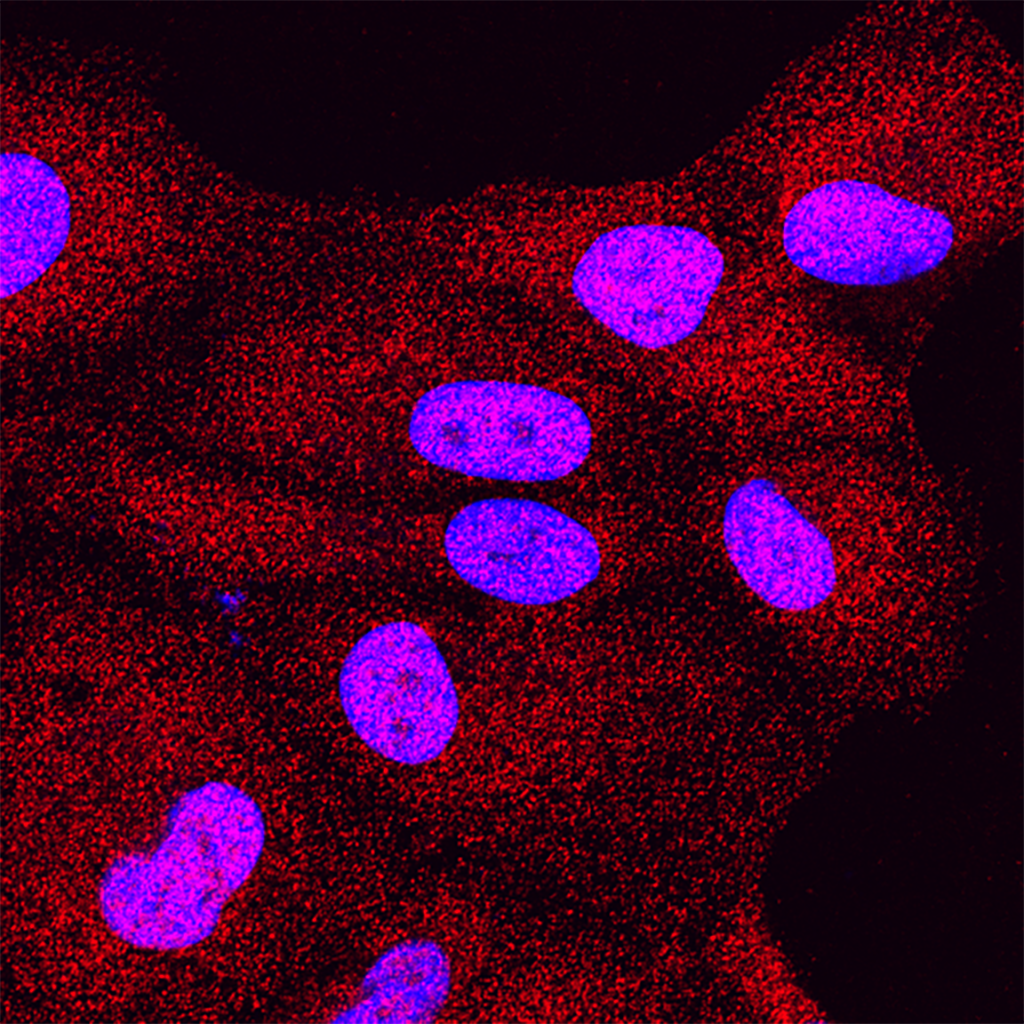

Supplement: Supplementary file 9 — Source data Fig. 1 [file 44319_2025_659_MOESM9_ESM.zip › Figure 1/1M/NaAsO2_2h.tif]

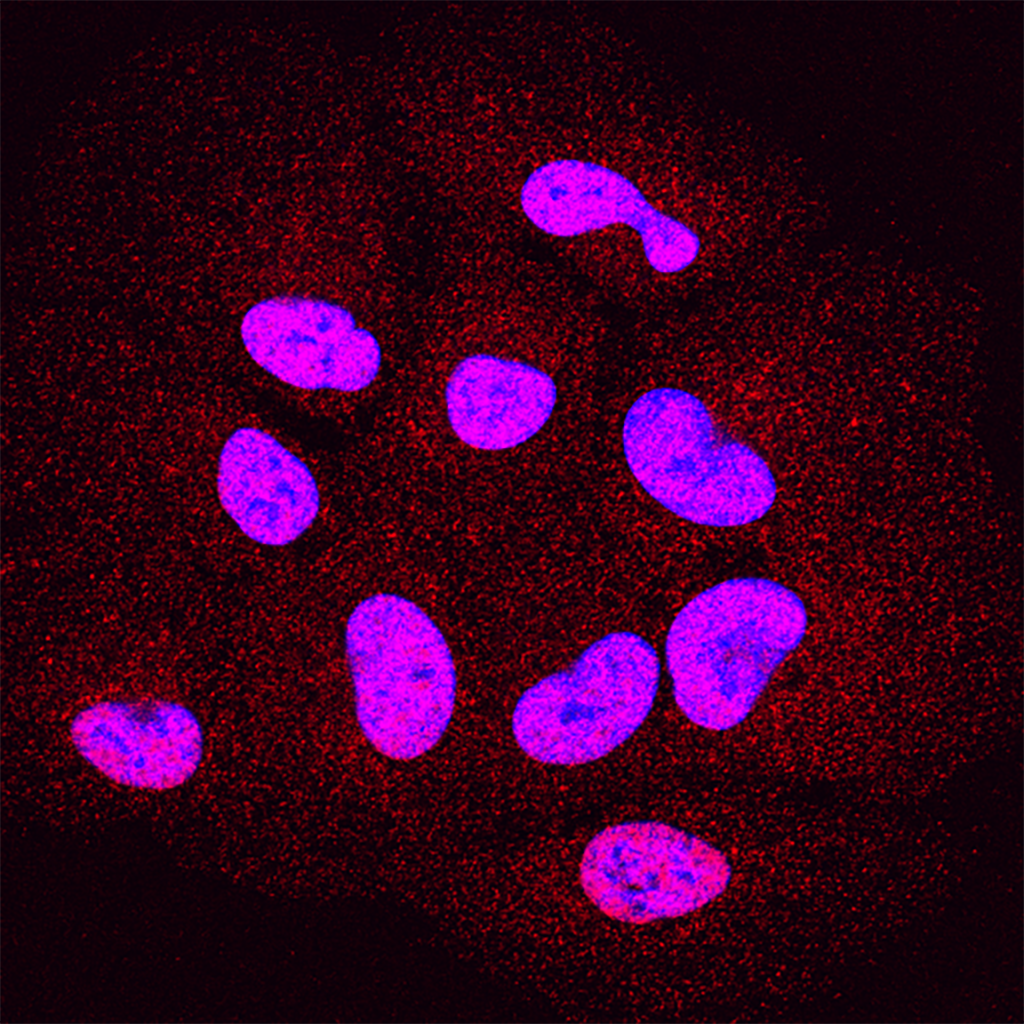

Supplement: Supplementary file 9 — Source data Fig. 1 [file 44319_2025_659_MOESM9_ESM.zip › Figure 1/1M/EBSS12h.tif]

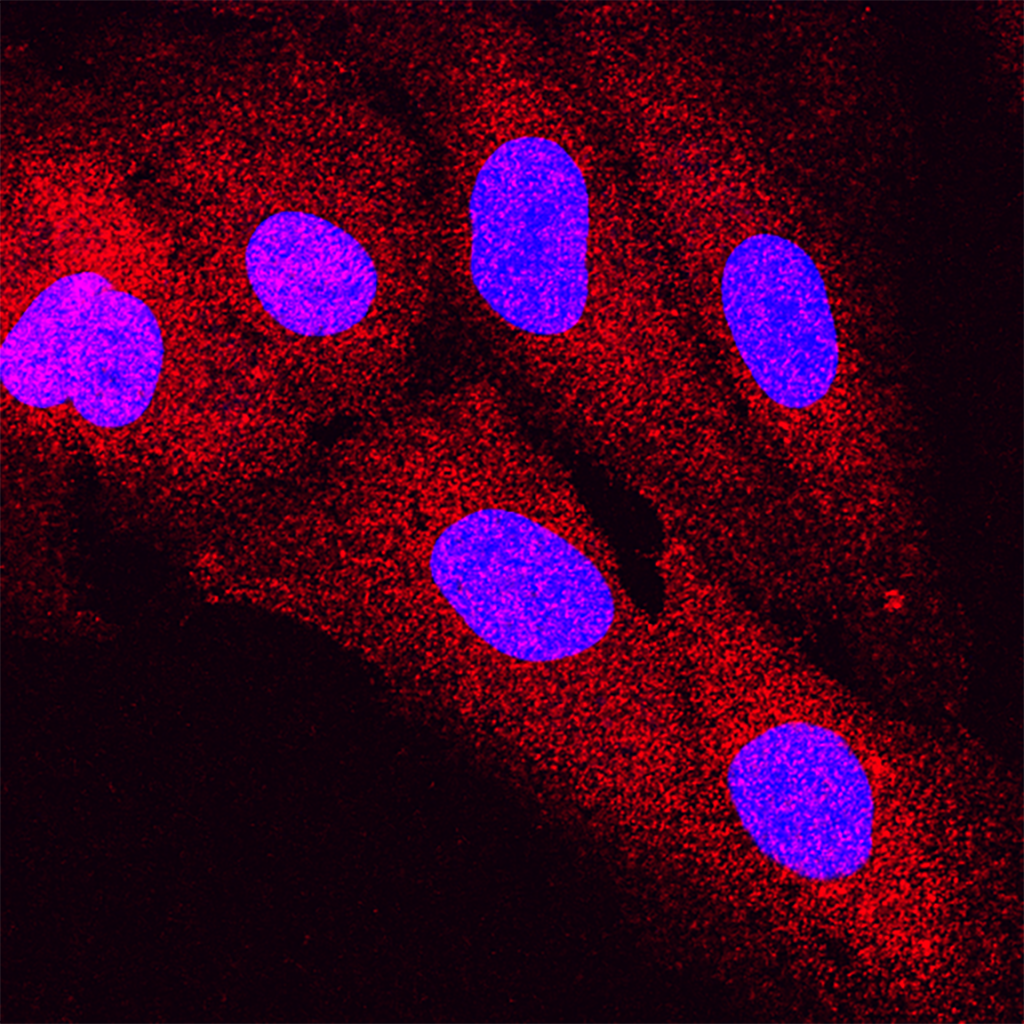

Supplement: Supplementary file 9 — Source data Fig. 1 [file 44319_2025_659_MOESM9_ESM.zip › Figure 1/1M/CCCP4h.tif]

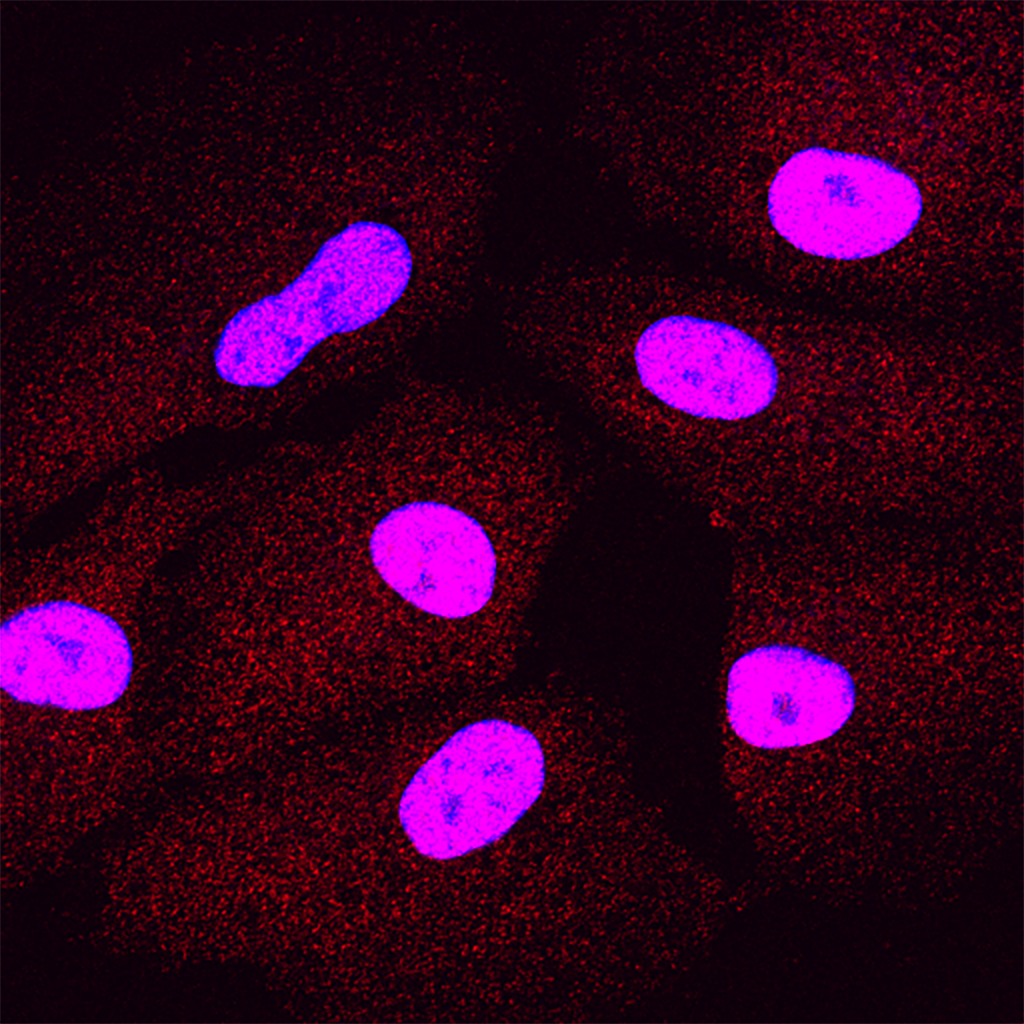

Supplement: Supplementary file 9 — Source data Fig. 1 [file 44319_2025_659_MOESM9_ESM.zip › Figure 1/1M/CHLQ2h.tif]

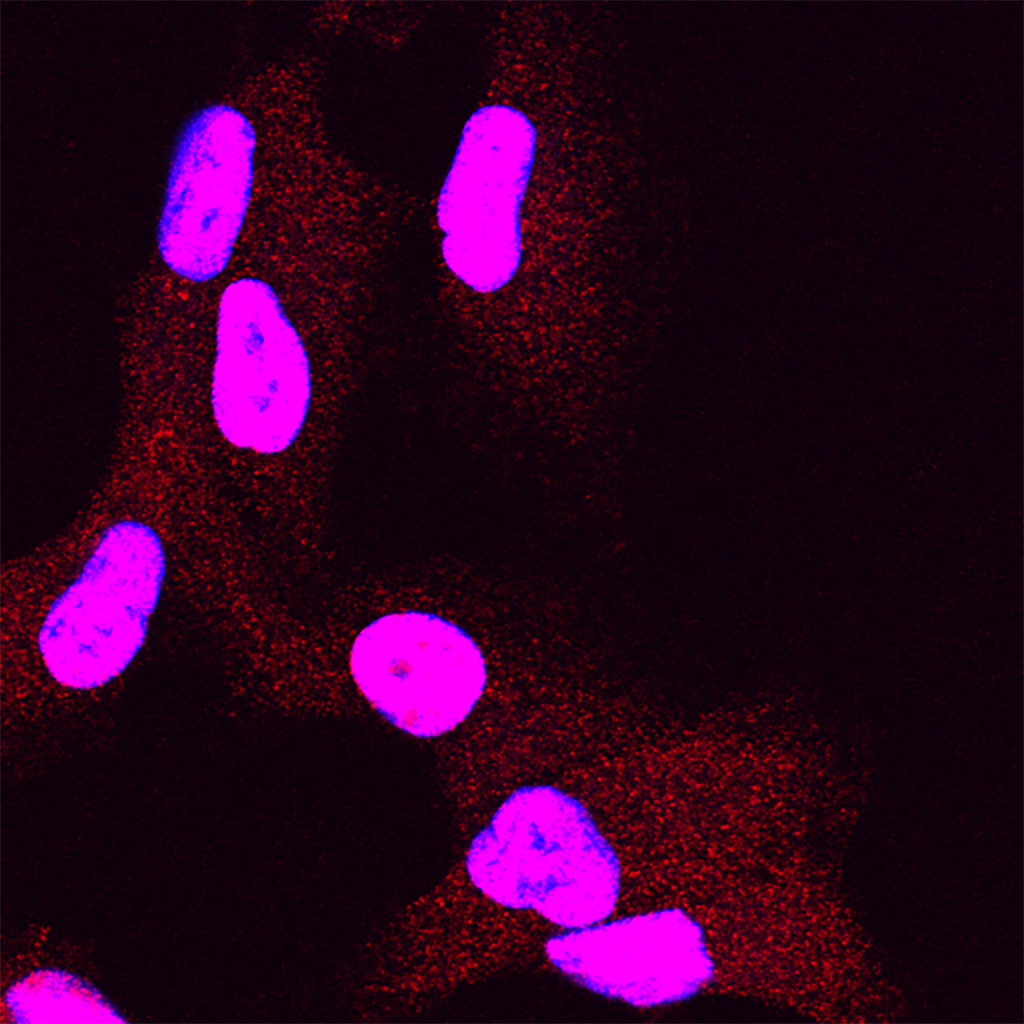

Supplement: Supplementary file 9 — Source data Fig. 1 [file 44319_2025_659_MOESM9_ESM.zip › Figure 1/1M/LLOMe2h.tif]

Source Data Fig. 1C

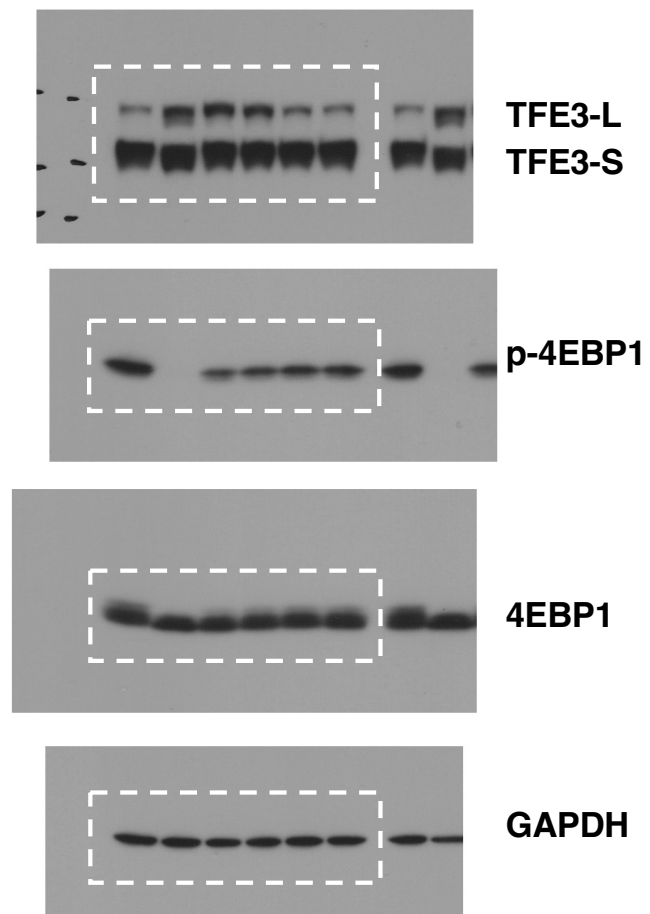

Supplement: Supplementary file 9 — Source data Fig. 1 [file 44319_2025_659_MOESM9_ESM.zip › Figure 1/1C/Source Data Fig 1C.pdf]

Source Data Fig. 1E

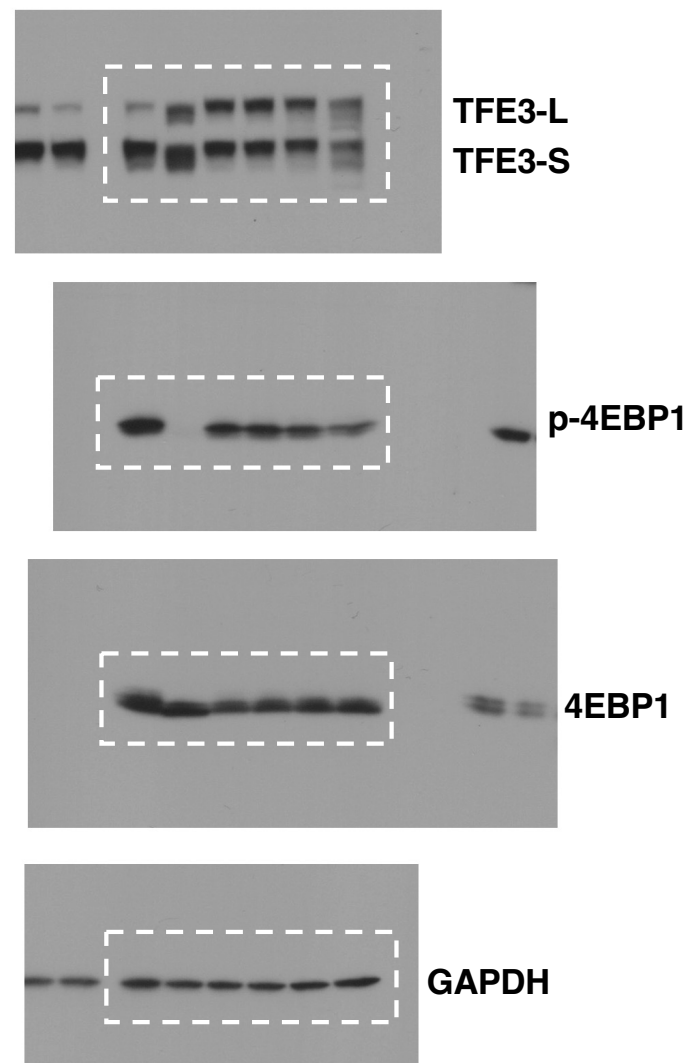

Supplement: Supplementary file 9 — Source data Fig. 1 [file 44319_2025_659_MOESM9_ESM.zip › Figure 1/1E/Source Data Fig 1E.pdf]

Source Data Fig. 1G

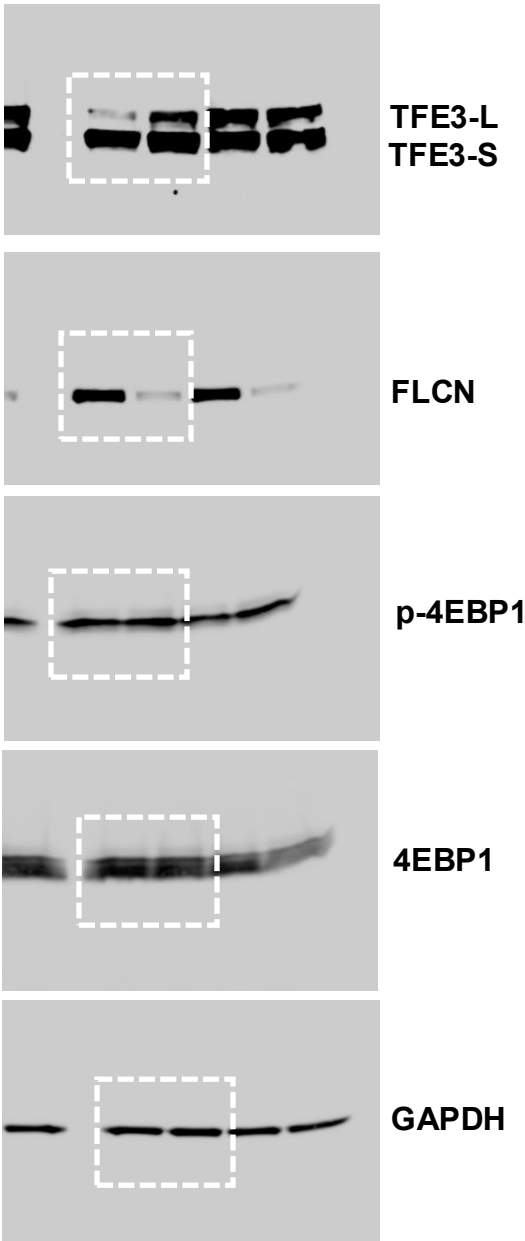

Supplement: Supplementary file 9 — Source data Fig. 1 [file 44319_2025_659_MOESM9_ESM.zip › Figure 1/1G/Source Data Fig 1G.pdf]

Source Data Fig. 2D

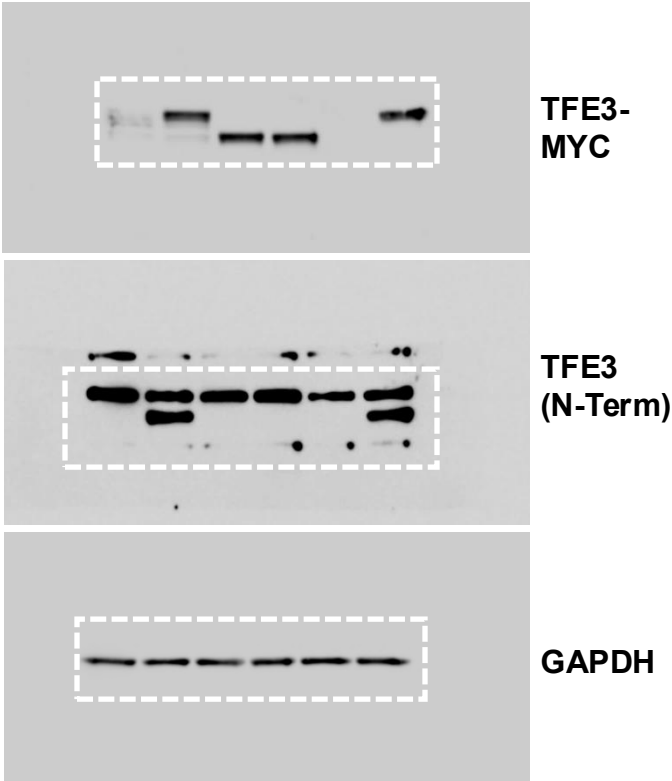

Supplement: Supplementary file 10 — Source data Fig. 2 [file 44319_2025_659_MOESM10_ESM.zip › Figure 2/2D/Source Data Fig 2D.pdf]

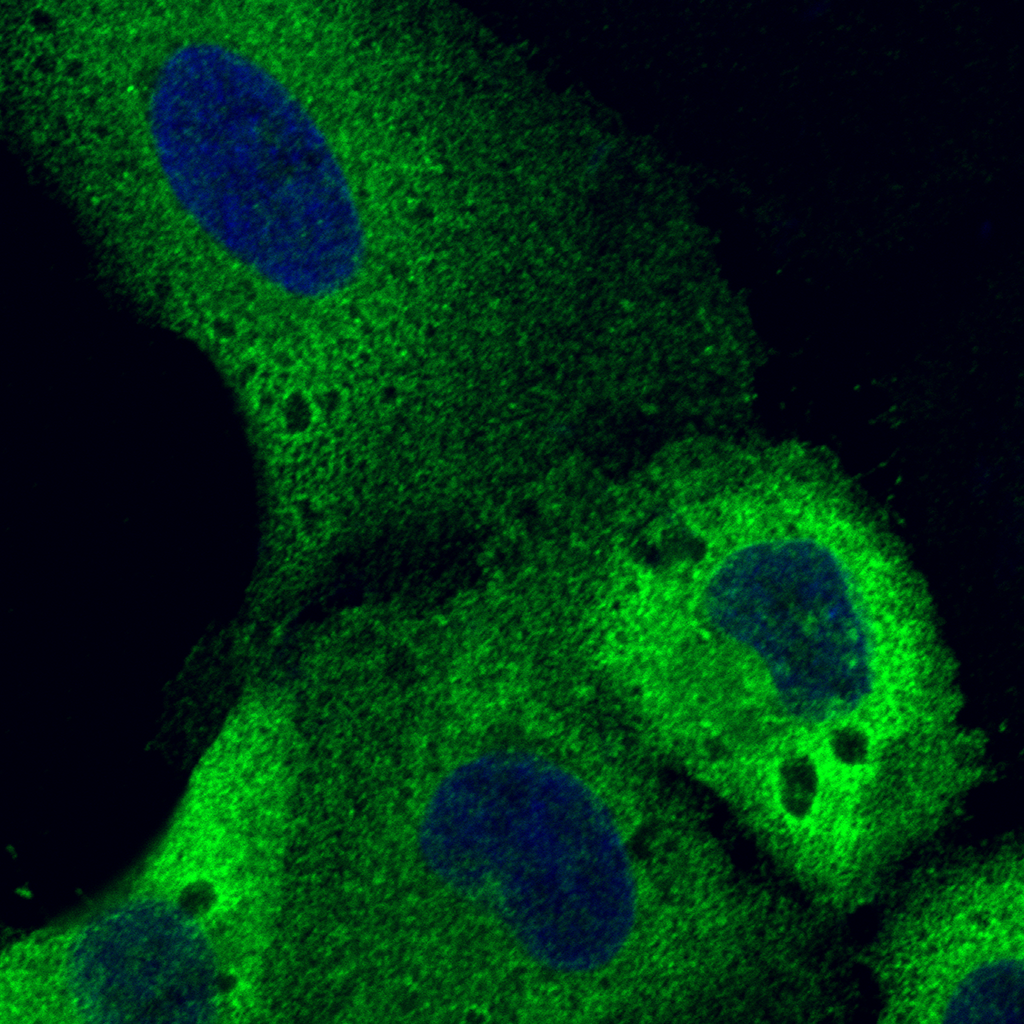

Supplement: Supplementary file 10 — Source data Fig. 2 [file 44319_2025_659_MOESM10_ESM.zip › Figure 2/2G/r-TFE3-L Control.tif]

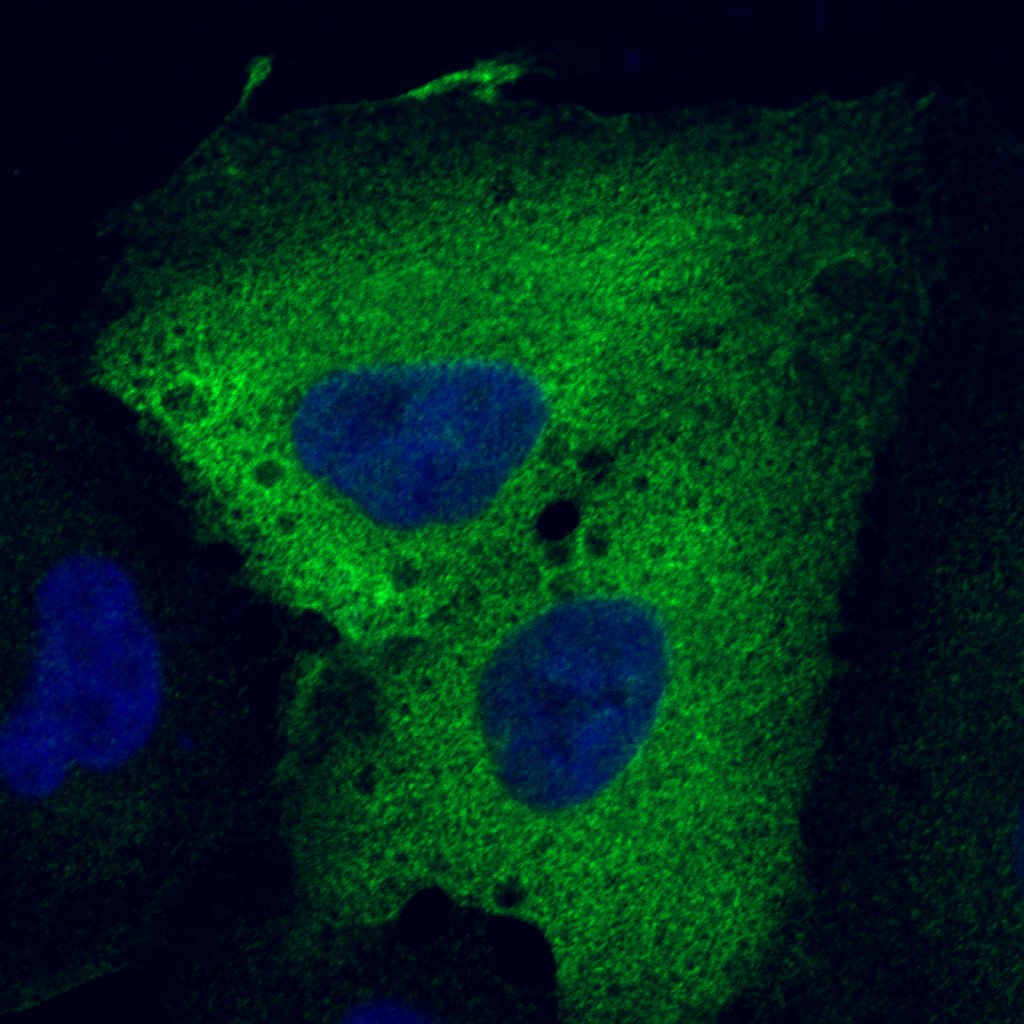

Supplement: Supplementary file 10 — Source data Fig. 2 [file 44319_2025_659_MOESM10_ESM.zip › Figure 2/2G/r-TFE3-M106A Control.tif]

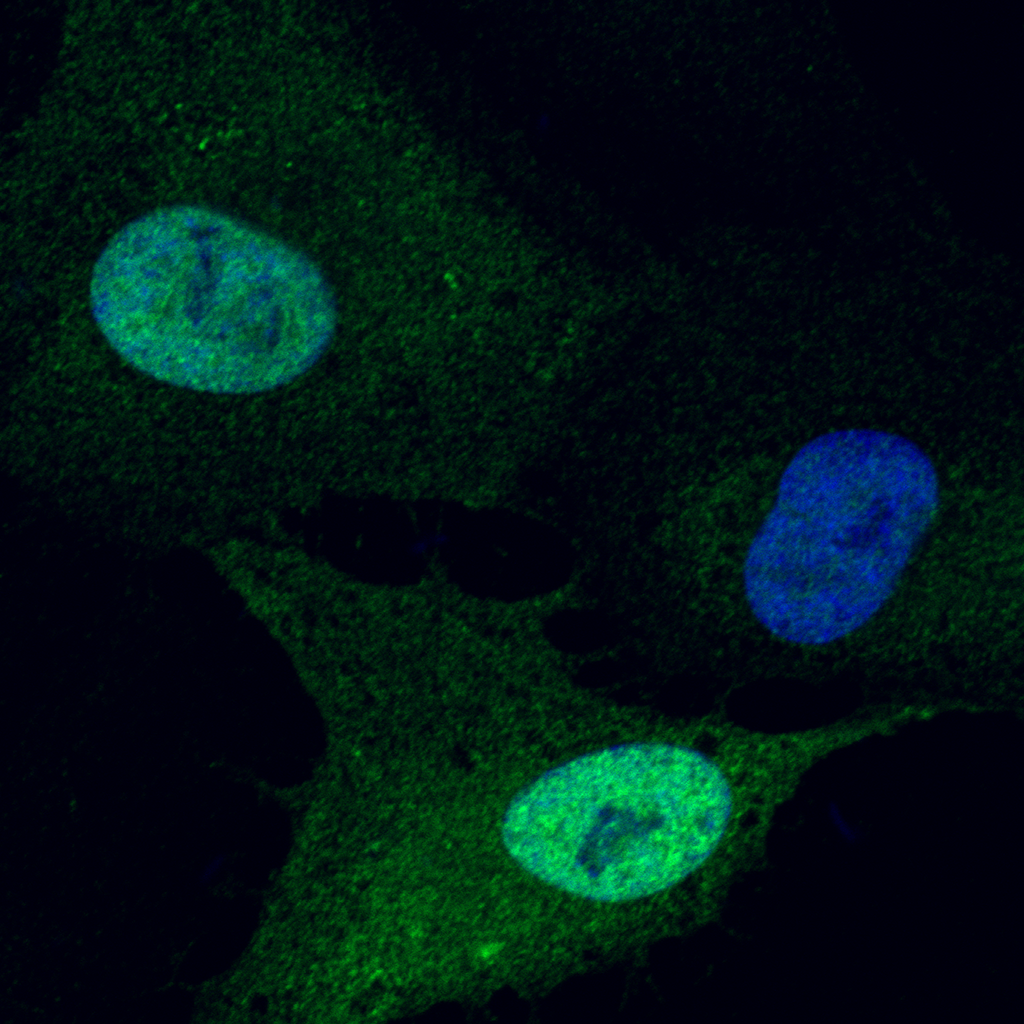

Supplement: Supplementary file 10 — Source data Fig. 2 [file 44319_2025_659_MOESM10_ESM.zip › Figure 2/2G/r-TFE3-S EBSS 8h.tif]

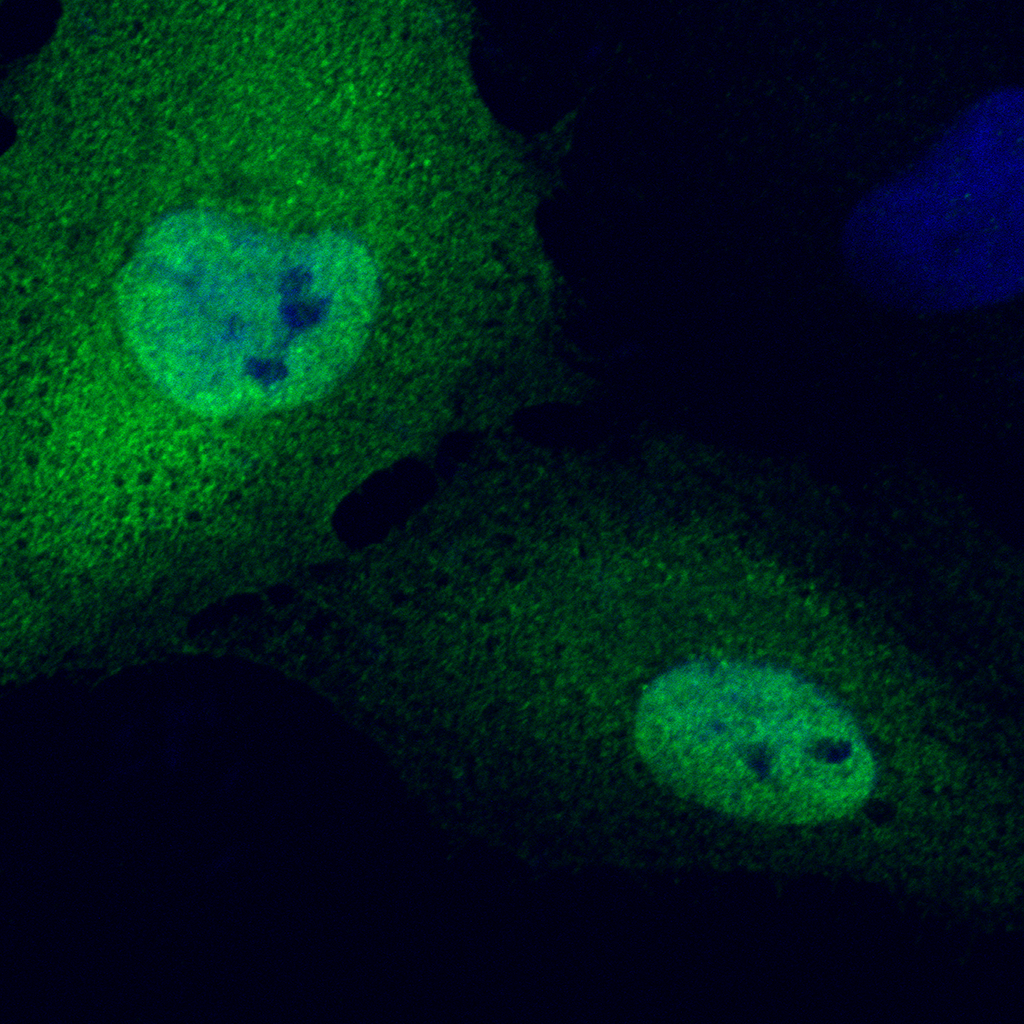

Supplement: Supplementary file 10 — Source data Fig. 2 [file 44319_2025_659_MOESM10_ESM.zip › Figure 2/2G/r-TFE3-L EBSS 8h.tif]

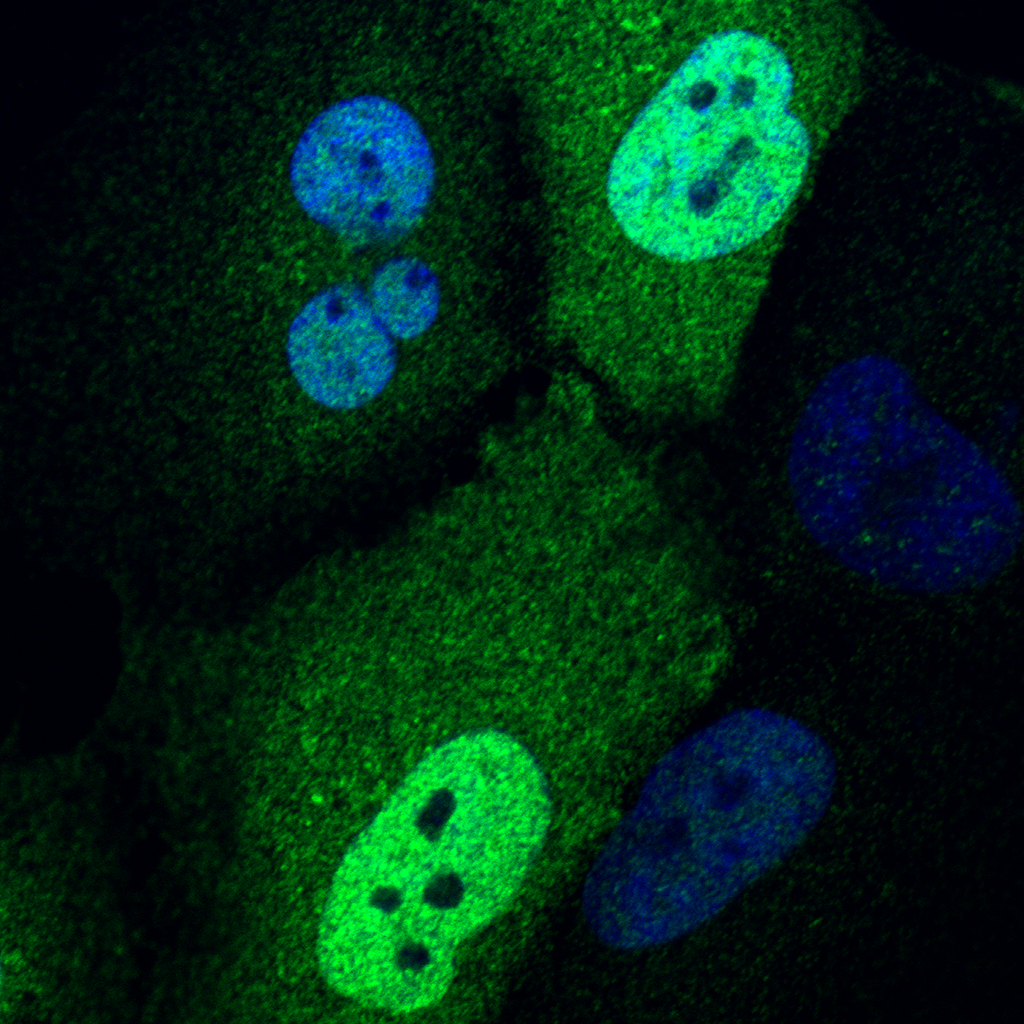

Supplement: Supplementary file 10 — Source data Fig. 2 [file 44319_2025_659_MOESM10_ESM.zip › Figure 2/2G/r-TFE3-M106A EBSS 8h.tif]

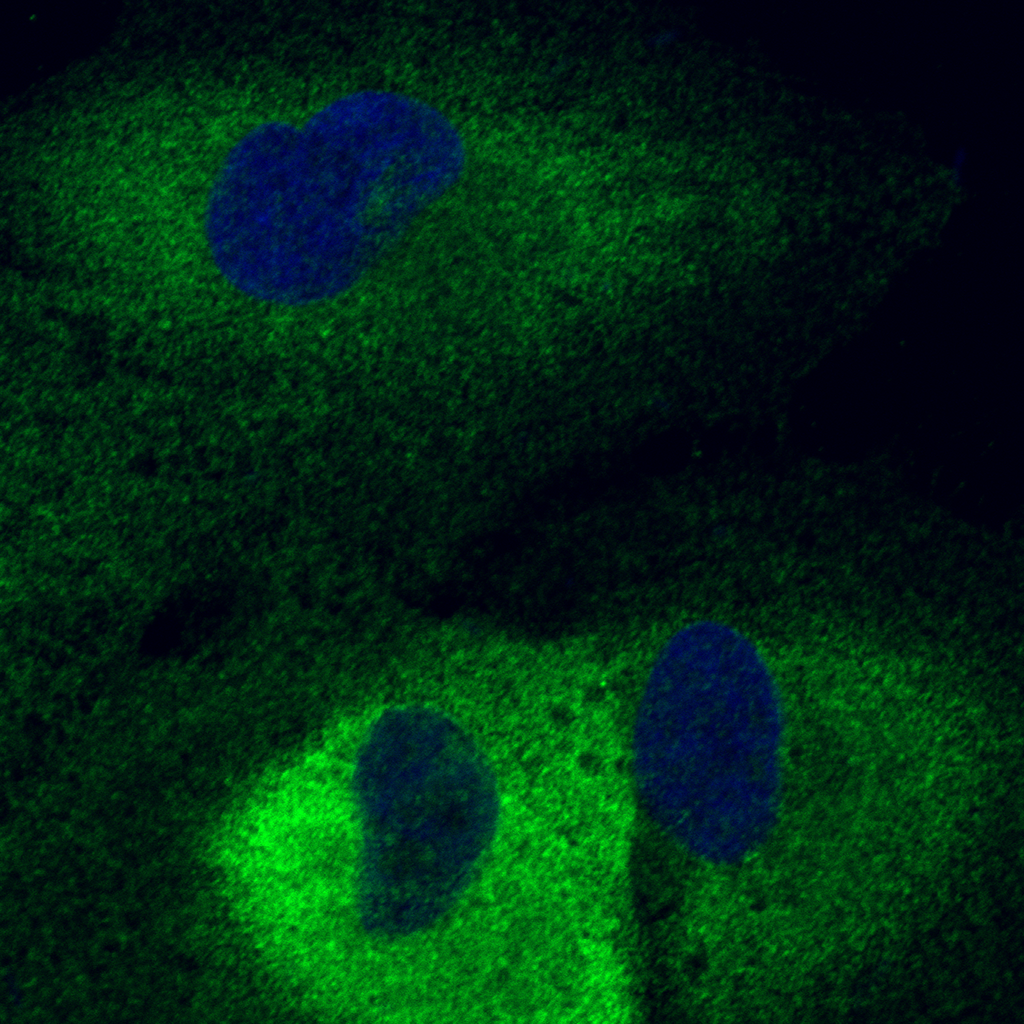

Supplement: Supplementary file 10 — Source data Fig. 2 [file 44319_2025_659_MOESM10_ESM.zip › Figure 2/2G/r-TFE3-S Control.tif]

Source Data Fig. 2F

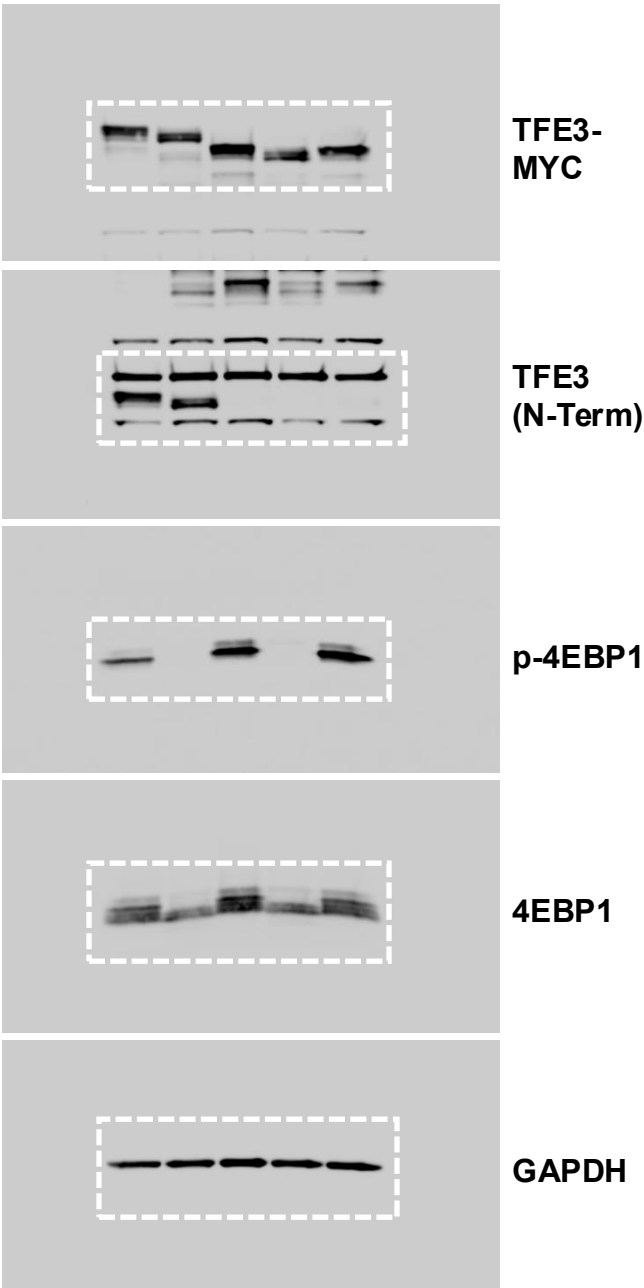

Supplement: Supplementary file 10 — Source data Fig. 2 [file 44319_2025_659_MOESM10_ESM.zip › Figure 2/2F/Source Data Fig 2F.pdf]

Source Data Fig. 2B

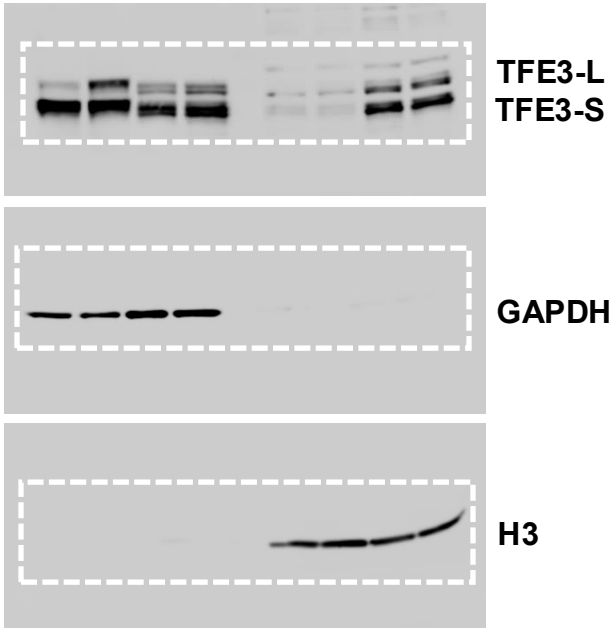

Supplement: Supplementary file 10 — Source data Fig. 2 [file 44319_2025_659_MOESM10_ESM.zip › Figure 2/2B/Source Data Fig 2B.pdf]

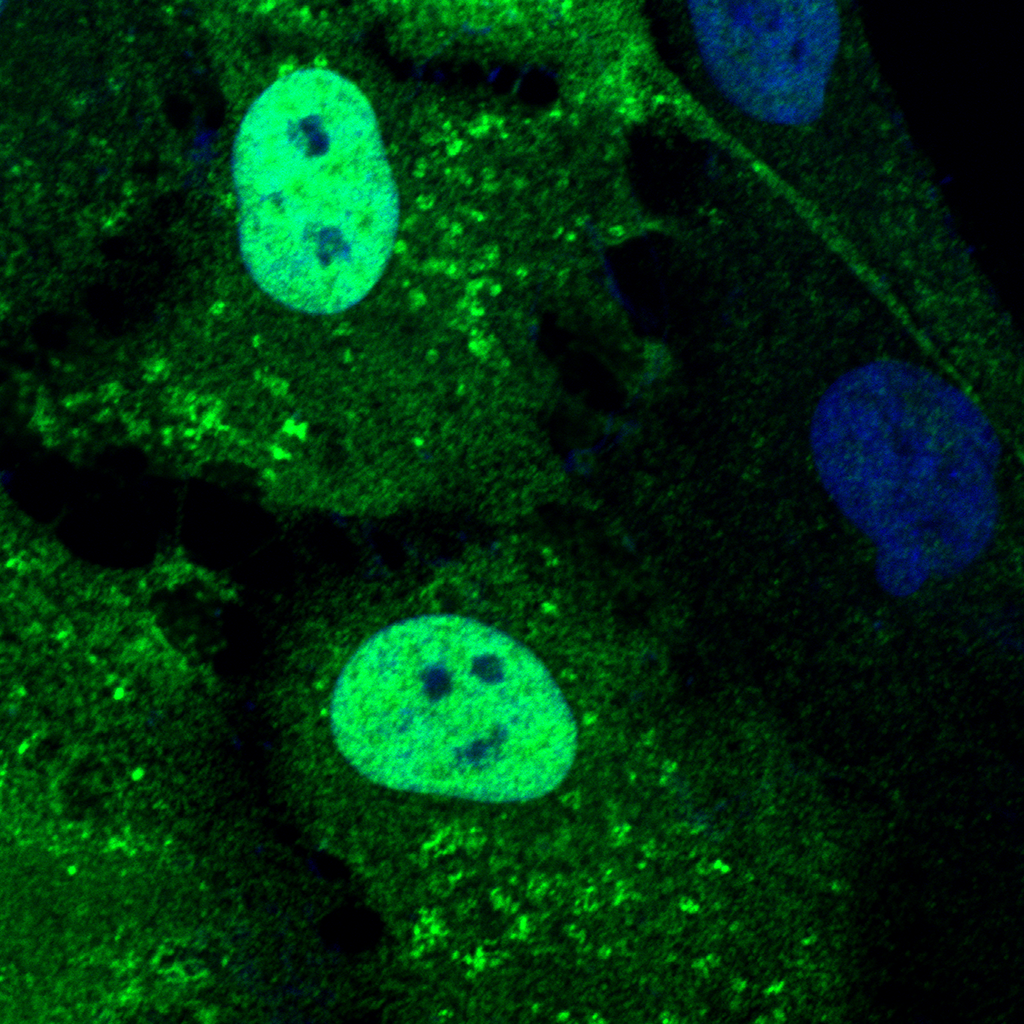

Supplement: Supplementary file 10 — Source data Fig. 2 [file 44319_2025_659_MOESM10_ESM.zip › Figure 2/2E/rTFE3-L Torin1 8h .tif]

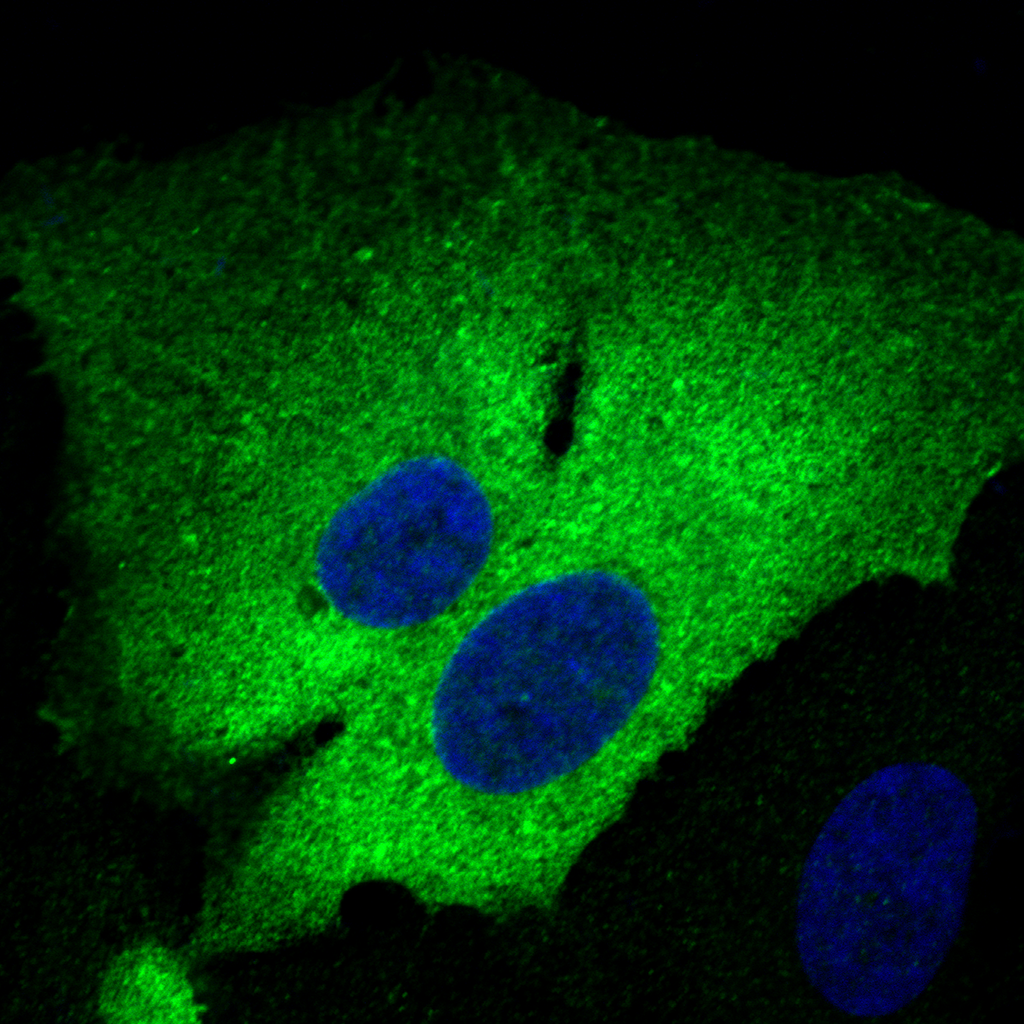

Supplement: Supplementary file 10 — Source data Fig. 2 [file 44319_2025_659_MOESM10_ESM.zip › Figure 2/2E/rTFE3-L Control.tif]

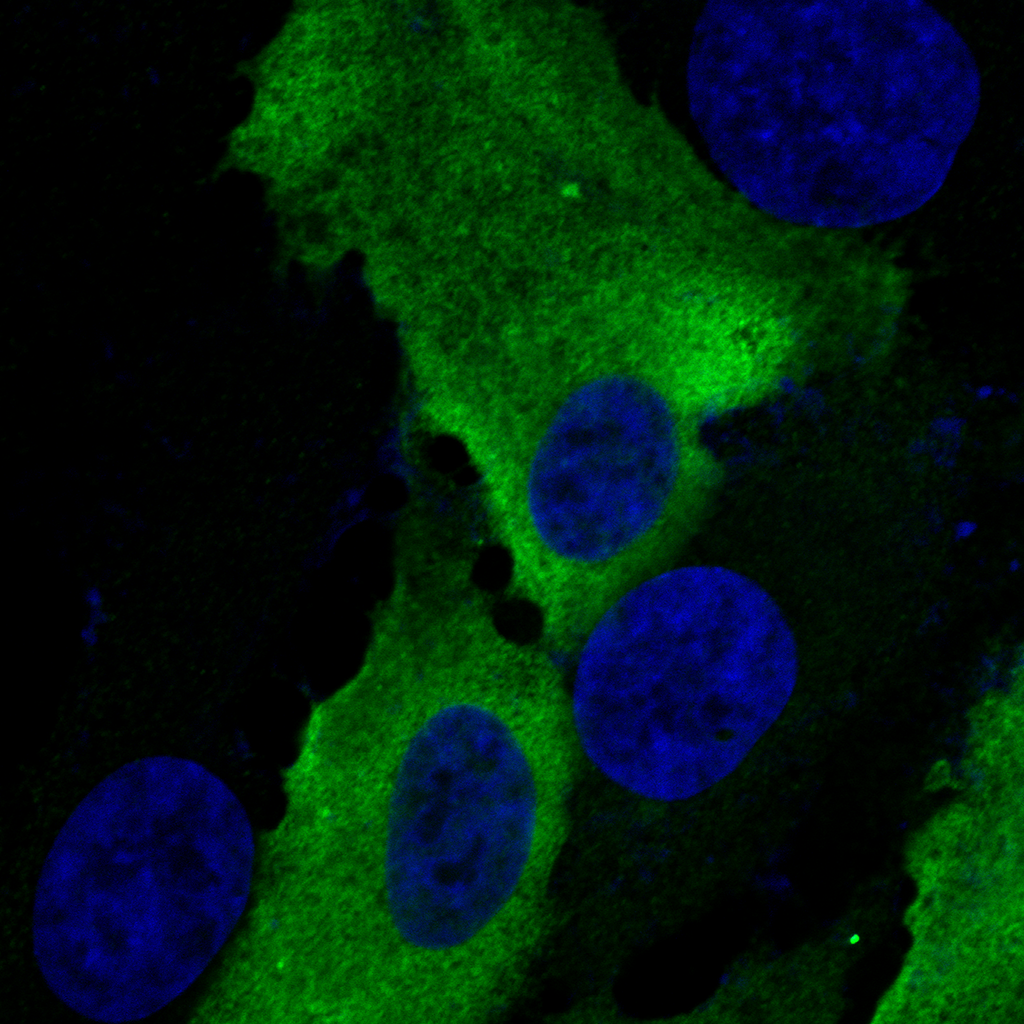

Supplement: Supplementary file 10 — Source data Fig. 2 [file 44319_2025_659_MOESM10_ESM.zip › Figure 2/2E/rTFE3-M1A Control.tif]

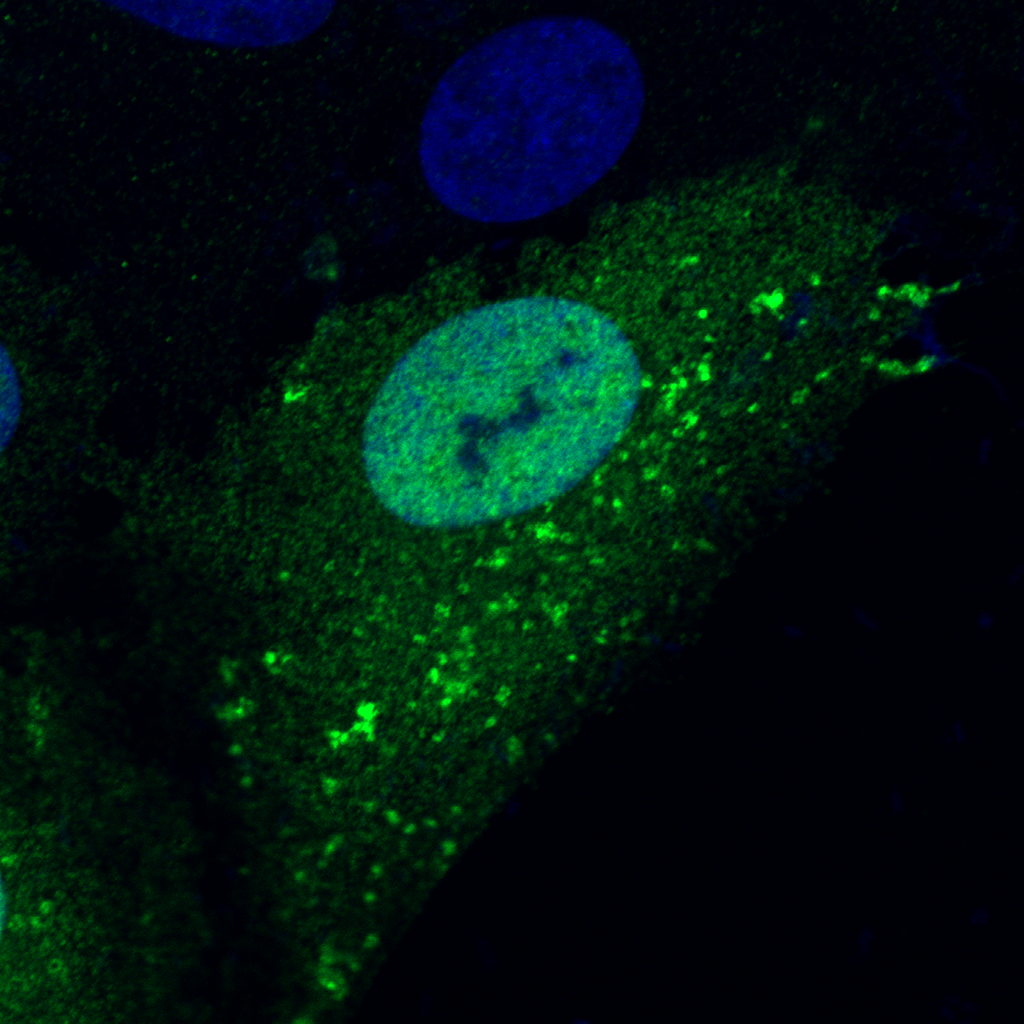

Supplement: Supplementary file 10 — Source data Fig. 2 [file 44319_2025_659_MOESM10_ESM.zip › Figure 2/2E/rTFE3-M1A Torin1 8h.tif]

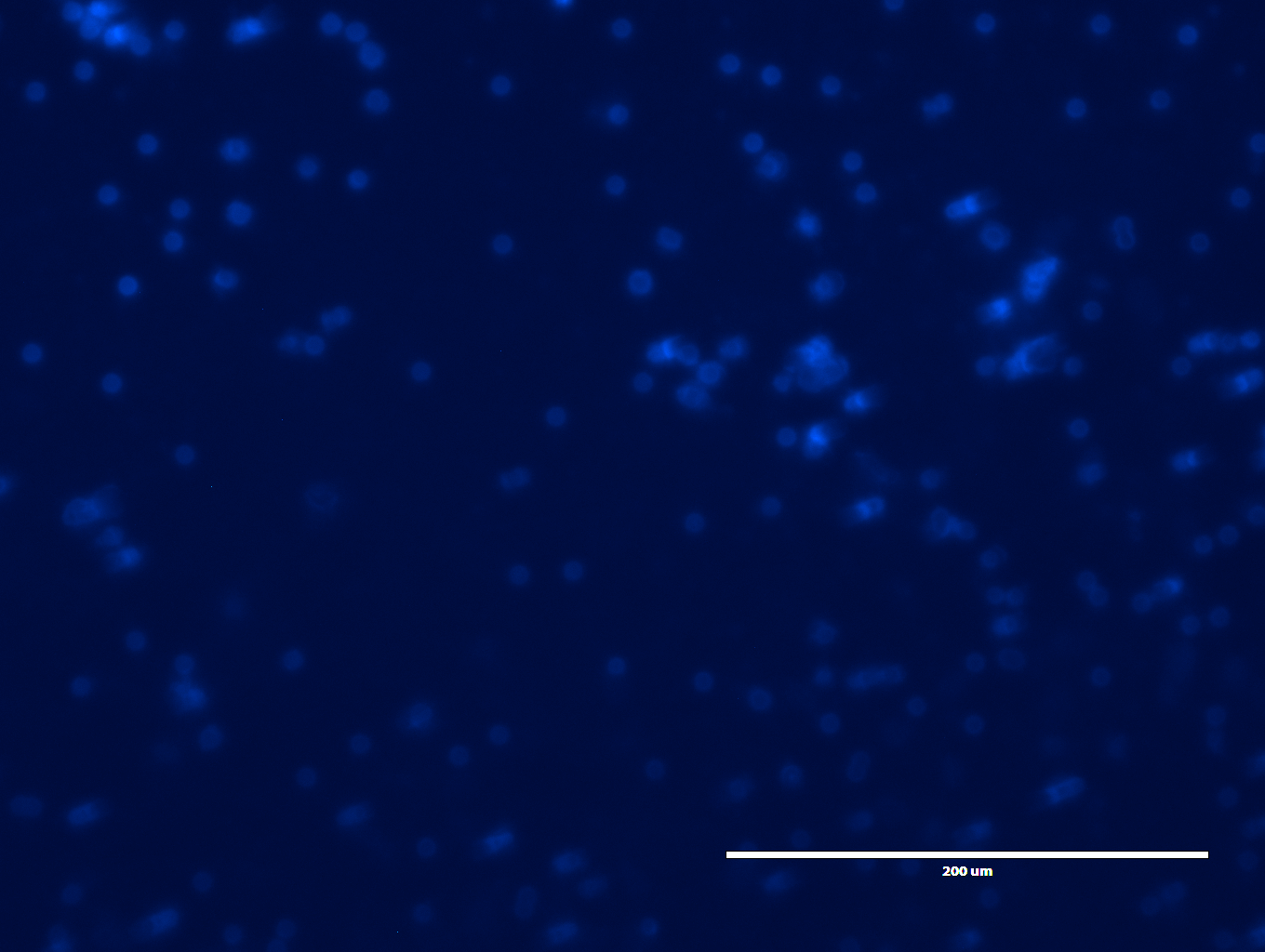

Supplement: Supplementary file 12 — Source data Fig. 4 [file 44319_2025_659_MOESM12_ESM.zip › Figure 4/4F/ARPE19-AdNull.tif]

Source Data. Figure 4F

ARPE19

Ad-Null

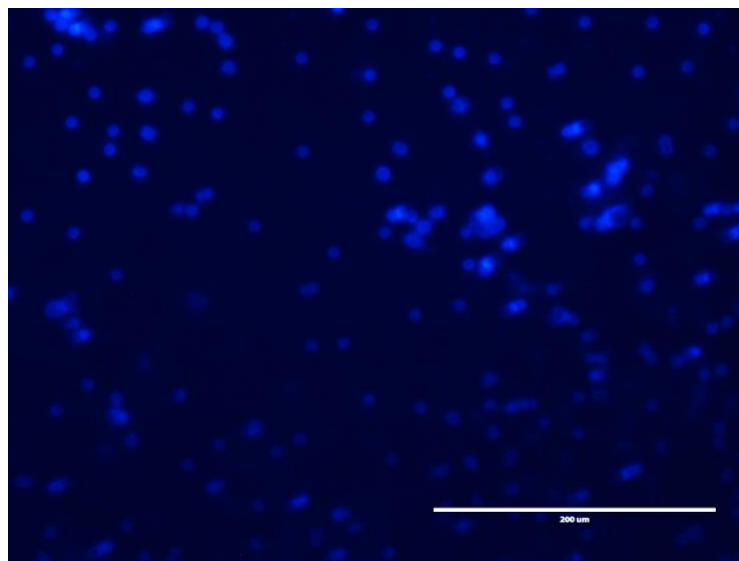

ARPE19

Ad-rTFE3-L

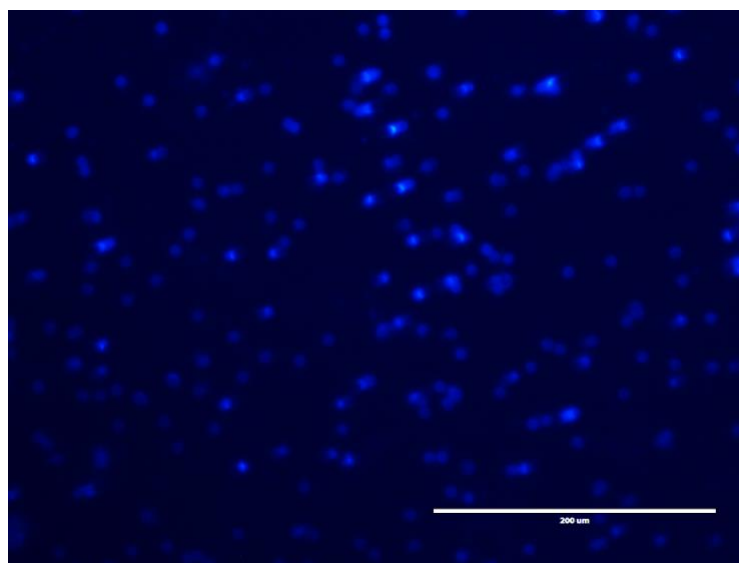

ARPE19

Ad-rTFE3-S

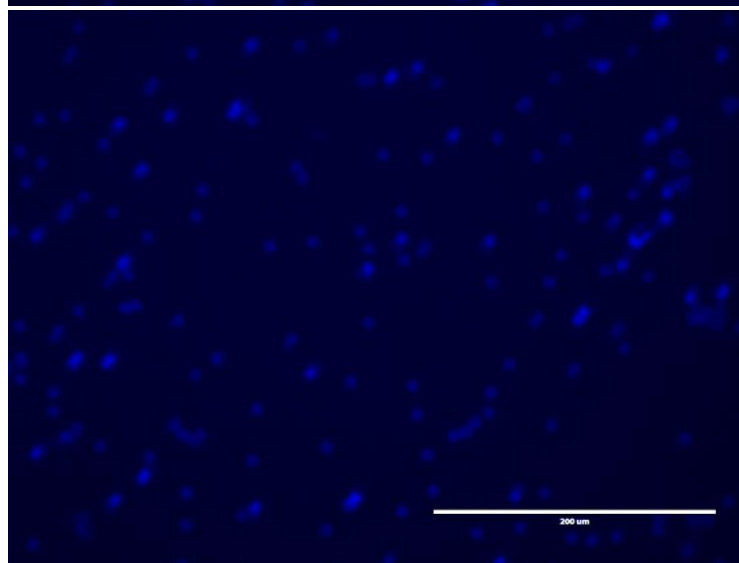

Supplement: Supplementary file 12 — Source data Fig. 4 [file 44319_2025_659_MOESM12_ESM.zip › Figure 4/4F/Combined Source Data 4F.pdf]

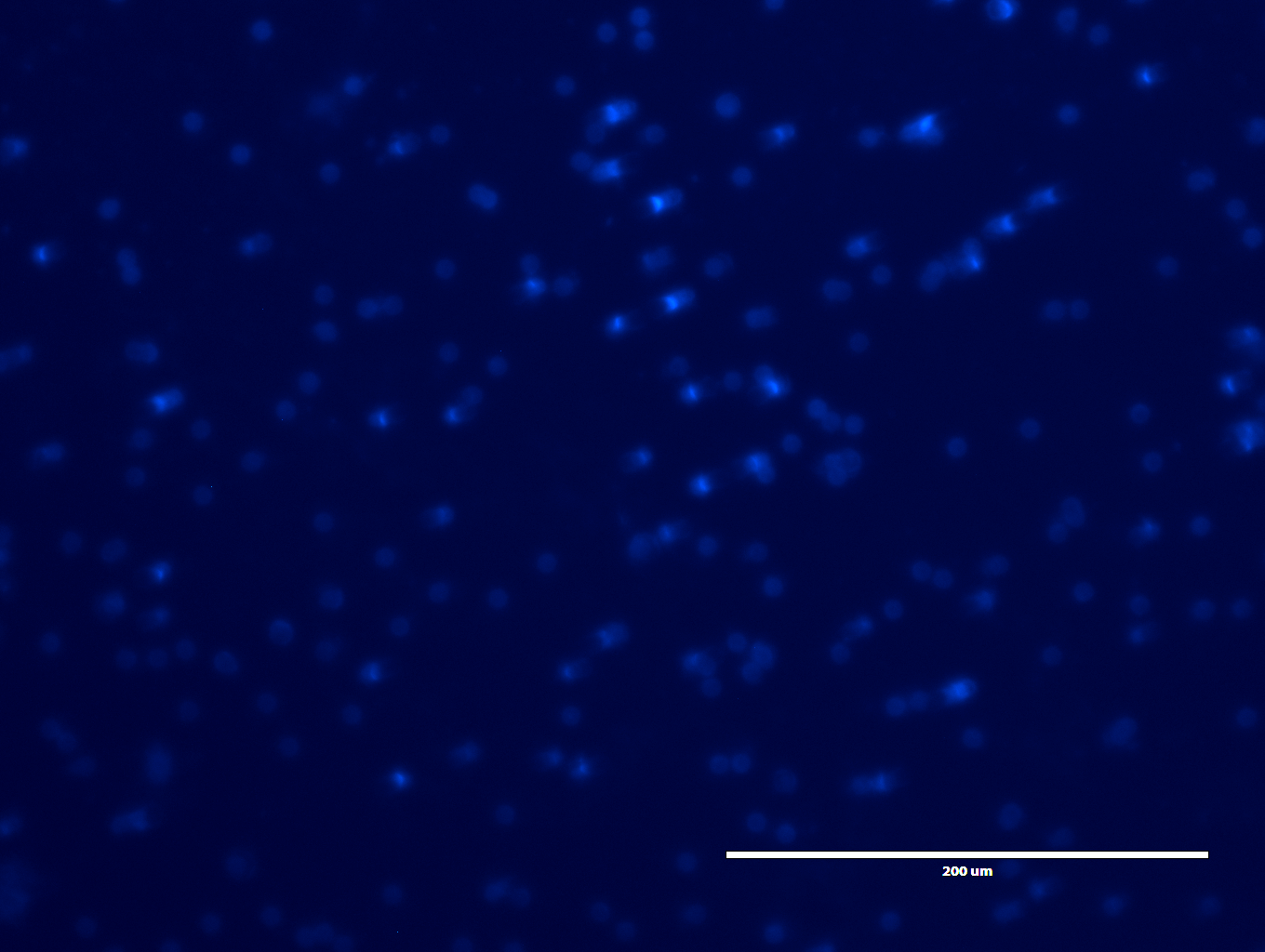

Supplement: Supplementary file 12 — Source data Fig. 4 [file 44319_2025_659_MOESM12_ESM.zip › Figure 4/4F/ARPE19-AdrTFE3L.tif]

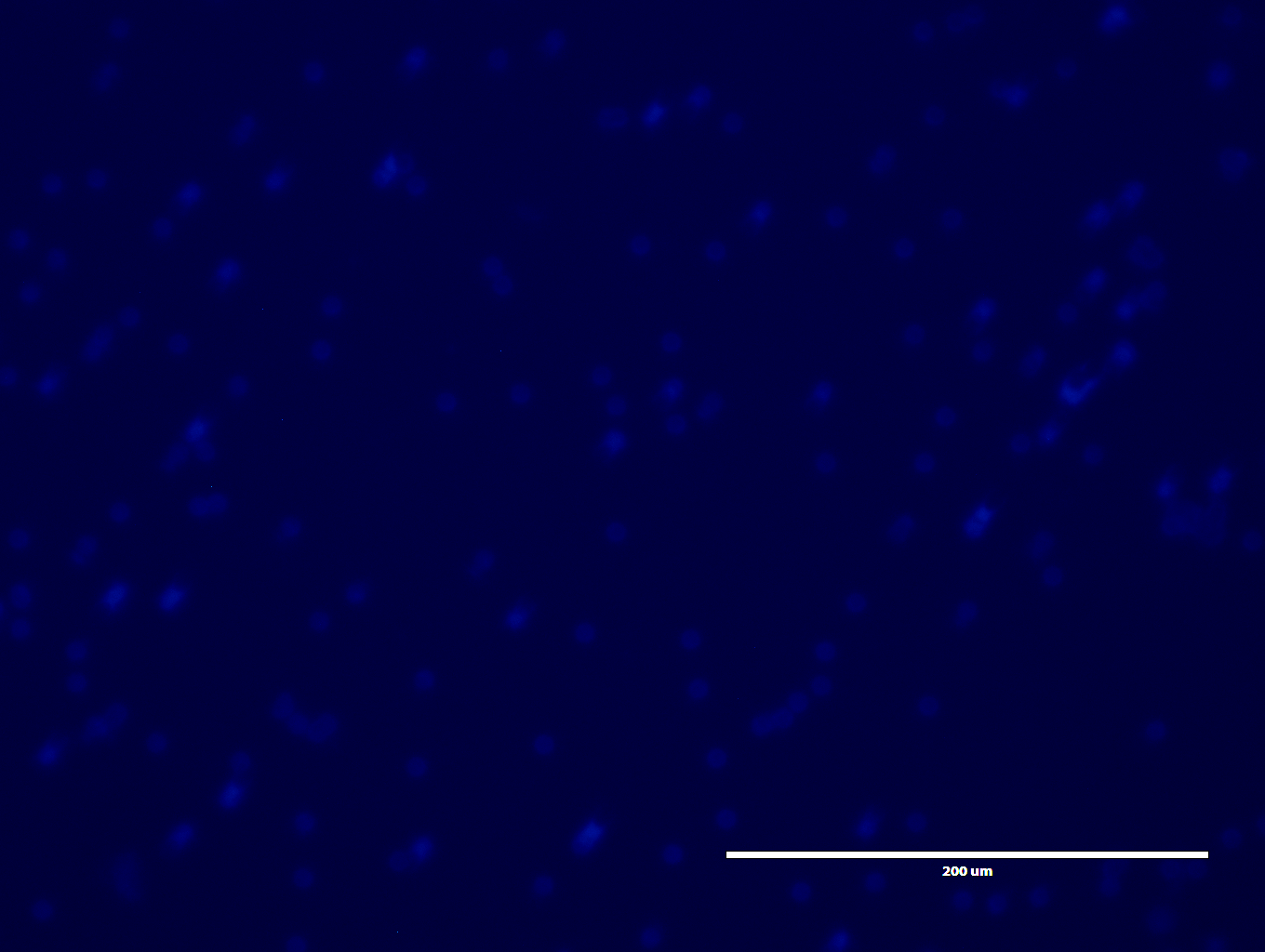

Supplement: Supplementary file 12 — Source data Fig. 4 [file 44319_2025_659_MOESM12_ESM.zip › Figure 4/4F/ARPE19-ADrTFE3S.tif]

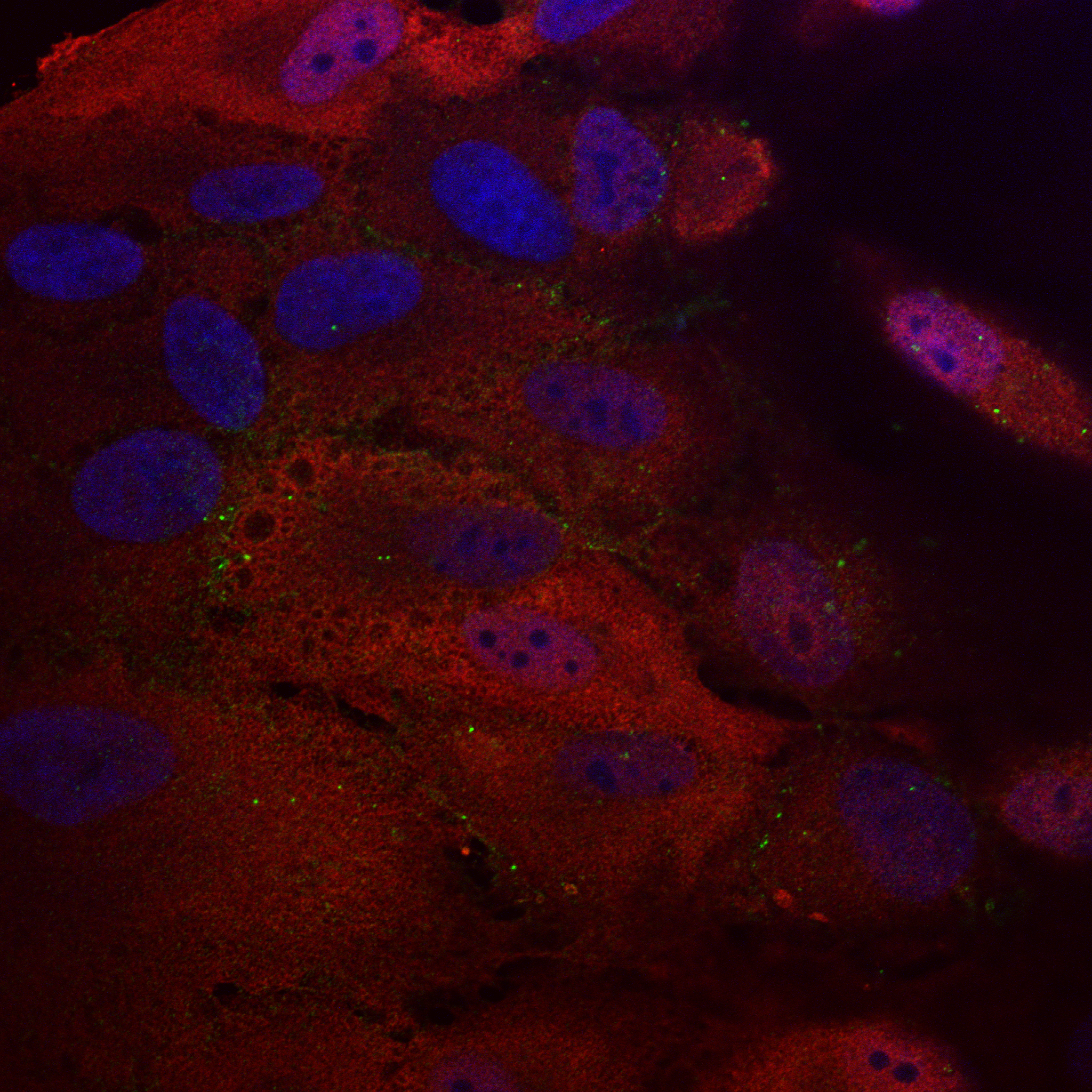

Supplement: Supplementary file 12 — Source data Fig. 4 [file 44319_2025_659_MOESM12_ESM.zip › Figure 4/4C/Ad-rTFE3-S.tif]

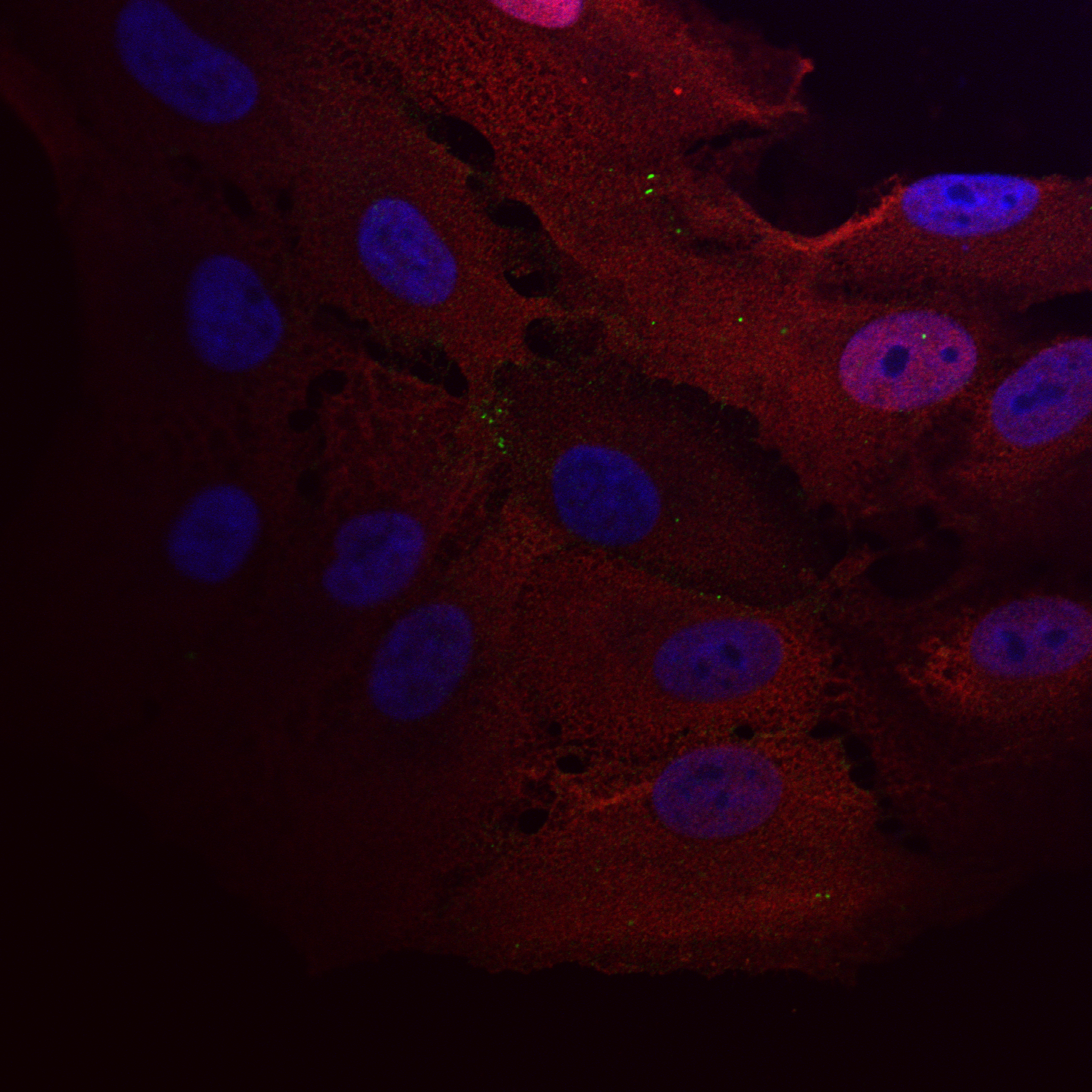

Supplement: Supplementary file 12 — Source data Fig. 4 [file 44319_2025_659_MOESM12_ESM.zip › Figure 4/4C/Ad-rTFE3-L.tif]

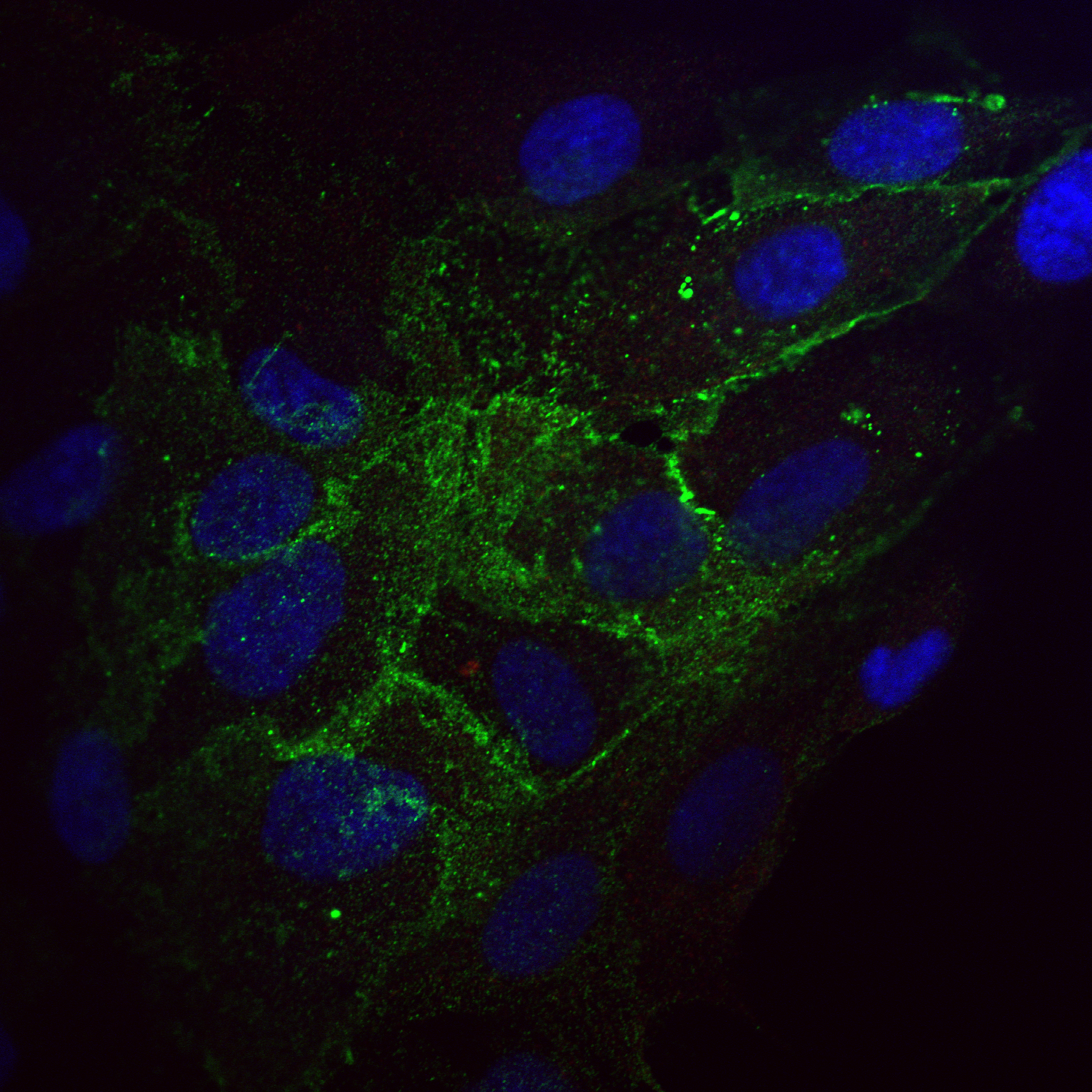

Supplement: Supplementary file 12 — Source data Fig. 4 [file 44319_2025_659_MOESM12_ESM.zip › Figure 4/4C/Ad-Null.tif]

Source Data Fig. 5G

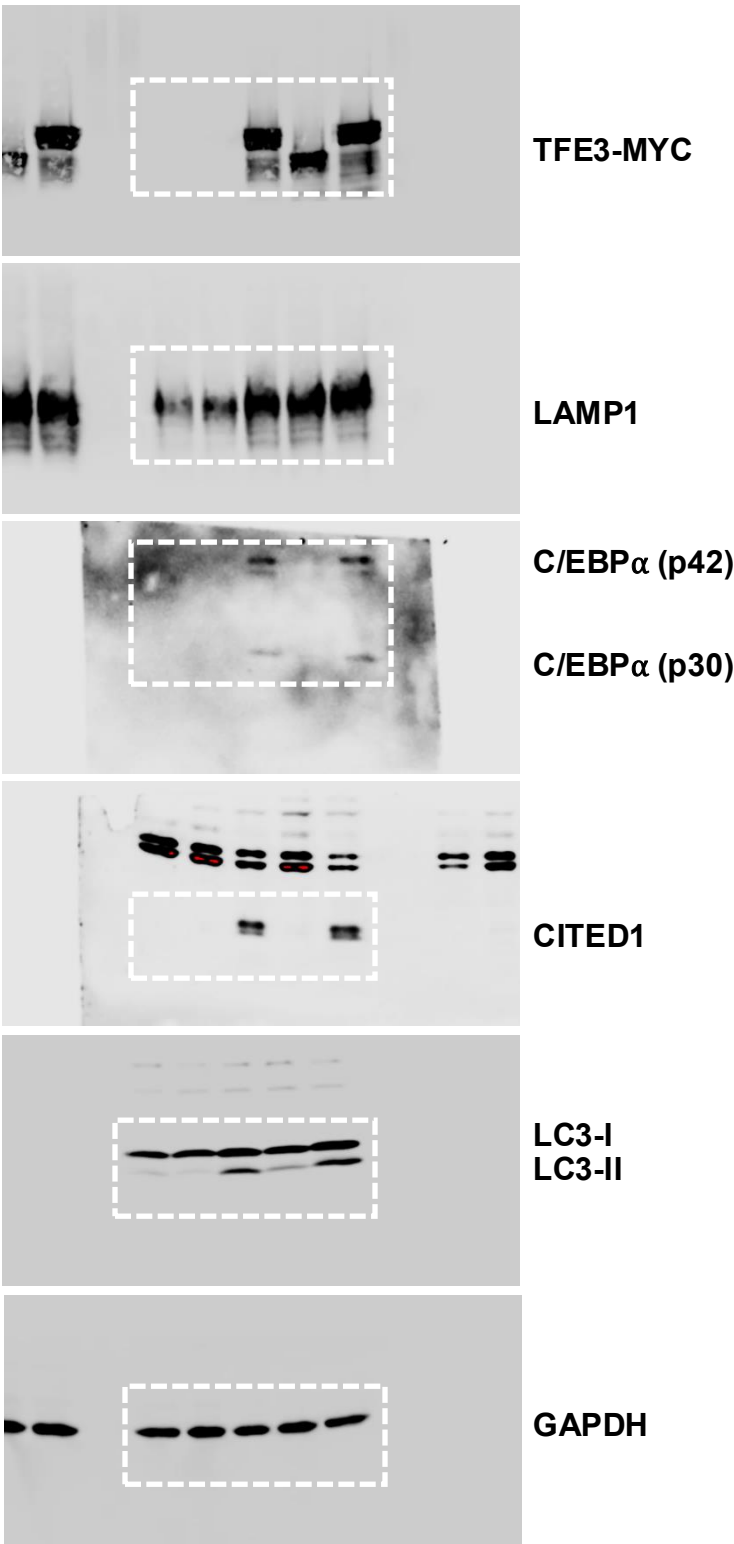

Supplement: Supplementary file 13 — Source data Fig. 5 [file 44319_2025_659_MOESM13_ESM.zip › Figure 5/5G/Source Data Fig 5G.pdf]

Source Data Fig. 5C

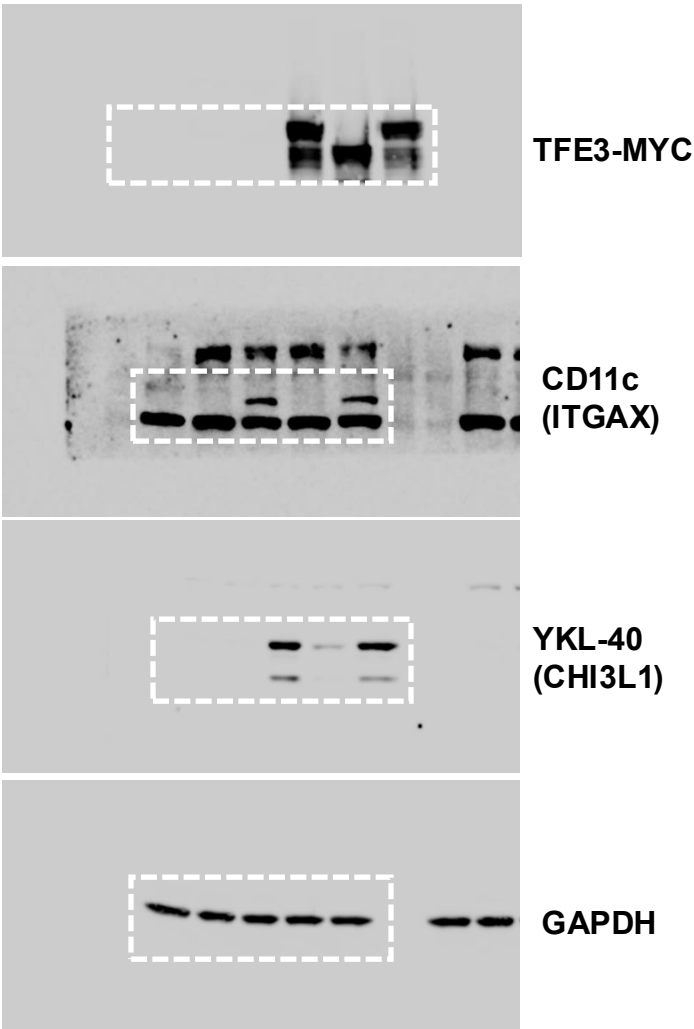

Supplement: Supplementary file 13 — Source data Fig. 5 [file 44319_2025_659_MOESM13_ESM.zip › Figure 5/5C/Source Data Fig 5C.pdf]

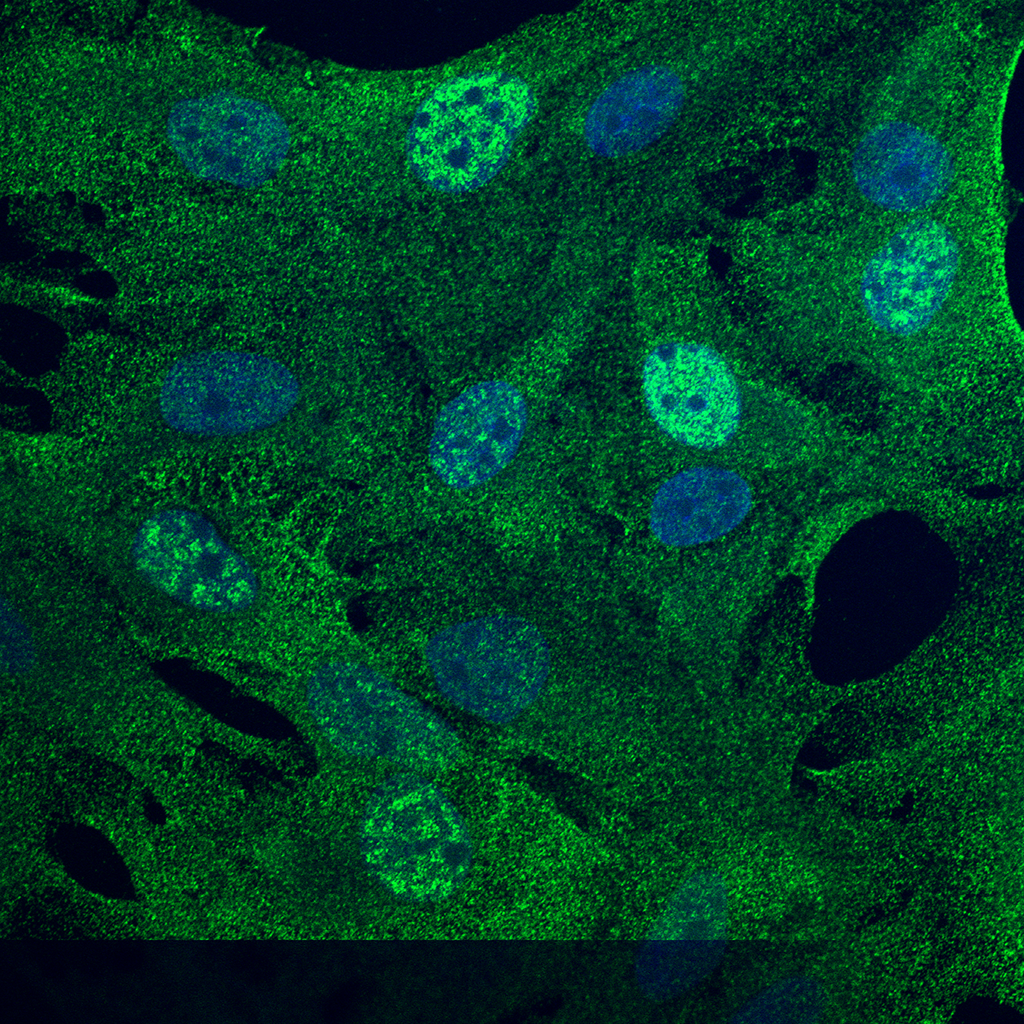

Supplement: Supplementary file 13 — Source data Fig. 5 [file 44319_2025_659_MOESM13_ESM.zip › Figure 5/5E/Ad-rTFE3-L:M106A.tif]

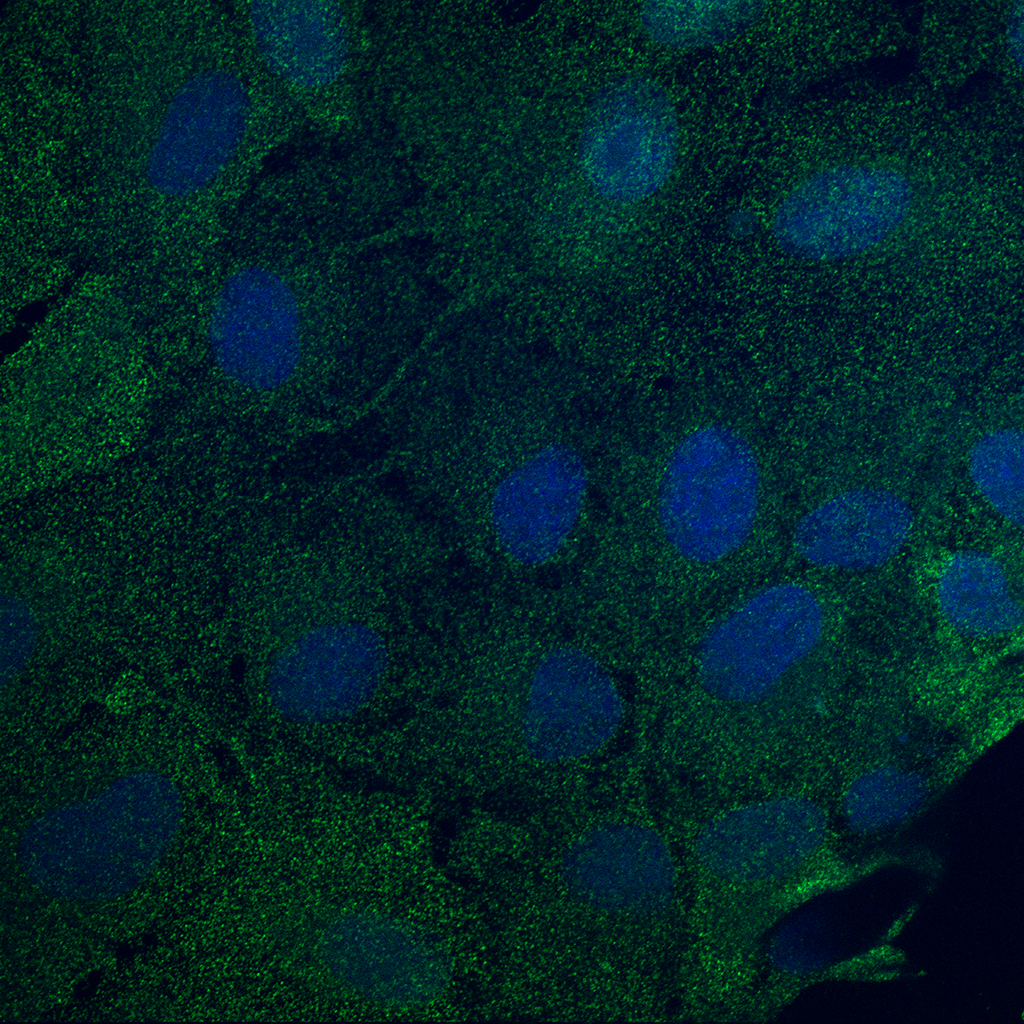

Supplement: Supplementary file 13 — Source data Fig. 5 [file 44319_2025_659_MOESM13_ESM.zip › Figure 5/5E/Ad-rTFE3-S.tif]

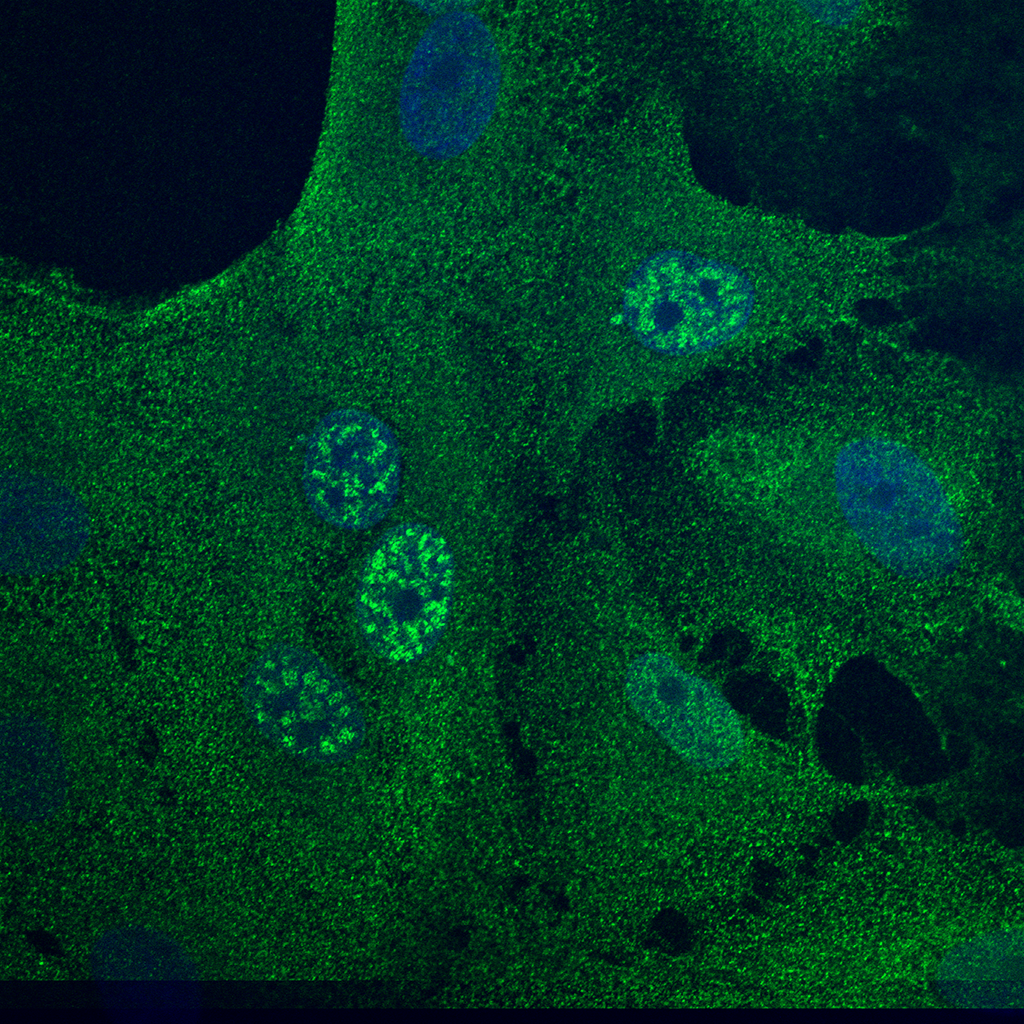

Supplement: Supplementary file 13 — Source data Fig. 5 [file 44319_2025_659_MOESM13_ESM.zip › Figure 5/5E/Ad-rTFE3-L.tif]

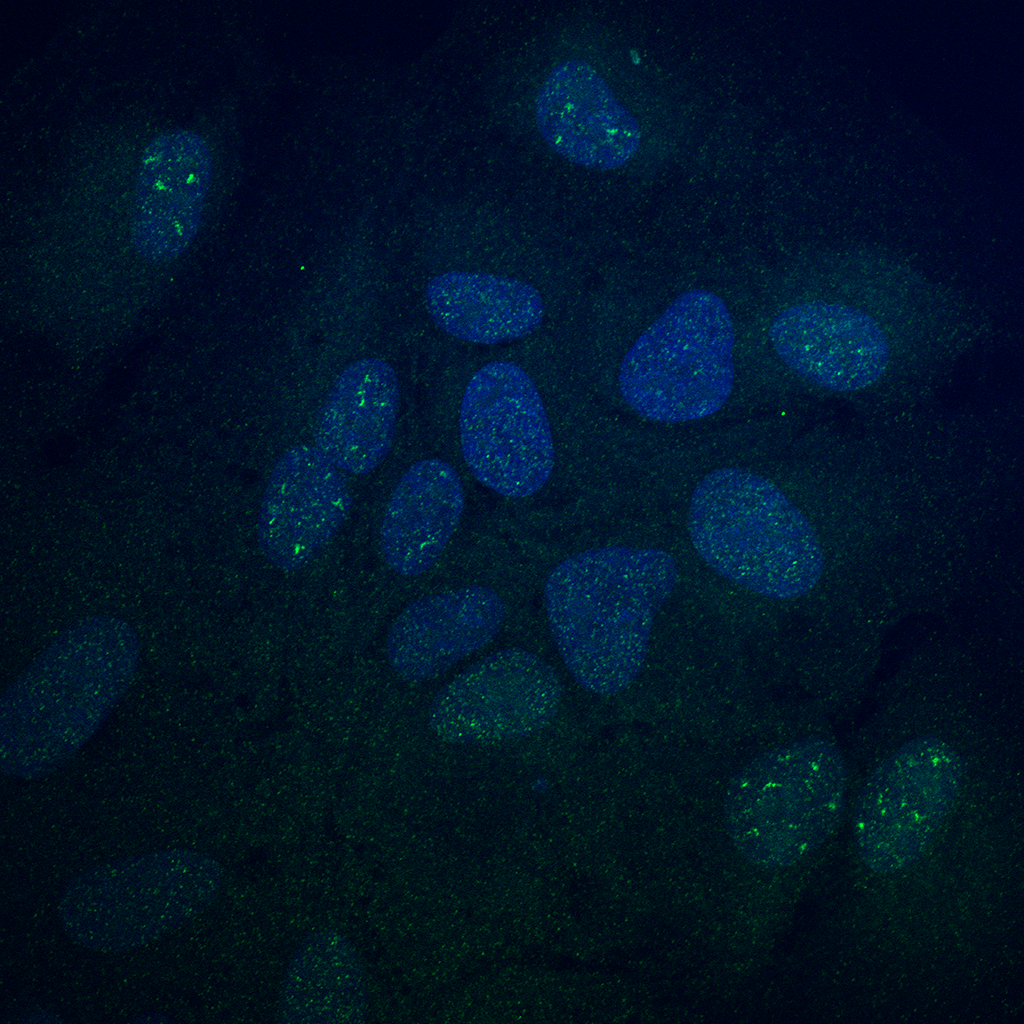

Supplement: Supplementary file 13 — Source data Fig. 5 [file 44319_2025_659_MOESM13_ESM.zip › Figure 5/5E/Ad-Null.tif]

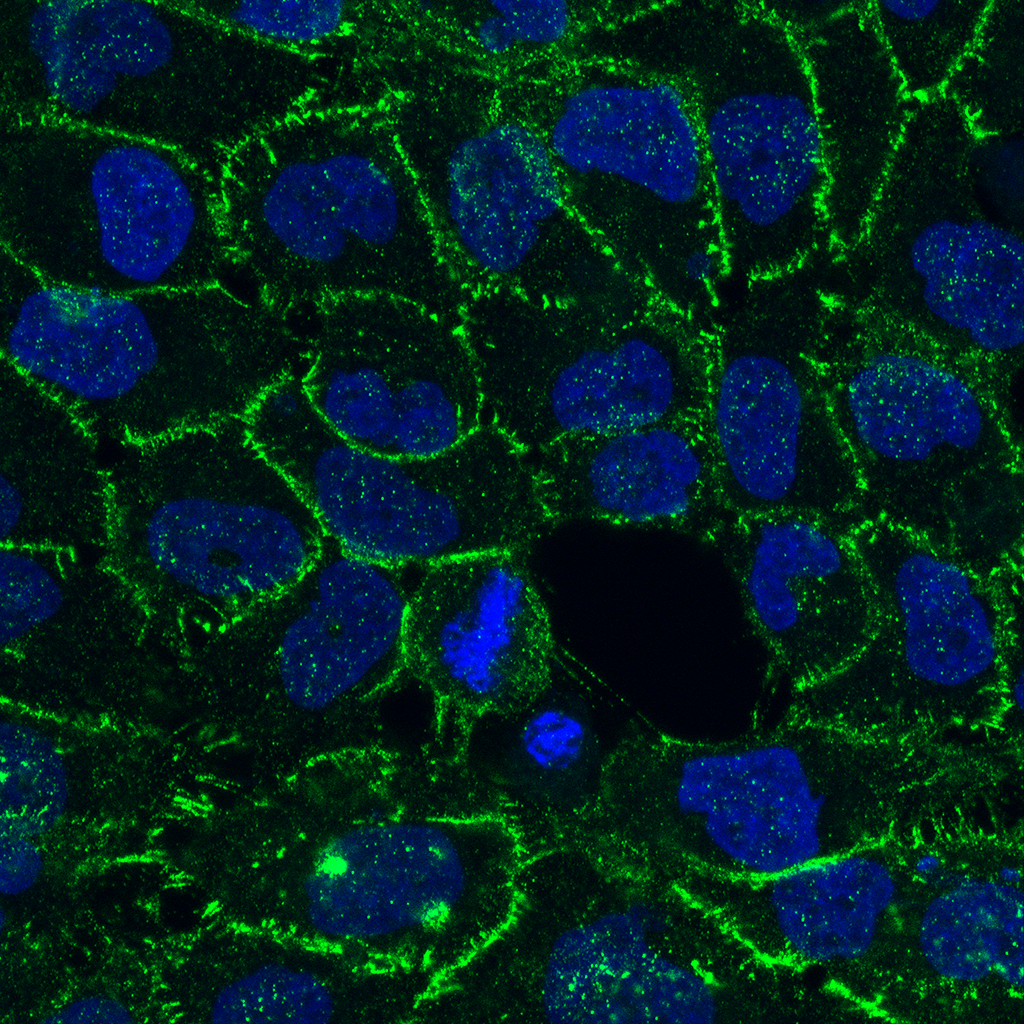

Supplement: Supplementary file 14 — Source data Fig. 6 [file 44319_2025_659_MOESM14_ESM.zip › Figure 6/6C/Source-TSC2KOcrisprTFE3AK-6C.tif]

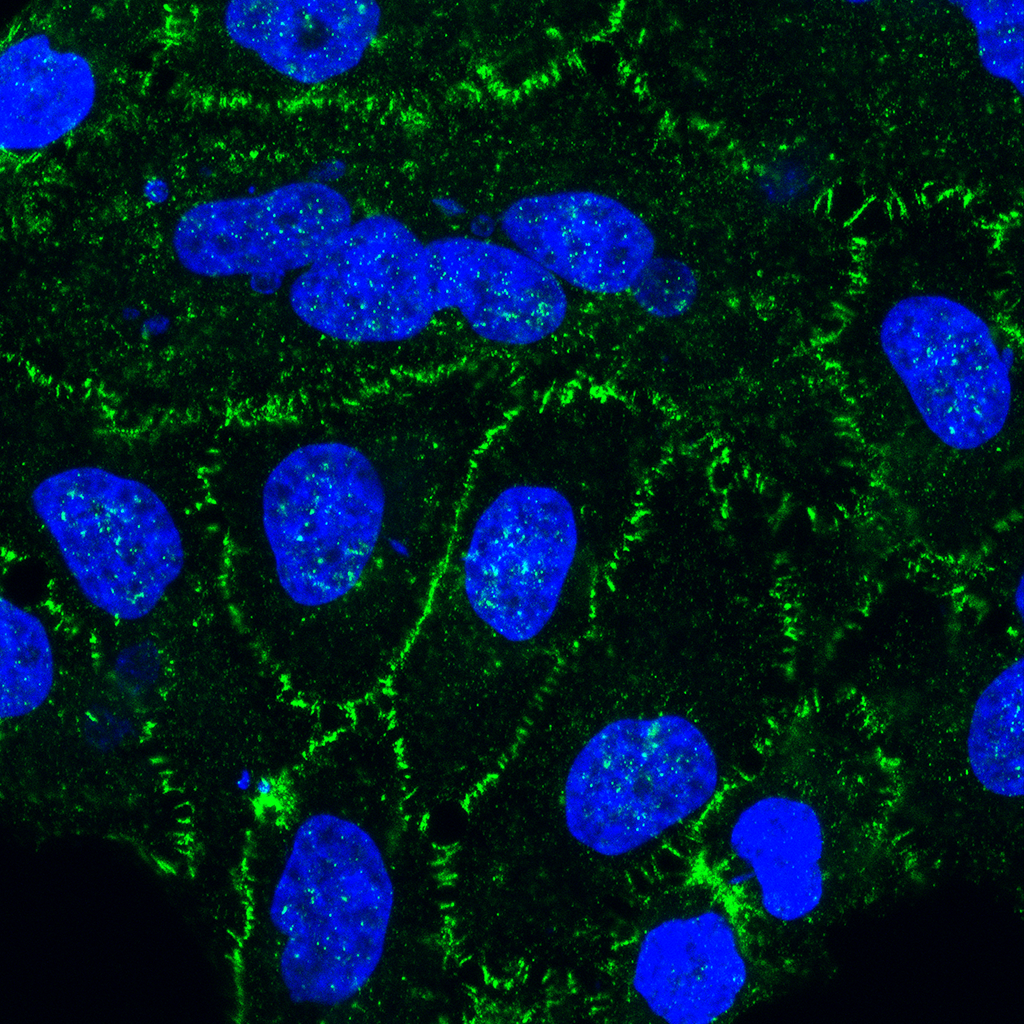

Supplement: Supplementary file 14 — Source data Fig. 6 [file 44319_2025_659_MOESM14_ESM.zip › Figure 6/6C/SourceTSC2KOcrisprCTRL-6C.tif (RGB).tif]

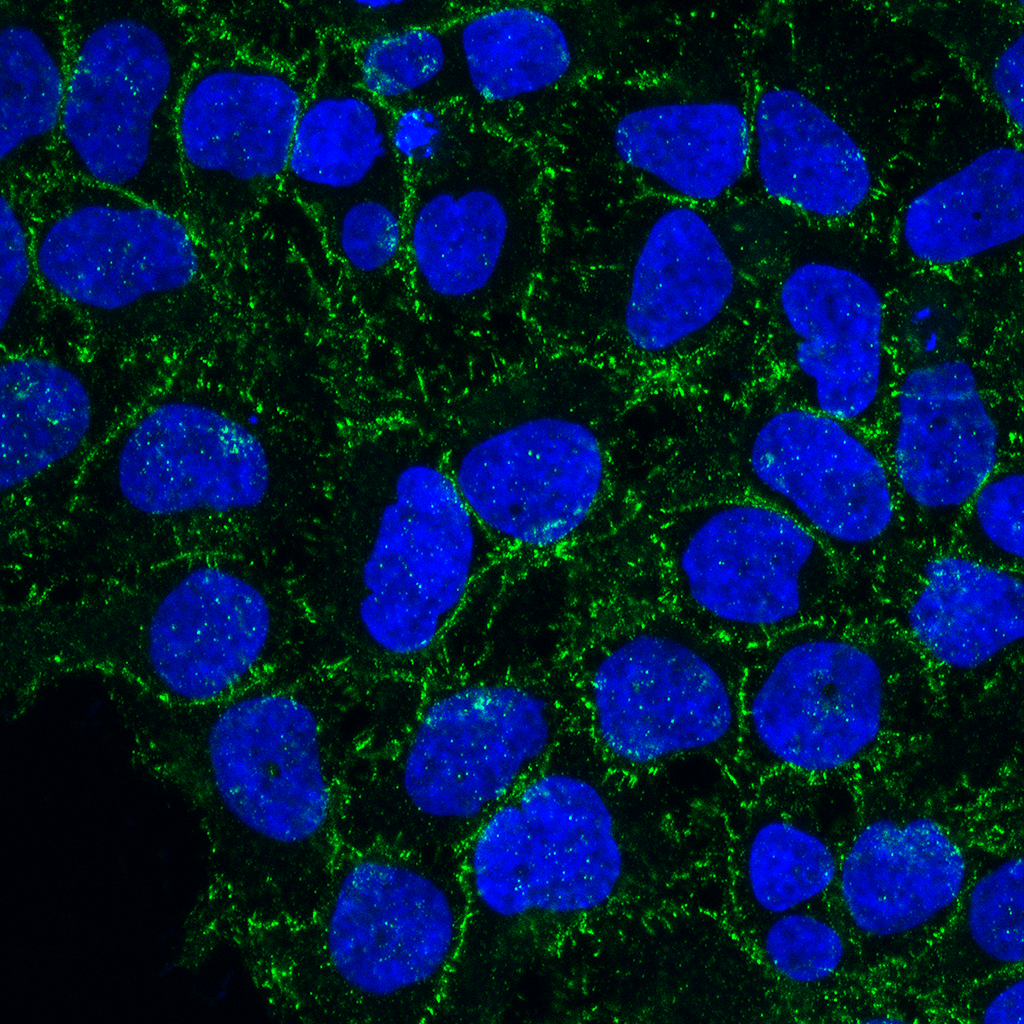

Supplement: Supplementary file 14 — Source data Fig. 6 [file 44319_2025_659_MOESM14_ESM.zip › Figure 6/6C/sourceNcad-6CWTcrisprCTRL.tif]

Source Data Fig. 6A

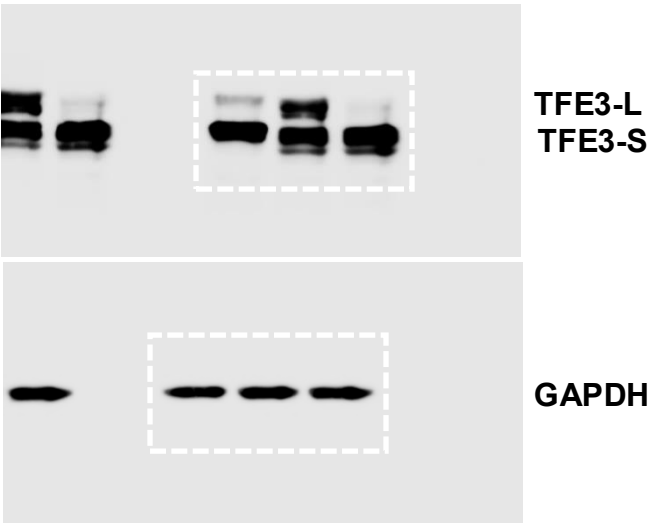

Supplement: Supplementary file 14 — Source data Fig. 6 [file 44319_2025_659_MOESM14_ESM.zip › Figure 6/6A/Source Data Fig 6A.pdf]

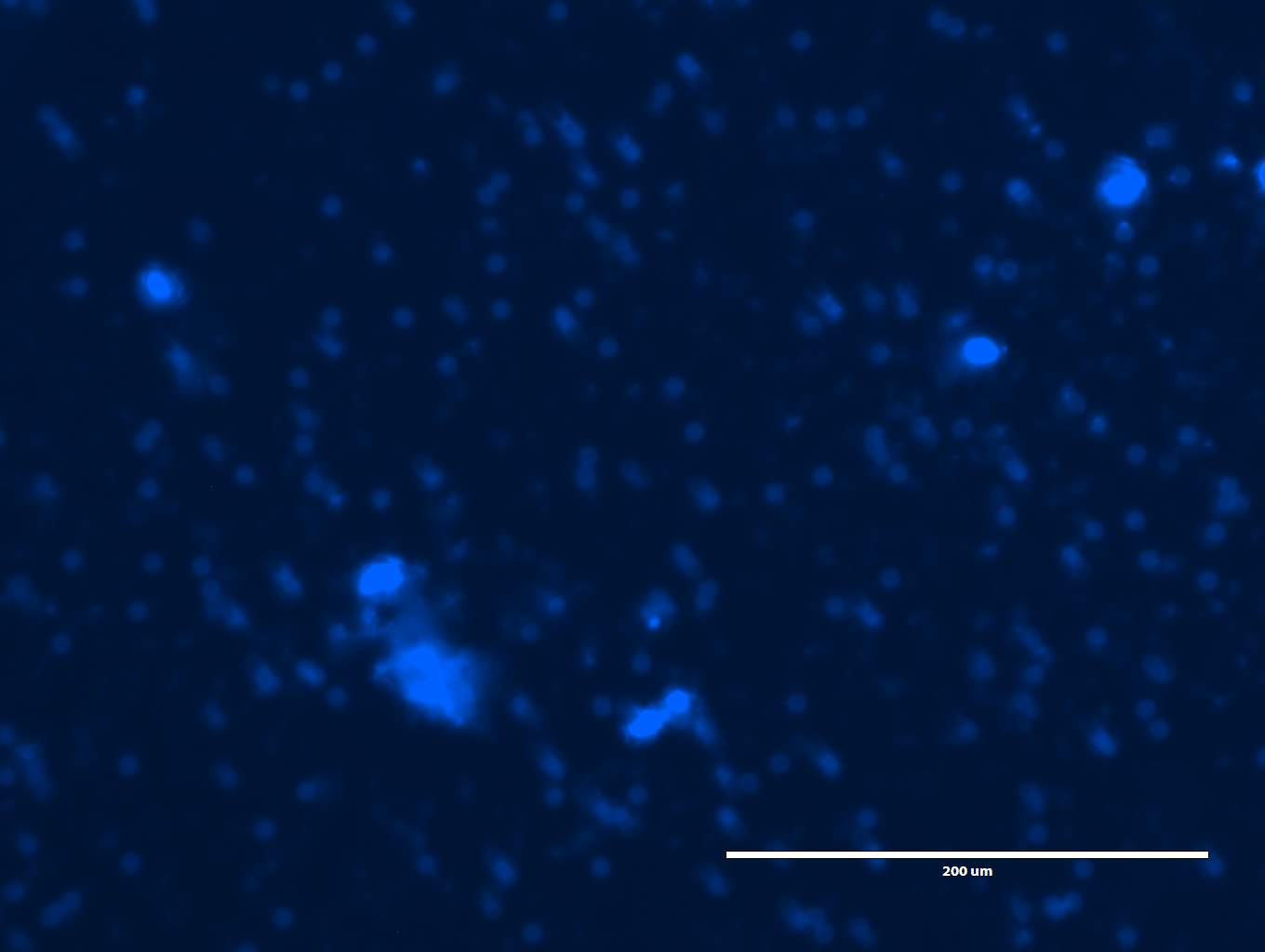

Supplement: Supplementary file 14 — Source data Fig. 6 [file 44319_2025_659_MOESM14_ESM.zip › Figure 6/6D/SourcehelaTSC2KO-CRISPRctrl.tif]

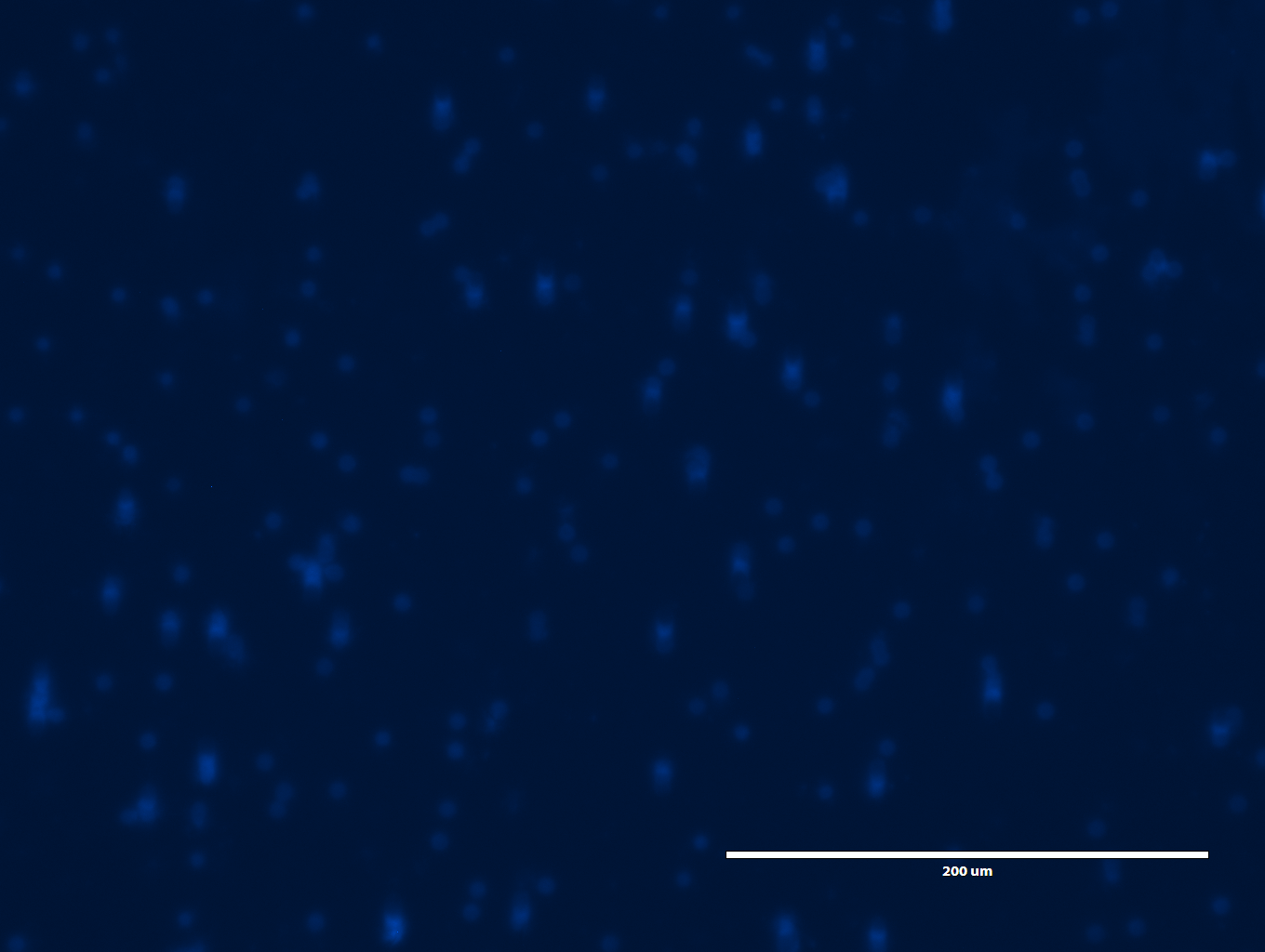

Supplement: Supplementary file 14 — Source data Fig. 6 [file 44319_2025_659_MOESM14_ESM.zip › Figure 6/6D/SourcehelaTSC2KO-CRISPRTFE3L.tif]

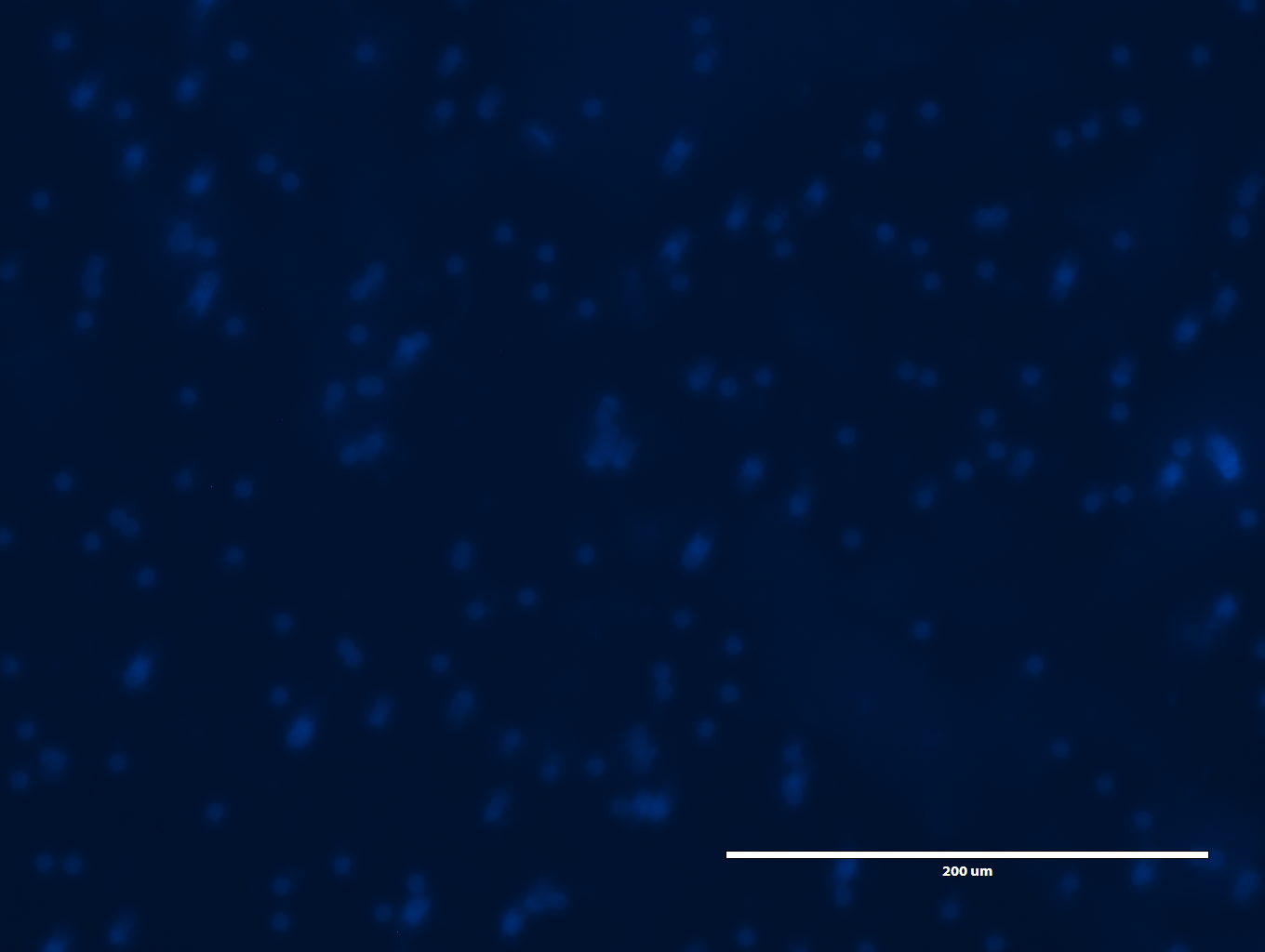

Supplement: Supplementary file 14 — Source data Fig. 6 [file 44319_2025_659_MOESM14_ESM.zip › Figure 6/6D/SourcehelaWT-CRISPRctrl.tif]
