# Supplementary material for: Differential contribution of TFE3 isoforms to cell motility and invasion
Source: EMBO Rep. 2025 Dec 8;27(2):471–500. doi: 10.1038/s44319-025-00659-3 (PMC12852735; doi:10.1038/s44319-025-00659-3)

Source Data. Figure 6C

HeLa WT
N-Cad
Hoechst

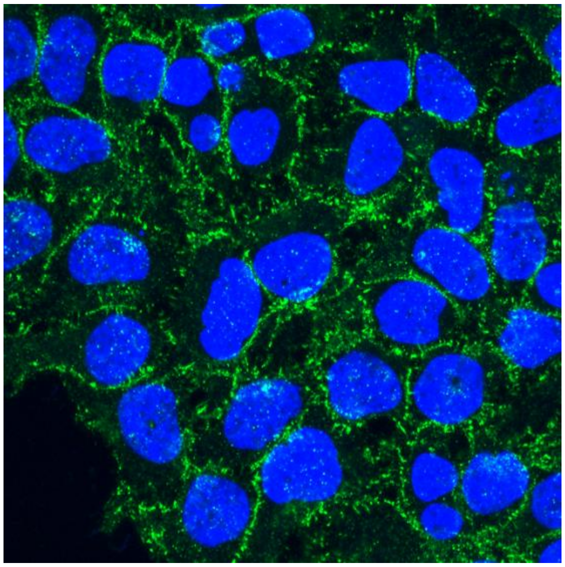

HeLa TSC2KO
CRISPR CTRL
N-Cad
Hoechst

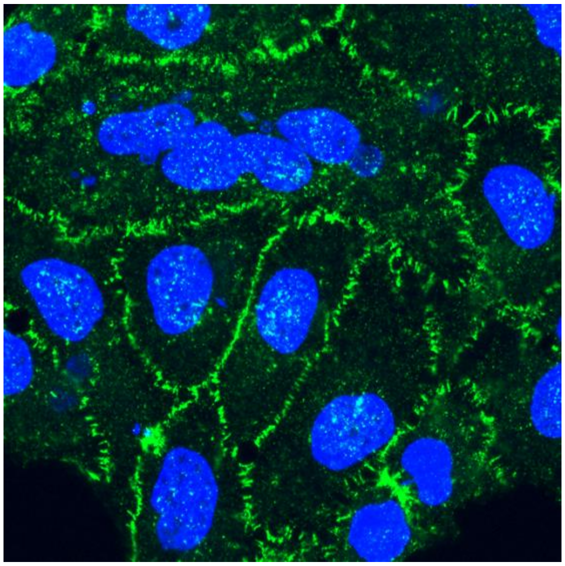

HeLa TSC2KO
TFE3-L CRISPR
N-Cad
Hoechst

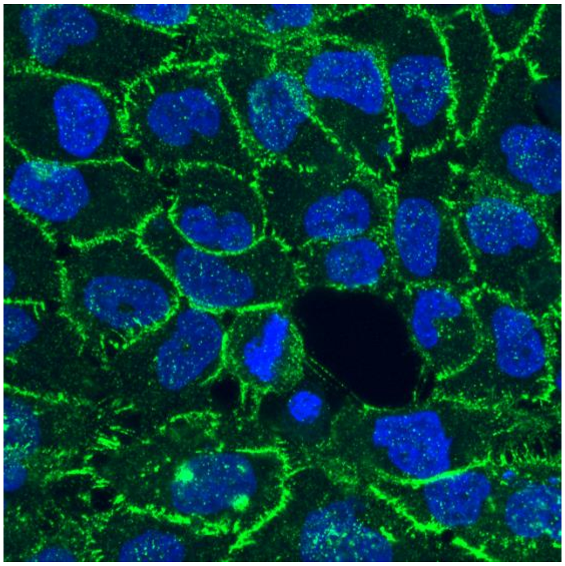

Supplement: Supplementary file 14 — Source data Fig. 6 [file 44319_2025_659_MOESM14_ESM.zip › Figure 6/6C/Source Data Fig 6C.pdf]
