# Supplementary material for: Differential contribution of TFE3 isoforms to cell motility and invasion
Source: EMBO Rep. 2025 Dec 8;27(2):471–500. doi: 10.1038/s44319-025-00659-3 (PMC12852735; doi:10.1038/s44319-025-00659-3)
Supplement: Supplementary file 14 — Source data Fig. 6 [file 44319_2025_659_MOESM14_ESM.zip › Figure 6/6D/Source Data Fig 6D.pdf]

Source Data. Figure 6C

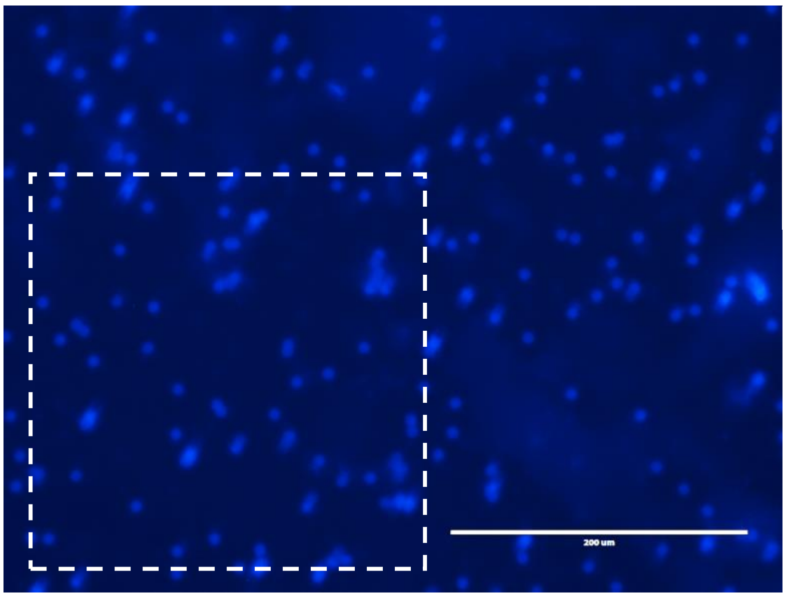

HeLa WT
CRISPR CTRL
Hoechst

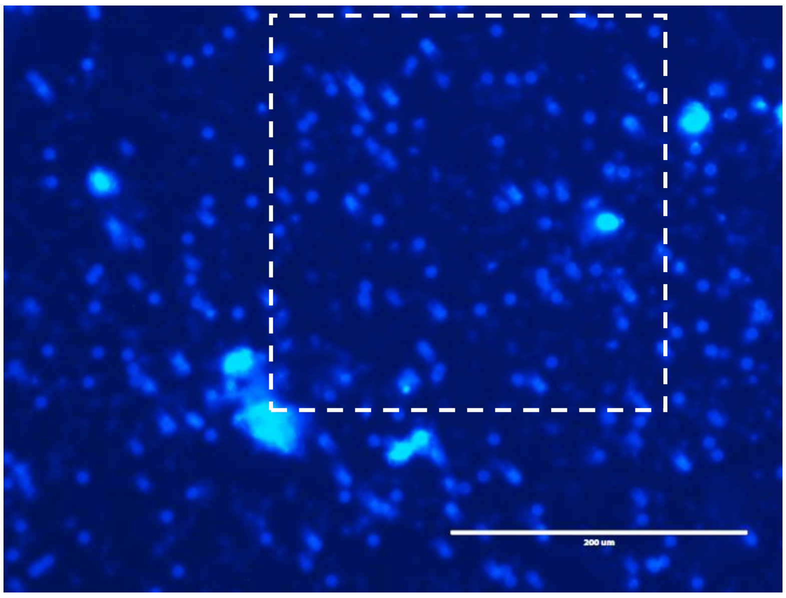

HeLa TSC2KO
CRISPR CTRL
Hoechst

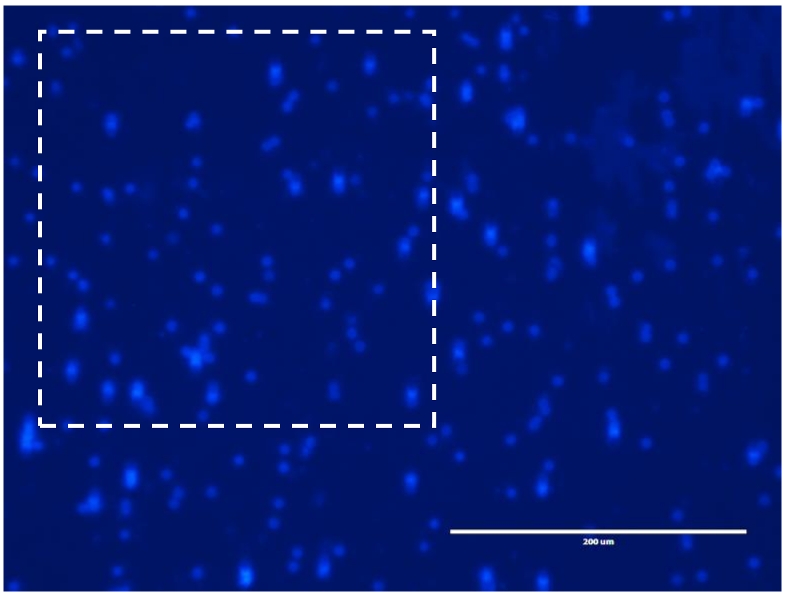

HeLa TSC2KO
CRISPR TFE3-L
Hoechst
