# Supplementary material for: Differential contribution of TFE3 isoforms to cell motility and invasion
Source: EMBO Rep. 2025 Dec 8;27(2):471–500. doi: 10.1038/s44319-025-00659-3 (PMC12852735; doi:10.1038/s44319-025-00659-3)
Supplement: Supplementary file 15 — Expanded View Figures [file 44319_2025_659_MOESM15_ESM.pdf]

Expanded View Figures

Figure EV1. Expression and activation of TFE3 isoforms in response to stress.

(**A**) Immunofluorescence confocal microscopy of ARPE19 cells showing the subcellular distribution of TFE3 (red) in response to treatment with EBSS for 1, 3, 8, 12, and 24 h. Scale bars: 10 μm. (**B**) Immunoblot analysis of protein lysates from HEK293T cells treated with EBSS for 1, 3, 8, 12, and 24 h. (**C**) Quantification of protein levels showing TFE3-L/TFE3-S ratio expressed as fold change as shown in (**B**). Data are presented as mean ± SD of four independent experiments. (ns) not significant $^{(a)}P = 0.9303$; (ns) not significant $^{(b)}P = 0.0981$; $^{**(c)}P = 0.0016$; $^{****(d)}P < 0.0001$; $^{****(e)}P < 0.0001$ (one-way ANOVA followed by Dunnett's multiple comparison post-test). (**D**) Quantification of protein levels showing TFE3-L/GAPDH ratio expressed as fold change as shown in (B). Data are presented as mean ± SD of four independent experiments. (ns) not significant $^{(a)}P = 0.9980$; (ns) not significant $^{(b)}P = 0.8448$; $^{*(c)}P = 0.0343$; $^{*(d)}P = 0.0315$; $^{**(e)}P = 0.0087$ (one-way ANOVA followed by Dunnett's multiple comparison post-test). (**E**) Immunoblot analysis of protein lysates from ARPE19 cells transiently expressing either active or inactive Rag heterodimers for 24 h and treated with 1 μM MLN4924 for 8 h. (**F**) Quantification of protein levels showing TFE3-L/TFE3 ratio expressed as fold change as shown in (**E**). Data are presented as mean ± SD of four independent experiments. $^{***(a)}P = 0.0005$; $^{***(b)}P = 0.0002$; $^{****(c)}P < 0.0001$ (one-way ANOVA followed by Dunnett's multiple comparison post-test). (**G**) Immunoblot analysis of protein lysates from HEK293T cells transiently expressing either active or inactive Rag heterodimers for 24 h. (**H**) Quantification of protein levels showing TFE3-L/TFE3 ratio expressed as fold change as shown in (**G**). Data are presented as mean ± SD of three independent experiments. $^{**}P = 0.0027$ (unpaired Student's *t* test). (**I**) Immunofluorescence confocal microscopy of RAW 264.7 cells showing the subcellular distribution of TFE3 (green) in response to treatment with 1 μg/ml LPS for 6, 12, 24, and 48 h. Scale bars: 10 μm. (**J**) Immunoblot analysis of protein lysates from RAW 264.7 cells treated with 1 μg/ml LPS for 6, 12, 24, and 48 h.

This is a U.S. Government work and not under copyright protection in the US; foreign copyright protection may apply

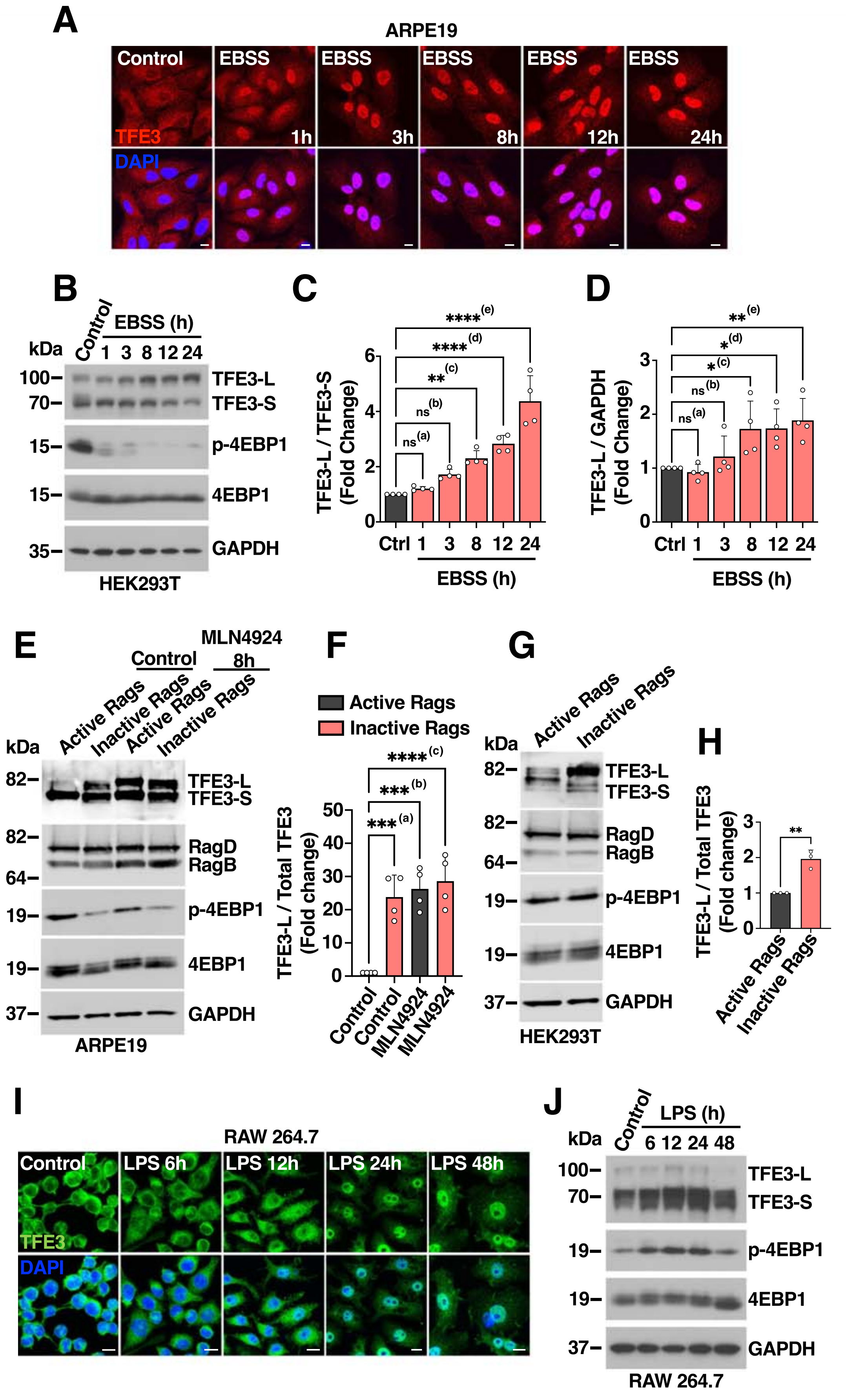

 This is a U.S. Government work and not under copyright protection in the US; foreign copyright protection may apply

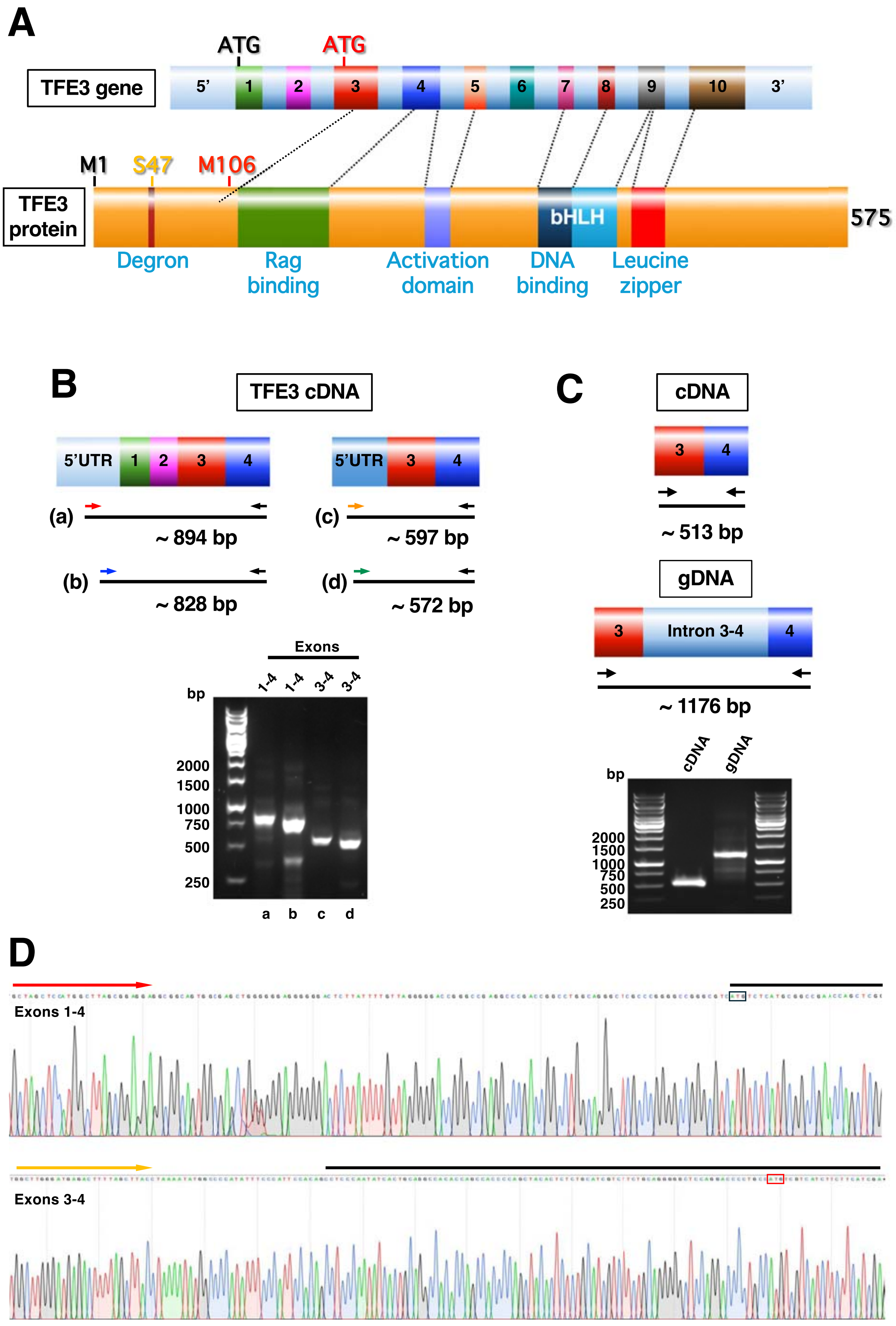
A
ATG
ATG
TFE3 gene
5'
1
2
3
4
5
6
7
8
9
10
3'
M1
S47
M106
TFE3 protein
bHLH
575
Degron
Rag binding
Activation domain
DNA binding
Leucine zipper
B
TFE3 cDNA
5'UTR 1 2 3 4
5'UTR 3 4
(a)
~ 894 bp
(b)
~ 828 bp
(c)
~ 597 bp
(d)
~ 572 bp
Exons
1-4
1-4
3-4
3-4
bp
2000
1500
1000
750
500
250
a b c d
C
cDNA
3 4
~ 513 bp
gDNA
3 Intron 3-4 4
~ 1176 bp
cDNA
gDNA
bp
2000
1500
1000
750
500
250
D
Exons 1-4
Exons 3-4

Figure EV2. TFE3-L and TFE3-S are products of two distinct TFE3 transcripts.

(**A**) Schematic diagram of the genomic structure of TFE3 representing exons 1 through 10, their corresponding intronic regions and the position of two start codons. The dotted lines indicate the region of exons encoding for the conserved functional domains of TFE3 protein. Intron sizes are not to scale, and exons and protein domains sizes and boundaries are approximate. (**B**) Gel electrophoresis analysis showing the RT-PCR amplified cDNA fragments of TFE3. Black arrows represent a common reverse primer targeting exon 4, red and blue arrows represent forward primers that target the 5′UTR of TFE3-L; orange and green arrows indicate primers to the intronic region upstream TFE3 exon 3. (**C**) Gel electrophoresis analysis showing amplified PCR fragments of TFE3 from either cDNA or genomic DNA (gDNA) using forward and reverse primers (black arrows) targeting exons 3 and 4, respectively. (**D**) Partial sequence chromatogram of the subcloned cDNA amplified fragments (a) and (c) shown in (**B**). Black lines indicate the beginning of the coding sequences for exons 1 and 3, and the start codons corresponding to methionines 1 and 106 are indicated with black and red rectangles, respectively.

 This is a U.S. Government work and not under copyright protection in the US; foreign copyright protection may apply

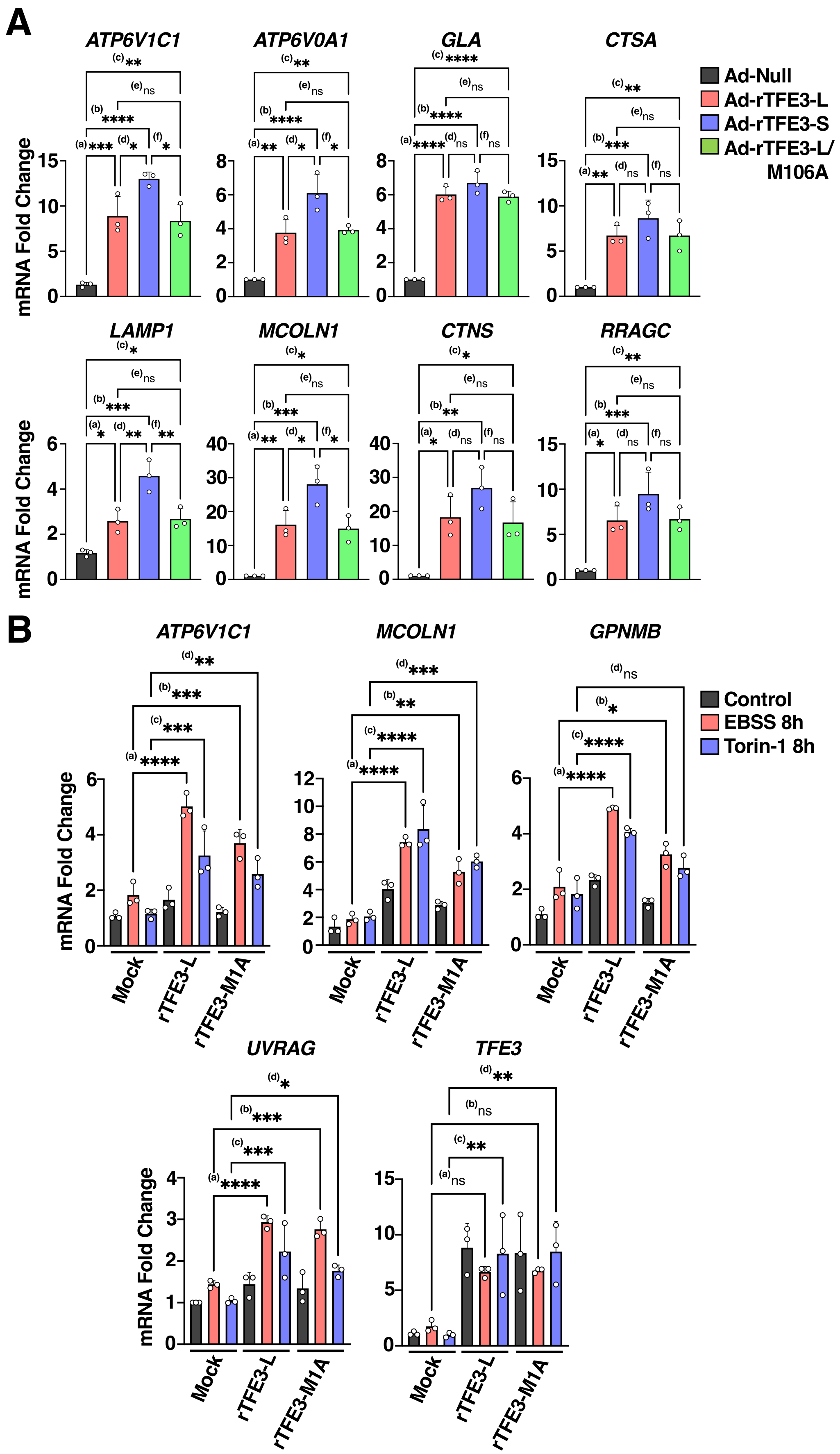
A
ATP6V1C1
ATP6V0A1
GLA
CTSA
LAMP1
MCOLN1
CTNS
RRAGC
mRNA Fold Change
Ad-Null
Ad-rTFE3-L
Ad-rTFE3-S
Ad-rTFE3-L/
M106A
B
ATP6V1C1
MCOLN1
GPNMB
UVRAG
TFE3
Control
EBSS 8h
Torin-1 8h
Mock
rTFE3-L
rTFE3-M1A

Figure EV3. Overexpression of recombinant TFE3-L-Myc, TFE3-S-Myc, and TFE3-L-M106A-Myc induces expression of lysosomal genes.

(**A**) Relative quantitative RT-PCR analysis of the mRNA expression of lysosomal genes in ARPE19 cells infected with adenovirus expressing recombinant TFE3-L-Myc, TFE3-S-Myc, TFE3-L/M106A-Myc or control adenovirus (Null) for 30 h. Data are presented as mean ± SD of three independent experiments. ATP6V1C1 ($^{***(a)}P = 0.0010$; $^{****(b)}P < 0.0001$; $^{**(c)}P = 0.0016$; $^{*(d)}P = 0.0352$; (ns) not significant $^{(e)}P = 0.9696$; $^{*(f)}P = 0.0192$), ATP6V0A1 ($^{**(a)}P = 0.0053$; $^{****(b)}P < 0.0001$; $^{**(c)}P = 0.0038$; $^{*(d)}P = 0.0144$; (ns) not significant $^{(e)}P = 0.9923$; $^{*(f)}P = 0.0207$), GLA ($^{****(a)}P < 0.0001$; $^{****(b)}P < 0.0001$; $^{****(c)}P < 0.0001$; (ns) not significant $^{(d)}P = 0.3036$; (ns) not significant $^{(e)}P = 0.9836$; (ns) not significant $^{(f)}P = 0.1908$), CTSA ($^{**(a)}P = 0.0049$; $^{***(b)}P = 0.0008$; $^{**(c)}P = 0.0049$; (ns) not significant $^{(d)}P = 0.4090$; (ns) not significant $^{(e)}P > 0.9999$; (ns) not significant $^{(f)}P = 0.4057$), LAMP1 ($^{*(a)}P = 0.0363$; $^{***(b)}P = 0.0002$; $^{*(c)}P = 0.0254$; $^{*(d)}P = 0.0054$; (ns) not significant $^{(e)}P = 0.9936$; $^{*(f)}P = 0.0074$), MCOLN1 ($^{**(a)}P = 0.0075$; $^{***(b)}P = 0.0002$; $^{*(c)}P = 0.0118$; $^{*(d)}P = 0.0279$; (ns) not significant $^{(e)}P = 0.9842$; $^{*(f)}P = 0.0173$), CTNS ($^{*(a)}P = 0.0164$; $^{**(b)}P = 0.0014$; $^{*(c)}P = 0.0266$; (ns) not significant $^{(d)}P = 0.2641$; (ns) not significant $^{(e)}P = 0.9835$; (ns) not significant $^{(f)}P = 0.1642$), RRAGC ($^{*(a)}P = 0.0113$; $^{***(b)}P = 0.0008$; $^{**(c)}P = 0.0099$; (ns) not significant $^{(d)}P = 0.1882$; (ns) not significant $^{(e)}P = 0.9996$; (ns) not significant $^{(f)}P = 0.2160$) (one-way ANOVA followed by Tukey's multiple comparison post-test). (**B**) Relative quantitative RT-PCR analysis of the mRNA expression of lysosomal and autophagy genes in ARPE19 cells transiently expressing recombinant TFE3-L-Myc or TFE3-L/M1A-Myc for 24 h and treated with either EBSS or 250 nM Torin-1 for 8 h. Data are presented as mean ± SD of three independent experiments. ATP6V1C1 ($^{****(a)}P < 0.0001$; $^{**(b)}P = 0.0008$; $^{****(c)}P = 0.0002$; $^{**(d)}P = 0.0100$), MCOLN1 ($^{****(a)}P < 0.0001$; $^{**(b)}P = 0.0012$; $^{****(c)}P < 0.0001$; $^{***(d)}P = 0.0002$), GPNMB ($^{****(a)}P < 0.0001$; $^{*(b)}P = 0.0114$; $^{****(c)}P < 0.0001$; (ns) not significant $^{(d)}P = 0.0526$), UVRAG ($^{****(a)}P < 0.0001$; $^{***(b)}P = 0.0001$; $^{***(c)}P = 0.0005$; $^{*(d)}P = 0.0433$), TFE3 ((ns) not significant $^{(a)}P = 0.0649$; (ns) not significant $^{(b)}P = 0.0560$; $^{**(c)}P = 0.0026$; $^{**(d)}P = 0.0020$) (two-way ANOVA followed by Tukey's multiple comparison post-test).

 This is a U.S. Government work and not under copyright protection in the US; foreign copyright protection may apply

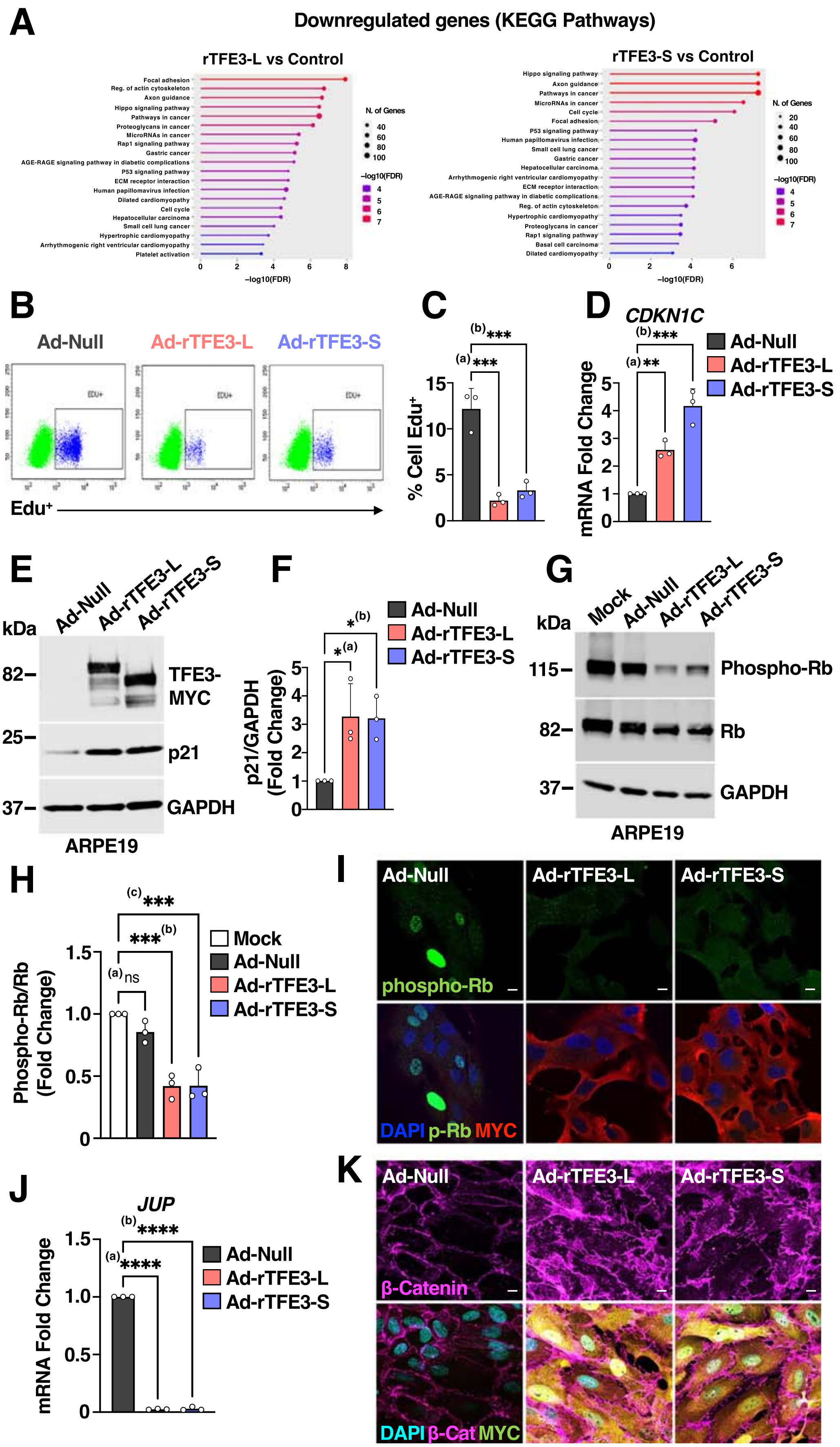
A
Downregulated genes (KEGG Pathways)
rTFE3-L vs Control
rTFE3-S vs Control
N. of Genes
−log10(FDR)
B
Ad-Null
Ad-rTFE3-L
Ad-rTFE3-S
Edu+
C
% Cell Edu+
D
CDKN1C
mRNA Fold Change
E
kDa
TFE3-MYC
p21
GAPDH
ARPE19
F
p21/GAPDH (Fold Change)
G
Mock
Phospho-Rb
Rb
ARPE19
H
Phospho-Rb/Rb (Fold Change)
I
phospho-Rb
DAPI p-Rb MYC
J
JUP
K
β-Catenin
DAPI β-Cat MYC

Figure EV4. TFE3-L-Myc and TFE3-S-Myc overexpression reduces proliferation and alters cell–cell contacts.

(**A**) KEGG pathway enrichment analysis of genes downregulated by TFE3-L-Myc or TFE3-S-Myc. Circle size represents the number of genes, while the color scale indicates the -log10 of the false discovery rate (FDR). (**B**) Flow cytometry analysis of 5-ethynyl-2′-deoxyuridine (EdU) incorporation in ARPE19 cells infected with control adenovirus (Null) or adenovirus expressing recombinant TFE3-L-Myc and TFE3-S-Myc for 30 h. (**C**) Quantification of the percentage of infected ARPE19 cells with incorporated EdU as shown in (**B**). Data are presented as mean ± SD of three independent experiments. $^{***(a)}P = 0.0003$; $^{***(b)}P = 0.0005$ (one-way ANOVA followed by Dunnett's multiple comparison post-test). (**D**) Relative quantitative RT-PCR analysis of the mRNA expression of CDKN1C gene in ARPE19 cells infected with adenovirus expressing recombinant TFE3-L-Myc, TFE3-S-Myc or Control for 30 h. Data are presented as mean ± SD of three independent experiments. $^{**(a)}P = 0.0052$; $^{***(b)}P = 0.0001$ (one-way ANOVA followed by Dunnett's multiple comparison post-test). (**E**) Immunoblot analysis of protein lysates from ARPE19 cells infected with adenovirus expressing recombinant TFE3-L-Myc, TFE3-S-Myc, or control adenovirus (Null) for 30 h. (**F**) Quantification of protein levels showing p21/GAPDH ratio expressed as fold change as shown in (**E**). Data are presented as mean ± SD of three independent experiments. $^{*(a)}P = 0.0234$; $^{*(b)}P = 0.0262$ (one-way ANOVA followed by Dunnett's multiple comparison post-test). (**G**) Immunoblot analysis of protein lysates from ARPE19 cells infected with adenovirus expressing recombinant TFE3-L-Myc, TFE3-S-Myc, or control adenovirus (Null) for 30 h. (**H**) Quantification of protein levels showing phospho-Rb/Rb ratio expressed as fold change as shown in (**G**). Data are presented as mean ± SD of three independent experiments. (ns) not significant $^{(a)}P = 0.2001$; $^{***(b)}P = 0.0001$; $^{***(c)}P = 0.0001$ (one-way ANOVA followed by Dunnett's multiple comparison post-test). (**I**) Immunofluorescence confocal microscopy of ARPE19 cells infected with adenovirus expressing recombinant TFE3-L-Myc, TFE3-S-Myc, or control adenovirus (Null) for 30 h, showing the cellular distribution of phospho-Rb (green) and recombinant TFE3-L-Myc or TFE3-S-Myc (red). Scale bars: 10 µm. (**J**) Relative quantitative RT-PCR analysis of the mRNA expression of *JUP* in ARPE19 cells infected with adenovirus expressing recombinant TFE3-L-Myc, TFE3-S-Myc, or control adenovirus (Null) for 30 h. Data are presented as mean ± SD of three independent experiments. $^{****(a)}P < 0.0001$; $^{****(b)}P < 0.0001$ (one-way ANOVA followed by Dunnett's multiple comparison post-test). (**K**) Immunofluorescence confocal microscopy of ARPE19 cells infected with the indicated adenovirus for 30 h, showing cellular distribution of β-Catenin (pseudo-color magenta) and recombinant TFE3-L-Myc or TFE3-S-Myc (green). Scale bars: 10 µm.

This is a U.S. Government work and not under copyright protection in the US; foreign copyright protection may apply

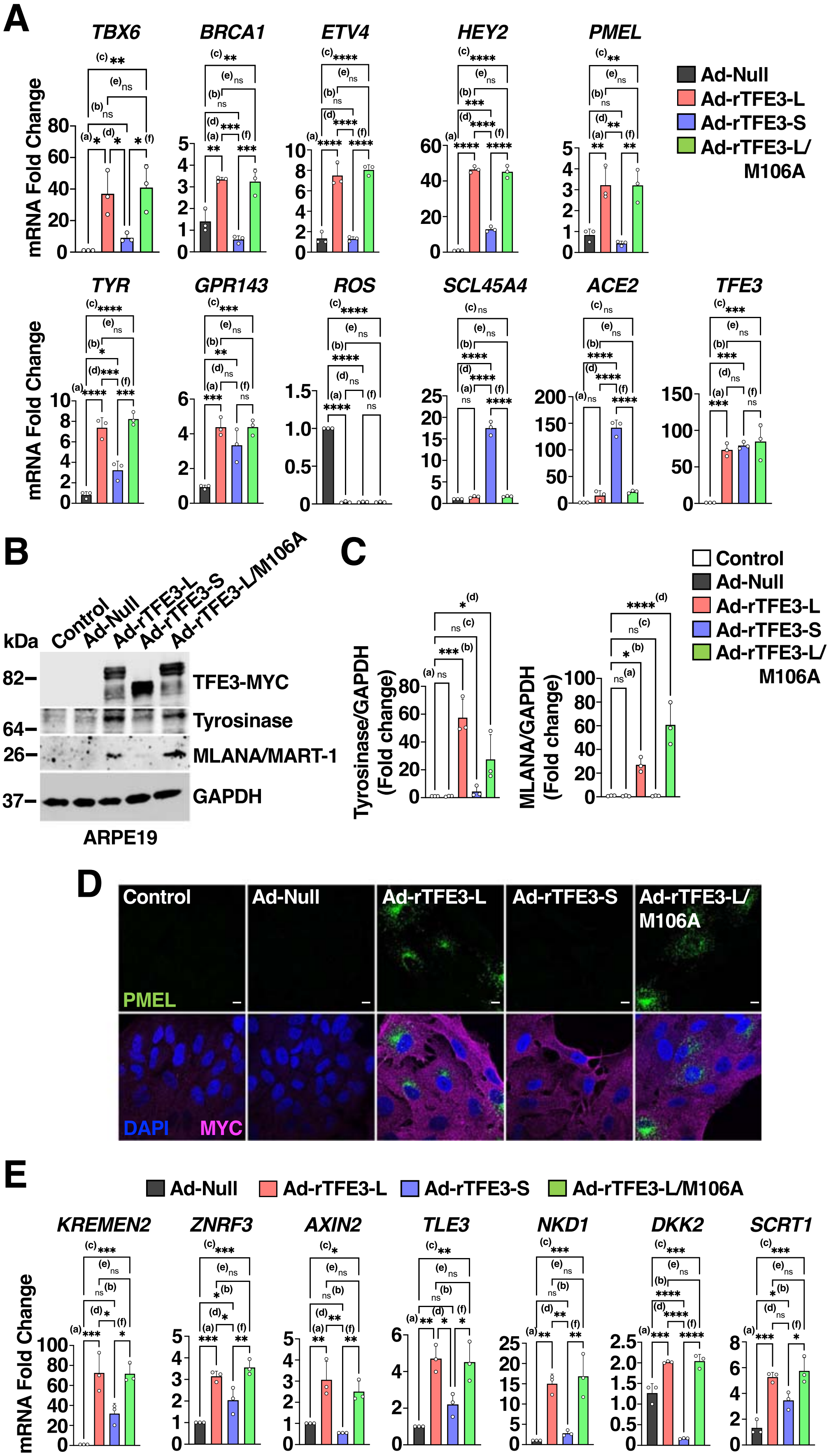
A
TBX6
BRCA1
ETV4
HEY2
PMEL
mRNA Fold Change
Ad-Null
Ad-rTFE3-L
Ad-rTFE3-S
Ad-rTFE3-L/M106A
TYR
GPR143
ROS
SCL45A4
ACE2
TFE3
B
kDa
Control
Ad-Null
Ad-rTFE3-L
Ad-rTFE3-S
Ad-rTFE3-L/M106A
82–
64–
26–
37–
TFE3-MYC
Tyrosinase
MLANA/MART-1
GAPDH
ARPE19
C
Tyrosinase/GAPDH (Fold change)
MLANA/GAPDH (Fold change)
Control
Ad-Null
Ad-rTFE3-L
Ad-rTFE3-S
Ad-rTFE3-L/M106A
D
Control
Ad-Null
Ad-rTFE3-L
Ad-rTFE3-S
Ad-rTFE3-L/M106A
PMEL
DAPI
MYC
E
Ad-Null
Ad-rTFE3-L
Ad-rTFE3-S
Ad-rTFE3-L/M106A
KREMEN2
ZNRF3
AXIN2
TLE3
NKD1
DKK2
SCRT1
mRNA Fold Change

Figure EV5. Differential gene expression upon overexpression of TFE3 isoforms.

(**A**) Relative quantitative RT-PCR analysis of the mRNA expression of the indicated genes in ARPE19 cells infected with adenovirus expressing recombinant TFE3-L-Myc, TFE3-S-Myc, TFE3-L/M106A-Myc or control adenovirus (Null) for 30 h. Data are presented as mean ± SD of three independent experiments. TBX6 ($^{*(a)}P = 0.0110$; (ns) not significant $^{(b)}P = 0.7678$; $^{**(c)}P = 0.0060$; $^{*(d)}P = 0.0412$; (ns) not significant $^{(e)}P = 0.9619$; $^{*(f)}P = 0.0213$), BRCA1 ($^{**(a)}P = 0.0017$; (ns) not significant $^{(b)}P = 0.1346$; $^{**(c)}P = 0.0024$; $^{***(d)}P = 0.0001$; (ns) not significant $^{(e)}P = 0.9899$; $^{***(f)}P = 0.0002$), ETV4 ($^{****(a)}P < 0.0001$; (ns) not significant $^{(b)}P = 0.9998$; $^{****(c)}P < 0.0001$; $^{****(d)}P < 0.0001$; (ns) not significant $^{(e)}P = 0.7658$; $^{****(f)}P < 0.0001$), HEY2 ($^{****(a)}P < 0.0001$; $^{***(b)}P = 0.0005$; $^{****(c)}P < 0.0001$; $^{****(d)}P < 0.0001$; (ns) not significant $^{(e)}P = 0.8636$; $^{****(f)}P < 0.0001$), PMEL ($^{**(a)}P = 0.0049$; (ns) not significant $^{(b)}P = 0.8388$; $^{**(c)}P = 0.0050$; $^{**(d)}P = 0.0018$; (ns) not significant $^{(e)}P = 0.5424$; $^{**(f)}P = 0.0019$), TYR ($^{****(a)}P < 0.0001$; $^{*(b)}P = 0.0179$; $^{****(c)}P < 0.0001$; $^{***(d)}P = 0.0006$; (ns) not significant $^{(e)}P = 0.7658$; $^{***(f)}P = 0.0002$), GPR143 ($^{***(a)}P = 0.0004$; $^{**(b)}P = 0.0046$; $^{***(c)}P = 0.0004$; (ns) not significant $^{(d)}P = 0.2124$; (ns) not significant $^{(e)}P > 0.9999$; (ns) not significant $^{(f)}P = 0.2084$), ROS ($^{****(a)}P < 0.0001$; $^{****(b)}P < 0.0001$; $^{****(c)}P < 0.0001$; (ns) not significant $^{(d)}P > 0.9999$; (ns) not significant $^{(e)}P = 0.9868$; (ns) not significant $^{(f)}P = 0.9868$), SCL45A4 ((ns) not significant $^{(a)}P = 0.8487$; $^{****(b)}P < 0.0001$; (ns) not significant $^{(c)}P = 0.7804$; $^{****(d)}P < 0.0001$; (ns) not significant $^{(e)}P = 0.9989$; $^{****(f)}P < 0.0001$), ACE2 ((ns) not significant $^{(a)}P = 0.3170$; $^{****(b)}P < 0.0001$; (ns) not significant $^{(c)}P = 0.0831$; $^{****(d)}P < 0.0001$; (ns) not significant $^{(e)}P = 0.7610$; $^{****(f)}P < 0.0001$), TFE3 ($^{***(a)}P = 0.0006$; $^{***(b)}P = 0.0004$; $^{***(c)}P = 0.0002$; (ns) not significant $^{(d)}P = 0.9410$; (ns) not significant $^{(e)}P = 0.7137$; (ns) not significant $^{(f)}P = 0.9535$) (one-way ANOVA followed by Tukey's multiple comparison post-test). (**B**) Immunoblot analysis of protein lysates from ARPE19 cells infected with adenovirus expressing recombinant TFE3-L-Myc, TFE3-S-Myc, TFE3-L/M106A-Myc or control adenovirus (Null) for 30 h. (**C**) Quantification of protein levels showing Tyrosinase/GAPDH and MLANA/GAPDH ratios expressed as fold change as shown in (**B**). Data are presented as mean ± SD of three independent experiments. Tyrosinase ((ns) not significant $^{(a)}P > 0.9999$; $^{***(b)}P = 0.0002$; (ns) not significant $^{(c)}P = 0.9798$; $^{*(d)}P = 0.0291$), MLANA ((ns) not significant $^{(a)}P > 0.9999$; $^{*(b)}P = 0.0115$; (ns) not significant $^{(c)}P > 0.9999$; $^{****(d)}P < 0.0001$) (one-way ANOVA followed by Dunnett's multiple comparison post-test). (**D**) Immunofluorescence confocal microscopy of ARPE19 cells infected with the indicated adenovirus for 30 h, showing the cellular distribution of PMEL (green) and recombinants TFE3-Myc (pseudo-color magenta). Scale bars: 10 µm. (**E**) Relative quantitative RT-PCR analysis of the mRNA expression of negative regulators of Wnt pathway genes (*KREMEN2*, *ZNRF3*, *AXIN2*, *TLE3*, *NKD1*, *DKK2*, and *SCRT1*) in ARPE19 cells infected with adenovirus expressing recombinant TFE3-L-Myc, TFE3-S-Myc, TFE3-L/M106A-Myc or control adenovirus (Null) for 30 h. Data are presented as mean ± SD of three independent experiments. KREMEN2 ($^{***(a)}P = 0.0004$; (ns) not significant $^{(b)}P = 0.0515$; $^{***(c)}P = 0.0004$; $^{*(d)}P = 0.0128$; (ns) not significant $^{(e)}P = 0.9998$; $^{*(f)}P = 0.0143$), ZNRF3 ($^{***(a)}P = 0.0005$; $^{*(b)}P = 0.0367$; $^{***(c)}P = 0.0001$; $^{*(d)}P = 0.0274$; (ns) not significant $^{(e)}P = 0.5627$; $^{**(f)}P = 0.0047$), AXIN2 ($^{**(a)}P = 0.0043$; (ns) not significant $^{(b)}P = 0.6984$; $^{*(c)}P = 0.0251$; $^{**(d)}P = 0.0012$; (ns) not significant $^{(e)}P = 0.5537$; $^{**(f)}P = 0.0059$), TLE3 ($^{**(a)}P = 0.0011$; (ns) not significant $^{(b)}P = 0.2473$; $^{**(c)}P = 0.0015$; (ns) not significant $^{(d)}P = 0.0122$; (ns) not significant $^{(e)}P = 0.9882$; $^{*(f)}P = 0.0185$), NKD1 ($^{**(a)}P = 0.0017$; (ns) not significant $^{(b)}P = 0.8730$; $^{***(c)}P = 0.0008$; $^{*(d)}P = 0.0042$; (ns) not significant $^{(e)}P = 0.8692$; $^{**(f)}P = 0.0017$), DKK2 ($^{***(a)}P = 0.0009$; $^{****(b)}P < 0.0001$; $^{***(c)}P = 0.0007$; $^{****(d)}P < 0.0001$; (ns) not significant $^{(e)}P = 0.9964$; $^{****(f)}P < 0.0001$), SCRT1 ($^{***(a)}P = 0.0006$; $^{*(b)}P = 0.0251$; $^{***(c)}P = 0.0003$; (ns) not significant $^{(d)}P = 0.0543$; (ns) not significant $^{(e)}P = 0.8366$; $^{*(f)}P = 0.0171$) (one-way ANOVA followed by Tukey's multiple comparison post-test).

This is a U.S. Government work and not under copyright protection in the US; foreign copyright protection may apply
